# Super interactive promoters provide insight into cell type-specific regulatory networks in blood lineage cell types

**DOI:** 10.1371/journal.pgen.1009984

**Published:** 2022-01-31

**Authors:** Jia Wen, Taylor M. Lagler, Quan Sun, Yuchen Yang, Jiawen Chen, Yuriko Harigaya, Vijay G. Sankaran, Ming Hu, Alexander P. Reiner, Laura M. Raffield, Yun Li

**Affiliations:** 1 Department of Genetics, University of North Carolina at Chapel Hill, Chapel Hill, North Carolina, United States of America; 2 Department of Biostatistics, University of North Carolina at Chapel Hill, Chapel Hill, North Carolina, United States of America; 3 Department of Pathology and Laboratory Medicine, University of North Carolina at Chapel Hill, Chapel Hill, North Carolina, United States of America; 4 McAllister Heart Institute, University of North Carolina at Chapel Hill, Chapel Hill, North Carolina, United States of America; 5 State Key Laboratory of Biocontrol, School of Ecology, Sun Yat-sen University, Guangzhou, Guangdong, China; 6 Curriculum in Bioinformatics and Computational Biology, University of North Carolina at Chapel Hill, Chapel Hill, North Carolina, United States of America; 7 Division of Hematology/Oncology, Boston Children’s Hospital and Department of Pediatric Oncology, Dana-Farber Cancer Institute, Harvard Medical School, Boston, Massachusetts, United States of America; 8 Broad Institute of MIT and Harvard, Cambridge, Massachusetts, United States of America; 9 Harvard Stem Cell Institute, Cambridge, Massachusetts, United States of America; 10 Department of Quantitative Health Sciences, Lerner Research Institute, Cleveland Clinic Foundation, Cleveland, Ohio, United States of America; 11 Division of Public Health Sciences, Fred Hutchinson Cancer Research Center, Seattle, Washington, United States of America; 12 Department of Epidemiology, University of Washington, Seattle, Washington, United States of America; 13 Department of Computer Science, University of North Carolina at Chapel Hill, Chapel Hill, North Carolina, United States of America; University of Pennsylvania, UNITED STATES

## Abstract

Existing studies of chromatin conformation have primarily focused on potential enhancers interacting with gene promoters. By contrast, the interactivity of promoters per se, while equally critical to understanding transcriptional control, has been largely unexplored, particularly in a cell type-specific manner for blood lineage cell types. In this study, we leverage promoter capture Hi-C data across a compendium of blood lineage cell types to identify and characterize cell type-specific super-interactive promoters (SIPs). Notably, promoter-interacting regions (PIRs) of SIPs are more likely to overlap with cell type-specific ATAC-seq peaks and GWAS variants for relevant blood cell traits than PIRs of non-SIPs. Moreover, PIRs of cell-type-specific SIPs show enriched heritability of relevant blood cell trait (s), and are more enriched with GWAS variants associated with blood cell traits compared to PIRs of non-SIPs. Further, SIP genes tend to express at a higher level in the corresponding cell type. Importantly, SIP subnetworks incorporating cell-type-specific SIPs and ATAC-seq peaks help interpret GWAS variants. Examples include GWAS variants associated with platelet count near the megakaryocyte SIP gene *EPHB3* and variants associated lymphocyte count near the native CD4 T-Cell SIP gene *ETS1*. Interestingly, around 25.7% ~ 39.6% blood cell traits GWAS variants residing in SIP PIR regions disrupt transcription factor binding motifs. Importantly, our analysis shows the potential of using promoter-centric analyses of chromatin spatial organization data to identify biologically important genes and their regulatory regions.

## Author summary

By analyzing pcHi-C data, we catalogue super-interactive promoters (SIPs) in five blood cell types. These SIPs and SIP genes in blood cells will be valuable not only for studying hematological traits but for many complex phenotypes. We provide mechanistic hypotheses regarding the formation of SIPs. To be identified as a SIP, a promoter can be driven by few super strong interactions or many significant (not necessarily all strong) interactions. Importantly, we find that the latter seems to be the norm. This finding sheds light regarding the formation of SIPs: to ensure the expression level of some critical gene (here a SIP gene), multiple regulatory regions are likely key for orchestrating fine transcriptional control. These multiple regulatory regions provide a level of “redundancy”, ensuring that even in the presence of genetic variant (s) that disrupt some enhancer(s), appropriate transcriptional regulation can still be maintained in a given hematopoietic cell type. This finding also has important implications for the interpretation and functional follow-up of hundreds of thousands of GWAS findings. These multiple regulatory regions for one SIP gene help explain multiple independent GWAS signals at one locus. In summary, we believe our work presents important findings governing the orchestrated transcriptional control in blood lineage cell types, and provides valuable insights and resources for the interpretation and follow-up of GWAS findings of many complex traits.

## Introduction

Genome-wide chromosome conformation capture techniques such as Hi-C [1] have been widely used to study chromatin three-dimensional (3D) organization. However, due to the complexity and sparsity of Hi-C data, it is difficult to identify statistically significant long-range chromatin interactions between distant genomic sequences at fine resolutions (e.g, at restriction fragment level, or < 10Kb equal size bin level) even with tens of billions of pairwise reads produced [2,3]. Furthermore, ultra-deep sequencing is costly and likely to generate redundant reads, leading to Hi-C library saturation [4]. In addition, chromatin spatial organization studies have largely focused on regulatory regions, but characterization of the 3D genome at promoters is also important for understanding gene expression regulation. To bridge this gap, capture Hi-C and subsequent variations were developed as an extension of the Hi-C technique by combining target enrichment and sequencing [5–8]. One such capture technique, promoter capture Hi-C (pcHi-C), was developed to focus on promoter regions. These regions have been largely taken for granted and automatically removed from detailed study in many chromatin conformation-based studies [9–11]. PcHi-C is specifically enriched for promoter sequences. Following Song et al 2020 [7], we define super interactive promoters (SIPs) as promoters with high cumulative chromatin interactions with other regions, while other promoters as non-SIPs. The cognate gene for a SIP, i.e., the gene whose promoter is the SIP, is called the SIP gene. PcHi-C also enables genome-wide detection of distal promoter-interacting regions (PIRs) for all promoters with *a priori* designed probes/baits in a single experiment [12]. In this manuscript, we refer to the PIRs interacting with SIPs as SIP PIRs and PIRs interacting with non-SIPs as non-SIP PIRs.

Promoter interactomes (the set of all interactions involving promoters within a cell) are tissue- and lineage-specific and have been used to link promoters to GWAS risk loci [9,12–14]. Consequently, there has been growing interest in studying cell type-specific differences in PIRs. As one example, pcHi-C analysis of 17 human hematopoietic cells demonstrated that PIRs are highly cell type-specific and reflective of the expected lineage relationships (such as mapping of promoter interactions for T-cell receptor component encoding genes to lymphoid cell types only, not to myeloid lineage cell types). Importantly, this analysis demonstrated the ability of pcHi-C to link non-coding regulatory variants to their target genes [13]. Thus, pcHi-C analysis can be leveraged to provide insight into gene expression control and the function of non-coding disease-associated sequence variants [12].

A recent study on human corticogenesis has identified a subset of promoters exhibiting unusually high degrees of chromatin interactivity (where chromatin interactivity is defined by cumulative CHiCAGO scores of interactions with neighboring regions), which were termed SIPs [7]. Song et al. reported that these brain cortex SIPs were enriched for the corresponding lineage-specific genes compared to non-SIPs, suggesting that the interactions between SIPs and their regulatory networks may play a role in modulating cell type-specific transcription. In addition, Song et al. also found SIPs in hematopoietic lineages using pcHi-C data, but did not perform further annotation or characterization of these hematopoietic SIPs.

Due to the relative ease of measuring blood cells, rich genomics data is available for hematopoietic cells. Further, different hematopoietic cell types play different roles in blood cell generation and function and correspond to different phenotypic traits (for example inflammation, autoimmunity, and infection phenotypes for white blood cell types, thrombosis and hemostasis related phenotypes for platelet producing megakaryocytes), emphasizing the importance of studying them in cell type-specific manner [15]. Blood cells are highly relevant tissues for many complex phenotypes, including infectious disease susceptibility (including COVID-19), disease related biomarkers such as telomere length or circulating inflammatory cytokines, thrombosis (including venous thromboembolism and stroke), asthma and other respiratory diseases, and autoimmune conditions [16]. Understanding of interactions of gene promoters and their regulatory regions in specific blood cell types, as opposed to simple analysis of “whole blood”, can lead to improved annotation of genome-wide association study (GWAS) identified loci and their target genes, and thus of the genetic mechanisms underlying complex disease risk. Hematopoietic SIPs are thus of broad interest for understanding gene regulation and its connection to disease risk in human populations.

Here, we focus on characterizing promoter-centric chromatin spatial interaction profiles, across a compendium of cell types in the hematopoietic lineage. In this study, we identify and characterize SIPs in human blood cells using pcHi-C data from the Javierre et al. study [13]. We first detect SIPs for multiple blood lineage cell types in a cell type specific manner, and then characterize SIPs, non-SIPs, SIP PIRs and non-SIP PIRs from multiple aspects including overlapping with cell-type-specific ATAC-seq peaks, enrichment of GWAS variants, blood cell traits heritability enrichment, relationship to gene expression levels, and the construction of SIP subnetworks. We find that SIPs tend to be either cell type-specific or shared across all cell types, in contrast to being shared by a number but not all cell types, similar to gene expression levels across tissues [17]. Through examining the differences between SIPs and non-SIPs in terms of their interaction profiles as well as their genes, we find that SIPs share common properties across cell types. Importantly, we demonstrate how studying SIP networks may provide insight into the complex regulation of promoters as well as potential functional interactions.

## Results

### Inequality in the promoter interactome: Few Super-Interactive promoters

**Fig 1:**
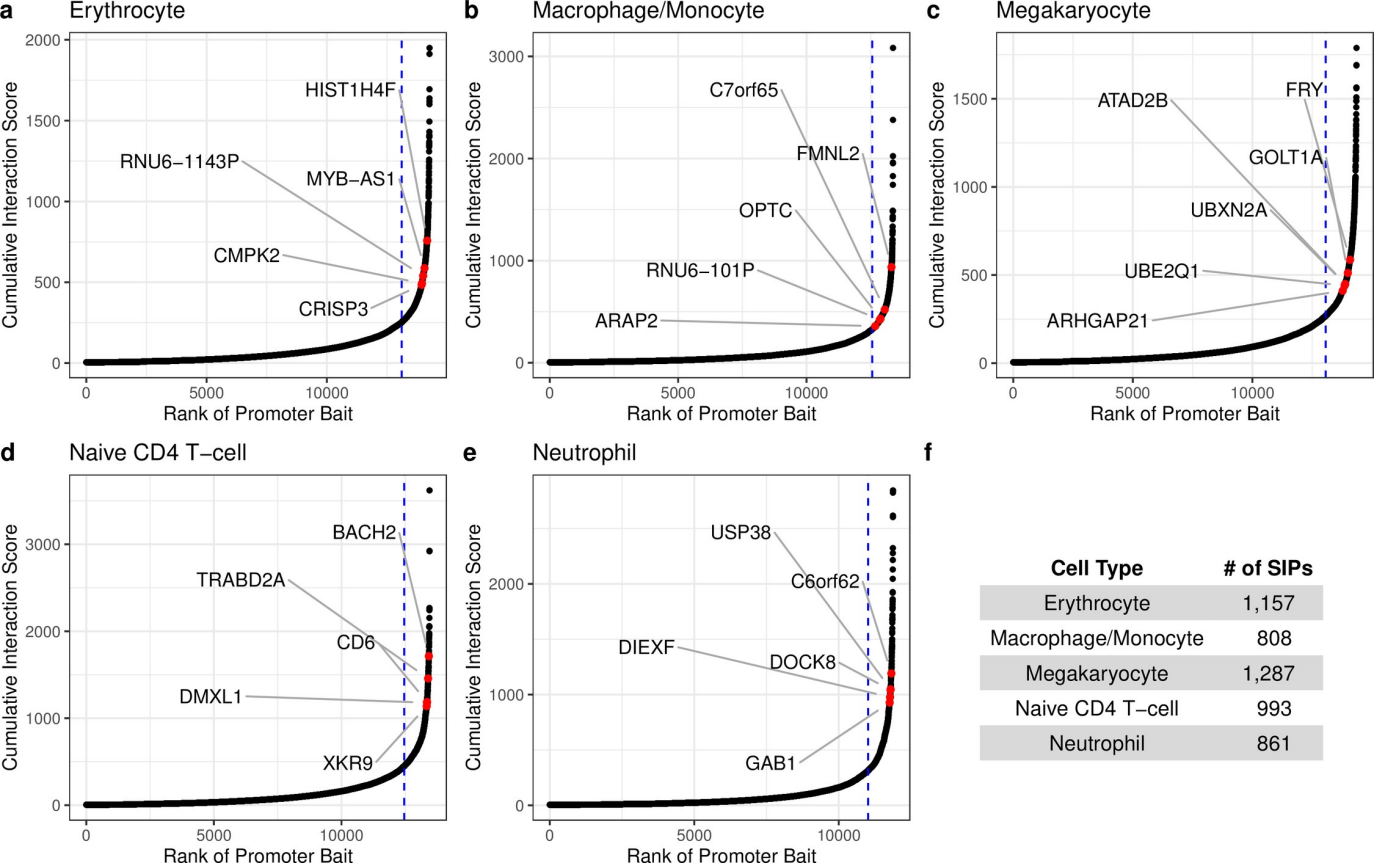
There are few super-interactive promoters (SIPs) in the chromatin interactome. **(A)-(E)** Hockey plots for each cell type show the ranked cumulative interaction scores for pcHi-C promoter-containing anchor bins (baits). Promoters to the right of the blue vertical line are classified as super interactive promoters (SIPs), as they exhibit unusually high levels of chromatin interactivity. Red dots highlight the highest ranked cell type-specific SIP genes^1^ with a PIR overlapping a relevant GWAS identified SNP. (**F**) Total number of SIPs annotated per cell type. ^1^The megakaryocyte genes *ATAD2B* and UBXN2A correspond to the same SIP bait.

We first examine the interactivity of promoters using pcHi-C data from Javierre at al. [13] in each of the five hematopoietic cell types: erythrocyte (Ery), macrophage/monocyte (MacMon), megakaryocyte (MK), naive CD4 T-cell (nCD4), and neutrophil (Neu) (see Methods). For each cell type, we ranked the promoter-containing anchor bins (baits) according to their cumulative interaction scores (see Methods) (**Fig 1**). We find that a small number of promoter baits (~7.5%) have extremely high cumulative interaction scores, as defined based on the curve inflection point in each cell type, and annotated them as super-interactive promoters (SIPs). In total, we annotate 1,157, 808, 1,287, 993, and 861 SIPs in erythrocytes, macrophages/monocytes, megakaryocytes, naive nCD4 T-cells, and neutrophils, respectively (**S1 Table**). These SIPs can be cell type-specific or shared across cell types. There are 170 SIPs shared across all five cell types, as well as 189, 107, 302, 283, and 274 cell type-specific SIPs in erythrocytes, macrophages/monocytes, megakaryocytes, naive nCD4 T-cells, and neutrophils, respectively. The details on how the SIPs are shared across the different cell types were showed in **S2 Fig** and **S1 Dataset**. Similar to GTEx analyses of eQTLs’ tissue specificity [18,19], the most common configurations pertain to cell type-specific SIPs and shared SIPs (across all five cell types). In addition, principal component analysis (PCA) on the cumulative interaction scores reflects expected correlations between cell type-specific SIPs in each cell type, as well as between any SIP and those SIPs shared by all five cell types (**S3 Fig**).

Moreover, many cell type-specific SIPs correspond to known lineage-specific genes and have PIRs overlapping relevant GWAS variants (examples annotated by red dots in **Fig 1A–1E**) (see **Methods**, **S2 Dataset**). For example, the neutrophil SIP gene *DOCK8* is an immunodeficiency gene that is expressed in resting human neutrophils [20], and the macrophage SIP gene *FMNL2* is most highly expressed in macrophages and is cell type relevant [21–23]. The naive CD4 T-cell SIP gene *CD6* is a strong positive control, as this gene is essentially only expressed in CD4 T-cells [24]; *BACH2* plays a vital role in maintaining naive CD4 T-cells and regulating immune homeostasis [25]. All of these SIP genes have at least one PIR overlapping a GWAS identified SNP.

**Fig 2:**
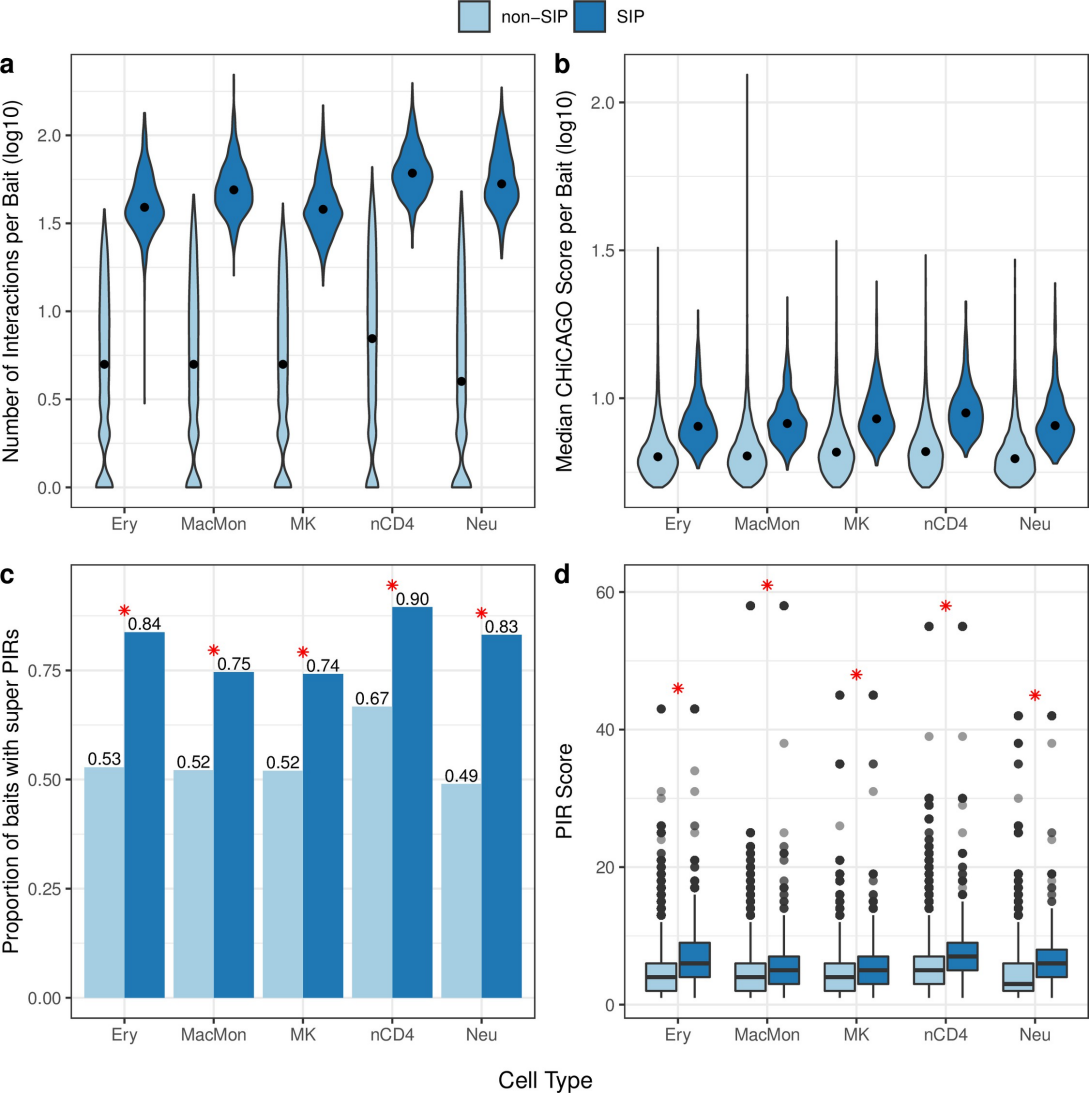
SIPs are driven by a large number of interactions. (**A**) Distribution of the number of significant interactions (log10 scale) between promoter bait and promoter interacting regions (PIRs) for SIPs and non-SIPs in each cell type. (**B**) Distribution of the median CHiCAGO score (log10 scale) of significant interactions per promoter bait for SIPs and non-SIPs in each cell type. The width of each violin corresponds to the frequency of interaction count (**A**) or median CHiCAGO score (**B**). The median of each distribution is marked by a black dot. (**C**) The proportion of SIP and non-SIP baits with super PIRs in each cell type. SIPs interact with a larger proportion of super PIRs than non-SIPs in each cell type (the red asterisk (*) denotes Chi-square *p*-value < 3.2e-35). (**D**) Distribution of PIR scores for SIPs and non-SIPs in each cell type. SIPs have significantly higher PIR scores than non-SIPs in each cell type (the red asterisk (*) denotes Wilcoxon *p*-value < 1.7e-50). Details for panels (**C**) and (**D**) can be found in S1 Table. (Ery = erythrocytes; MacMon = macrophages/monocytes; MK = megakaryocytes; nCD4 = naive CD4 T-cells; Neu = neutrophils).

The unusually high cumulative interaction scores at SIPs are driven by a large number of interactions, rather than a few interactions with large scores (**Fig 2A–2B**). SIP baits have a significantly greater number of other end interactions (i.e., PIRs) compared to non-SIP baits in each cell type (Wilcoxon *p-*value < 2.2e-16). The median number of significant interactions is 38–61 for SIPs and only 4–7 for non-SIPs (**Fig 2A**). SIPs interact with ~9 times more PIRs than non-SIPs on average (**Fig 2A**). However, the median CHiCAGO score [26] of significant interactions per bait, although statistically different, is comparable between SIPs and non-SIPs (the median is ~8.4 for SIPs and ~6.4 for non-SIPs, **Fig 2B**). We also note that median strength (as measured by the median CHiCAGO score [26]) of interactions for SIPs and non-SIPs are similar across cell types (**Fig 2B**). Of course, the SIP or non-SIP labels of each promoter changes from one cell type to another. Therefore, a specific promoter may well exhibit cell-type-specific effect of these interactions.

### SIPs and Super promoter-Interacting regulatory regions

In each cell type, ~59% of PIRs interact with a single promoter fragment while only ~10% of PIRs interact with 4 or more promoter fragments. Therefore, we define a super promoter-interacting region (super PIR) as a PIR interacting with at least 4 promoter fragments. As expected, SIPs interact with a larger proportion of super PIRs than non-SIPs in each cell type (Chi-square *p*-value < 3.2e-35) (**Fig 2C and S2 Table**). Approximately 74–90% of SIPs interact with a super PIR, whereas only 49–67% of non-SIPs interact with a super PIR. We assign each promoter region (bait) a PIR score, defined by its PIR with the maximum number of interactions. SIPs have significantly higher PIR scores than non-SIPs in each cell type (Wilcoxon *p*-value < 1.7e-50) (**Fig 2D and S3 Table**). The median PIR score is ~6 for SIPs and ~4 for non-SIPs (**Fig 2D and S3 Table**). These findings are not necessarily implicated by the definition of SIPs as in one extreme scenario, SIPs can be driven by a larger number of PIRs interacting just with the SIP and no other regions where the PIR score is 1 (lowest possible value). On the other hand, since we are taking the maximum across all PIRs and because SIPs have a much larger number of PIRs, the observation is not unexpected. The basic characteristics of SIPs (e.g., number of PIRs and proportion with super PIRs) are consistent across all five hematopoietic cell types.

### SIP PIRs Overlap with ATAC-seq Peaks and relevant GWAS Variants

**Fig 3:**
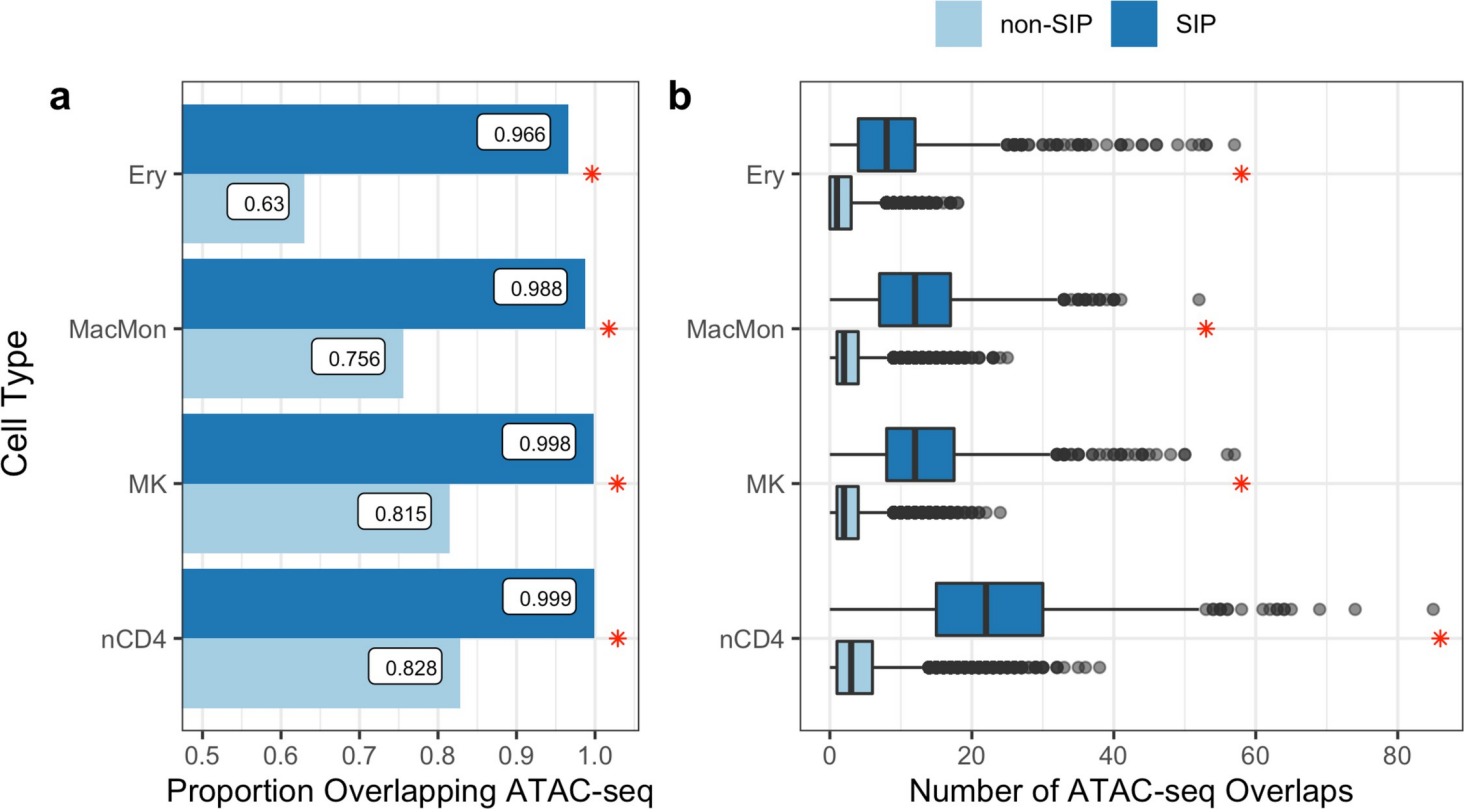
SIP PIRs overlap with ATAC-seq peaks. **(A)** In each cell type, the proportion of SIP having at least one PIR that overlaps a cell type-specific ATAC-seq peak is significantly greater than the proportion of non-SIP having at least one PIR that overlaps an ATAC-seq peak (the red asterisk (*) denotes Chi-square *p*-value < 2.2e-16). **(B)** The distribution of the number of PIRs overlapping with ATAC-seq peaks for each pcHi-C bait (y-axis) for each cell type (x-axis). In each cell type, significantly more SIP interactions overlap with ATAC-seq peaks compared to non-SIP interactions (the red asterisk (*) denotes two-sided t-test *p*-value < 1.2e-162). (Ery = erythrocytes; MacMon = macrophages/monocytes; MK = megakaryocytes; nCD4 = naive CD4 T-cells; Neu = neutrophils).

We can further characterize SIPs through their PIRs by examining the proximity of PIRs to open chromatin regions and known GWAS variants. In each cell type, at least 96% of SIPs have at least one PIR overlapping (at least one base pair) an ATAC-seq peak of the corresponding cell type [27], compared to 63–83% of non-SIPs. These proportional differences are statistically significant with Chi-square *p*-value < 2.9×10^−45^ (**Fig 3A**). We then compare the number of PIRs overlapping cell type-specific ATAC-seq peaks, for SIPs and non-SIPs (**Fig 3B**). In each cell type, significantly more SIP PIRs overlap with cell type-specific ATAC-seq peaks compared to non-SIP PIRs (*t*-test *p*-value < 1.2×10^−162^). The median number of ATAC-seq overlaps per bait is 8–22 for SIPs and only 1–3 for non-SIPs. Details on the number of overlaps as well as specific *p*-values are reported in **S3 Table**. Note that neutrophils are excluded from this analysis due to data availability (**S1 Note**). While informative, these findings are expected since SIPs have substantially more PIRs than non-SIPs. We additionally investigate individual PIRs of SIPs versus non-SIPs, finding that an individual PIR for a SIP exhibits slightly less overlap with ATAC-seq peaks than that for a non-SIP (**S23 Fig**). This is also expected due to the larger extent of speculated enhancer redundance for SIPs. In each cell type, the proportion of SIPs with a PIR that overlaps a cell type-specific ATAC-seq peak is significantly greater than the proportion of non-SIPs with a PIR that overlaps an ATAC-seq peak (Chi-square *p*-value < 2.9×10^−45^) (**Fig 3A**). We then compare the number of PIRs overlapping cell type-specific ATAC-seq peaks, for SIPs and non-SIPs (**Fig 3B**). In each cell type, significantly more SIP PIRs overlap with cell type-specific ATAC-seq peaks compared to non-SIP PIRs (*t*-test *p*-value < 1.2×10^−162^). The median number of ATAC-seq overlaps per bait is 8–22 for SIPs and only 1–3 for non-SIPs. Details on the number of overlaps as well as specific *p*-values are reported in **S3 Table**. Note that neutrophils are excluded from this analysis due to data availability (**S1 Note**).

**Fig 4:**
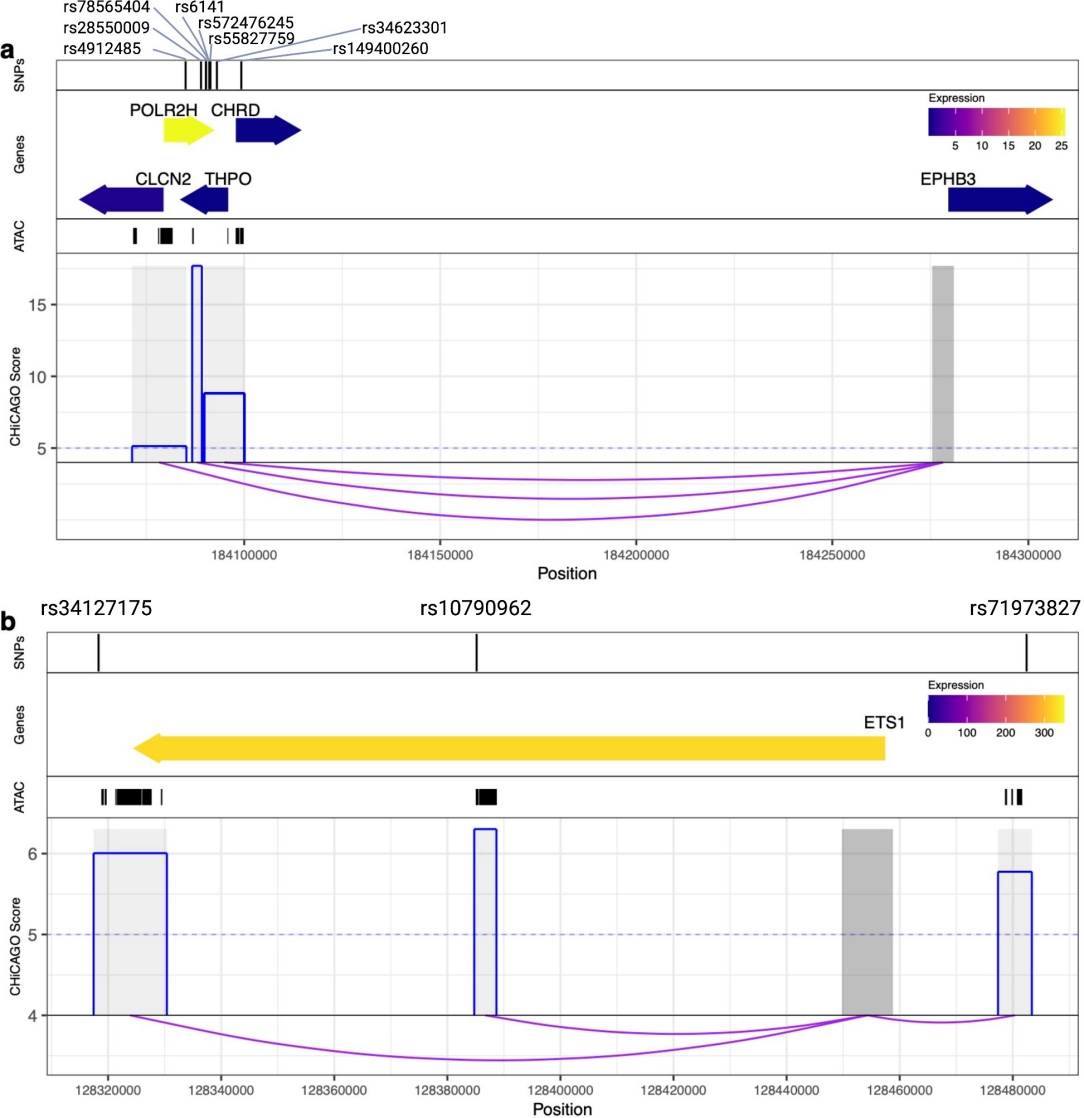
SIP subnetworks show the complex regulation of promoters for important hematopoietic cell type genes. First panel: position of SNPs. Second panel: position of SIP bait or PIR target gene(s), where the color corresponds to their exponentiated BLUEPRINT gene expression (equivalent to RPKM) in the respective cell type. Third panel: cell type-specific ATAC-seq peaks. Fourth panel: CHiCAGO scores (blue) of the interactions (depicted by purple arcs) between the SIP bait (dark grey) and the SIP PIRs (light grey). **(A)** Example of a megakaryocyte SIP subnetwork for platelet count related variants. **(B)** Example of a naive CD4 T-cell SIP subnetwork for lymphocyte count related variants.

Next, we examine the overlap between GWAS variants and PIRs. Blood cell lineage SIPs are more likely to have at least one PIR overlap with a relevant blood cell trait associated variant [28,29], compared to non-SIPs (see **Methods** and detailed in **S4A and S4B Fig**). In contrast, these blood linage SIPs show no or less significant enrichment for GWAS variants associated with schizophrenia (SCZ) [30] (**S4C and S4D Fig**). We found that cell-type-specific SIPs have insignificant less odds comparing to non-SIPs for SCZ GWAS variants, however all SIPs have significant greater odds comparing to non-SIPs for SCZ GWAS variants, and the odds were smaller and less significant than the enrichment results using the blood cell traits GWAS. That may be the reason that SIPs for each cell type include the lots of shared SIPs among all other cell types and/or brain related cell types, that may explain the reason of less enrichment if using all SIPs for each cell type for both GWAS studies (**S4B and S4D Fig**), but insignificant less odds if using the blood cell-type-specific SIPs. We additionally examined a wide range of transcription factor binding motifs including 374 motifs [29] which include blood lineage relevant motifs and negative control (negative control in the sense of not specifically relevant to blood lineages) motifs to annotate to the GWAS variants that overlap with SIP PIRs in **S2 Dataset**. For example, in **Fig 4A**, one SNP (3:184091102_T_G) residing in the MK SIP PIR disrupts transcription factor binding motifs of *ESRRA;ESRRB;NR5A1;NR6A1* (**S2 Dataset**). **S4 Table** summarizes the total number of GWAS variants residing SIP PIR regions and the number (%) of variants also disrupting transcription factor binding motifs. Overall, around 25.7% ~ 39.6% GWAS variants residing SIP PIR regions disrupt transcription factor binding motifs. Details SIP PIRs and their overlaps with relevant variants, ATAC-seq peaks and the transcription factor binding motifs can be found in **S2 Dataset**.

### SIP Subnetworks

By incorporating GWAS and open chromatin data with the pcHi-C data, we can determine SIP subnetworks that may provide insight into potential functional interactions. These SIP subnetworks are defined as having at least two PIRs that each overlap with a relevant statistically independent SNP and a cell type-specific ATAC-seq peak (see **Methods**). We identify 2–15 SIP subnetworks in each cell type/phenotype combination (see **Methods**, **S3 Dataset**). Details of the interactions and SNPs involved in these SIP subnetworks can be found in **S3 Dataset**.

We highlight two examples of SIP subnetworks in **Fig 4**. **Fig 4A** depicts the megakaryocyte SIP with bait located at the *EPHB3* gene interacting with three distinct regions that overlap with a total of 8 independent SNPs related to platelet count. These PIRs also overlap with megakaryocyte ATAC-seq peaks, and are near the key platelet related gene *THPO* or thrombopoietin, variants in which can lead to thrombocythemia (OMIM 600044 [31]). Thrombopoietin is essential for megakaryocyte proliferation and maturation, as well as for production of platelets. *EPHB3* encodes ephrin receptor B3, and plays roles in development, cell migration, and adhesion; variants in family member *EPHB2*, which also binds ephrin-B family ligands, are associated with a Mendelian bleeding disorder characterized by deficiencies in agonist-induced platelet aggregation and granule secretion (OMIM 600997 [31]). This SIP network suggests that *THPO* locus variants may also play a role in regulation of *EPHB3*. **Fig 4B** depicts the naive CD4 T-cell SIP with bait located at the *ETS1* gene interacting with three distinct PIRs that each overlap with an independent GWAS SNP related to lymphocyte count as well as a naive CD4 T-cell ATAC-seq peak. *ETS1* is a transcription factor highly expressed in CD4 T-cells known to regulate differentiation, survival and proliferation of lymphoid cells [32]; the *ETS1* locus is an important genetic regulator of risk for the autoimmune disorder systemic lupus erythematosus [33]. These SIP subnetworks show the complex regulation of promoters for important hematopoietic cell type genes, with multiple distinct genetic variants and regions of open chromatin acting together to regulate genes. Note that the visualizations of SIP subnetworks in **Fig 4** only depict specific PIRs, but each SIP interacts with many more PIRs (44 and 244, respectively).

### Partitioned heritability for Cell Type-Specific SIP PIRs using GWAS Summary statistics

We leveraged linkage disequilibrium score regression [34] (LDSC) using the cell type-specific SIP PIRs to partition the SNP heritability using European-ancestry GWAS summary statistics of 15 blood cell traits [35] (see **Methods**). Enrichment scores and corresponding *p*-values for each cell type and blood cell trait are displayed in **Figs S5 and 5**. PIRs of erythrocyte-specific SIPs are significantly enriched for red blood cell related traits including MCH, MCHC, MCV, RBC and RDW. Further, PIRs of megakaryocyte-specific SIPs are significantly enriched for PLT, PIRs of naive CD4 T-cell-specific SIPs are significantly enriched for LYM, and PIRs of neutrophil-specific SIPs are significantly enriched for NEU and WBC (**S5 Fig)**. Although *p*-values for neutrophil-specific SIP PIRs seem to stand out, we suggest interpreting the differences with care for two reasons. First, we note that the *p*-value range is rather tight: for example, smallest *p*-values for neutrophil-, megakaryocyte-, and erythrocyte-specific SIP PIRs are 3.9×10^−4^, 3.5×10^−3^ and 3.7×10^−3^ respectively, all within one order of magnitude difference. Second, the enrichment scores from PIRs of neutrophil-specific SIPs tend to be smaller than those from other cell types such as erythrocyte (**S5 Fig**). These results all show expected trait enrichments for each cell type. We also notice some less expected enrichments between PIRs of erythrocyte-specific SIPs and NEU, as well as between PIRs of neutrophil-specific SIPs and MCH, for example. While PIRs of macrophage/monocyte-specific SIPs are not enriched for white blood cell related traits (including monocyte counts), this may be due to the small number of macrophage/monocyte-specific SIPs (107) relative to the larger number of naive CD4 T-cell- and neutrophil-specific SIPs (283 and 274, respectively). When considering the PIRs of all SIPs, rather than only cell type specific SIP PIRs, PIRs of macrophage/monocyte SIPs are significantly enriched for MONO and WBC (**S6 Fig**).

**Fig 5:**
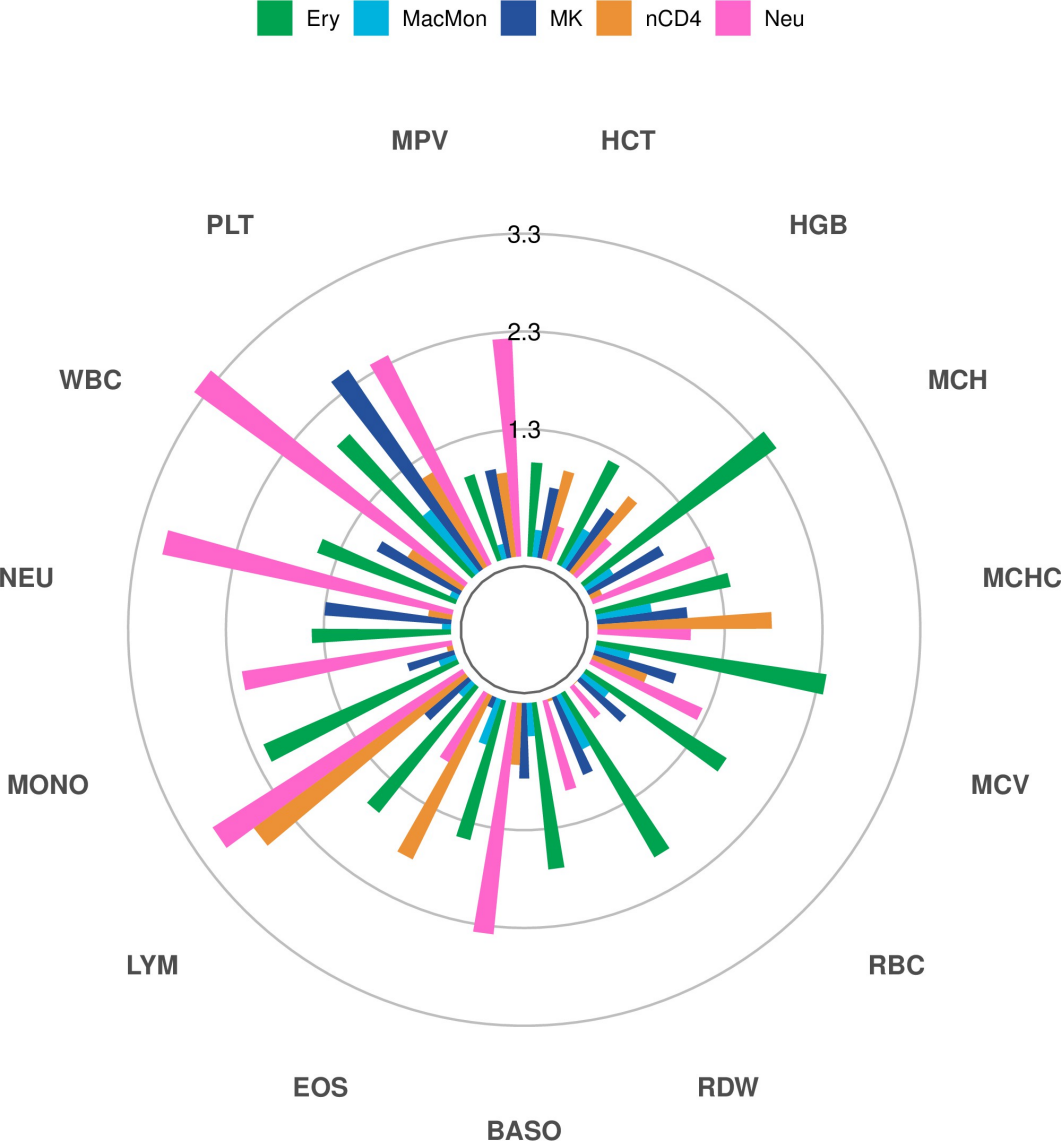
Partitioned heritability for blood cell traits shows enrichment between PIRs of cell type-specific SIPs and relevant traits. Enrichment score *p*-values (-log10 scale) for PIRs of cell type-specific SIPs and 15 blood cell traits. Bars passing the inner ring (1.3) correspond to statistically significant enrichment scores (*p*-value < 0.05). See **S5 Fig** for enrichment scores. (Cell types: Ery = erythrocytes; MacMon = macrophages/monocytes; MK = megakaryocytes; nCD4 = naive CD4 T-cells; Neu = neutrophils. Blood cell traits: HCT = Hematocrit; HGB = Hemoglobin; MCH = Mean Corpuscular Hemoglobin; MCHC = MCH Concentration; MCV = Mean Corpuscular Volume; RBC = Red Blood Cell Count; RDW = RBC Distribution Width; BASO = Basophil Count; EOS = Eosinophil Count; LYM = Lymphocyte Count; MONO = Monocyte Count; NEU = Neutrophil Count; WBC = White Blood Cell Count; PLT = Platelet Count; MPV = Mean Platelet Volume).

### SIPs Align with gene expression levels in a Cell Type-Specific manner

SIPs can also be characterized by their genes, and each SIP bait may correspond to more than one gene (**S1 Dataset and S1 Table**). Within each cell type, we ranked the genes according to their expression levels and calculated the fold enrichment of the genes classified as SIPs for higher gene expression (see **Methods**). All five cell types have well-expected trends in the relationship between SIP enrichment and gene expression (**S12A and S12B Fig**). For example, in erythrocytes there is 1.9-fold enrichment for a gene having a SIP in the highest quintile of gene expression (1st ranked) over the lowest (5th ranked) gene expression quintile (Chi-square *p*-value = 8.7×10^−14^). Conversely, for example, expression level for erythrocyte SIP genes is significantly higher than non-SIP genes in erythrocyte (*p*-value = 1.16×10^−56^, **S12B Fig**). In addition, among SIP genes, those with SIPs overlapping ATAC-seq peaks in the corresponding cell type exhibit higher expression levels than those not (**S12C Fig**).

We can further classify SIP genes as cell-type specific SIP genes, if the genes corresponding to cell type-specific SIP baits are not captured by any other promoter baits (some genes may be captured by multiple pcHi-C baits). In total, we annotate 251, 125, 385, 386, and 384 cell type-specific genes in erythrocytes, macrophages/monocytes, megakaryocytes, naive CD4 T-cells, and neutrophils, respectively (**S1 Table**). We also annotate 234 “shared” SIP genes (genes corresponding to SIPs shared across *all* five blood cell types evaluated).

We notice some trends in the gene expression of the 234 shared SIP genes that suggests that they have elevated expression levels in hematopoietic cell types [32] compared to the gene expression in other tissues (see **Methods**, **S7 Fig**). We find similar trends when comparing the gene expression of cell type-specific SIP genes to the expression in various other tissues (**S8 Fig**).

### CRISPRi Screen and SIPs in K562 cell line

Taking advantage of large scale CRISPRi perturbation screens in the human chronic myelogenous leukemia cell line K562, we additionally evaluated the impact of SIPs and PIRs for SIPs upon perturbation, compared to non-SIPs in K562. As baseline genotypes may affect enhancer activity, we first evaluated whether we observe any systematic difference in genotypes of genetic variants within SIP PIRs versus non-SIP PIRs. Specifically, we downloaded K562 genotypes (“ENCFF752OAX.vcf.gz”) from ENCODE [36], removed regions that are not diploid, and focused on genotypes of bi-allelic SNPs. As shown in **S24 Fig**, we find no evidence of differential genotype categories (0/0 for homozygous REF, 0/1 for heterozygotes, and 1/1 homozygous ALT) for SNPs residing in SIP PIRs versus non-SIP PIRs. Therefore, although still possible, there is no obvious reason to suspect that the genotypes in K562 cells affect enhancer activity in a manner that mask effects.

We then proceed to identify SIPs using K562 H3K27ac HiChIP data [37], finding 811 SIPs in K562, corresponding to 1,284 unique SIP genes (see **Methods**). Since H3K27ac is an enhancer mark, we also examined which promoters also serve as enhancers (i.e., which promoters are also PIRs for other promoters). We found that SIPs are significantly more likely to also act as an enhancer compared to non-SIPs (97.7% of SIPs vs 84.5% of non-SIPs; Chi-square *p*-value < 2.2×10^−16^).

We then assessed the effects of SIPs or PIRs of SIPs upon CRISPRi perturbation. Specifically, we used CRISPRi perturbation data from Gasperini et al 2019 [38], which perturbed 5,723 enhancer regions in K562 for impact on gene expression and identified 664 significant cis enhancer-gene pairs with adjusted *p*-value (using Benjamini-Hochberg FDR) of 10% [38]. We examined two aspects attempting to answer two questions. First, we studied whether perturbing PIRs of SIPs is more impactful than perturbing PIRs of non-SIPs. We hypothesize that disruption of SIP PIRs is likely less influential because of enhancer redundancy for SIPs. On the other hand, given the higher expression level of SIP corresponding genes, it tends to be more powerful to detect significance repression for SIP PIRs. Among the enhancer regions tested by Gasperini et al [38], 2.5% (16/652) of SIP PIRs significantly repressed the expression of the target gene, compared to 2.3% (61/2672) of non-SIP PIRs, with no significant difference (*p*-value = 0.908). We additionally compared effect sizes (absolute relative difference in median expression values) after perturbing PIRs of SIPs versus non-SIPs, which is less affected by differential power than statistical significance and would therefore reflect primarily the extent of enhancer redundancy. As expected, we observed smaller effect size when perturbing PIRs or SIPs than those of non-SIPs (mean effect size of 0.07 versus 0.08, Wilcoxon test *p*-value = 2.63×10^−8^) (**S25 Fig**).

Second, we examined whether perturbing SIPs (i.e., the promoter regions) is more impactful than perturbing non-SIPs. We hypothesize that disrupting SIPs is more impactful for two main reasons. First, for their cognate genes, despite enhancer redundancy, most if not all enhancers are expected to interact with the promoter regions. Second, SIPs are more likely to serve as potential enhancers that play regulatory role on other genes as we have shown above. SIPs involve in more promoter-promoter interactions and therefore likely have more potential to act as putative enhancers that play regulatory role compared to non-SIPs. CRISPRi data from Gasperini et al show that 13.8% (4/29) of SIPs significantly affect expression of target genes other than their cognate genes, compared to 1.3% (3/230) of non-SIPs (Fisher’s exact test *p*-value = 0.003). These results suggest that the SIPs are more likely to act as potential enhancers that play regulatory role compared to non-SIPs.

## Discussion

Hi-C has been widely adopted to study chromatin spatial organization. pcHi-C, a derivative of the Hi-C technology, enables the study of the promoter interactome, specifically. Importantly, recent studies have demonstrated the ability of pcHi-C analysis to link non-coding variants to their target genes.

By analyzing pcHi-C data, we catalogue super-interactive promoters (SIPs) in five blood cell types and present characteristics and analysis of SIPs in blood cell lineages. We in total identified 808–1,287 SIPs from major blood cell types, corresponding to 1,093–1,752 SIP genes, among which 125–386 are cell type specific. The characteristics of SIPs identified in blood cell lineages are consistent with those described of SIPs identified in brain cortex and human T cells [7,39], including enrichment for key blood lineage-specific genes, cell type specificity for most identified SIPs, and cell type-specific SIP enrichment in cells with higher expression of the regulated genes. We also demonstrate that SIPs share common properties across cell types, but align with cell type-specific genes. In our analyses, we find that SIPs’ regulatory networks are more likely to overlap with relevant GWAS variants and ATAC-seq peaks than non-SIP regulatory networks. We further find that cell type-specific SIP genes show enriched heritability in blood cell trait GWAS summary statistics. There results suggest that SIPs in relevant hematopoietic cell types can help identify GWAS variant target genes.

To shed insight on potential mechanisms distinguishing SIPs from non-SIPs, we performed analysis examining transcription factor binding motifs, distance to TAD boundaries, and gene density for SIPs versus non-SIPs. First, we collected 10 blood cell lineage relevant transcription factor binding motifs from [40–43] and performed motif enrichment analysis for SIPs versus non-SIPs, SIP PIRs versus non-SIP PIRs using fimo[44]. We find SIP PIRs tend to be enriched for binding motifs of a large number of transcription factors while SIPs themselves are less enriched (**S14 Fig**). Second, we examined the distance to the closest TAD boundary (from GM12878 [45]) for SIPs versus non-SIPs and SIP PIRs versus non-SIP PIRs. The results show that SIPs and SIP PIRs both tend to be slightly further away from TAD boundaries than non-SIPs and non-SIP PIRs (**S15 and S16 Figs**). These are expected because residing towards TAD centers allow more interactions on both sides. Finally, we compared overall gene density for SIPs versus non-SIPs and SIP PIRs versus non-SIP PIRs where the gene density is defined using Gencode v34. The results show that SIPs tend to reside in slightly less gene dense regions than non-SIPs, similarly for SIP PIRs compared to non-SIP PIRs (**S17 Fig**). These findings together suggest that SIPs and their PIRs tend to reside in slightly less gene dense regions, further away from TAD boundaries, thus enabling more within-TAD interactions and with its PIRs leveraging a number of transcription factors to achieve more precise control of the higher expression level of SIP genes.

We additionally identified SIPs using GM12878 Hi-C dataset [45], as well as K562 and GM12878 HiChIP datasets [46] to mitigate bait bias in pcHi-C data. Furthermore, for the HiChIP datasets, we evaluated the robustness of our SIP detection method to different replicates (2 replicates for GM12878 and 3 replicates for K562), bin resolution (5Kb and 10Kb), and sequencing depth. For pcHi-C data, we also evaluated the impact of maximum 1D genomic distance (attempted 1MB in addition to the 2MB default used so far). The results (**S18–S22 Figs**) showed consistent patterns that SIPs are driven by the large number of interactions and the SIPs interactions scores are slightly higher than non-SIPs.

Now that many blood cell lineage SIPs have been identified, a logical next step would be to disrupt SIPs or SIP PIRs and evaluate the effects on hematopoiesis. SIPs driven by few super strong interactions vs many significant (not necessarily all strong) interactions will have different implications for the design and prioritization of functional experiments. In our study, we find the SIPs are driven by large interactions with slightly higher CHiCAGO scores which means most SIPs are linked to multiple regulatory regions (as opposed to just having a few very strong interactions). These multiple regulatory regions are likely key for orchestrating fine transcriptional control of genes with SIPs. Multiple regulatory regions may also provide a level of “redundancy”, ensuring that even in the presence of an enhancer-disrupting genetic variant, appropriate transcriptional regulation can occur for important genes in a given hematopoietic cell type. Many key GWAS loci show allelic heterogeneity, with multiple rare and common variants (both coding and noncoding) impacting gene regulation (for example, at the *MPL* or *JAK2* locus for platelet traits [35,47,48]. Particularly for SIPs, genetic or epigenetic perturbations of one of these many putative regulatory regions (some of which may be tagged by statistically distinct GWAS SNPs) may be compensated for by other regulatory regions in the orchestra, leading to no apparent effect in vitro even when the perturbed region is functional in its native context. Researchers should consider this limitation when prioritizing loci and interpreting functional validation experiment results and may want to consider approaches that genetically or epigenetically edit multiple PIRs for a SIP simultaneously [49]. Cell type specificity of SIPs and their PIRs, together with complementary evidences from 1D epigenetic assay and eQTL/pQTL studies in relevant cell types, should also be considered in linking GWAS variants to genes and in the design of functional experiments [50]. We believe that the identification of SIPs can provide novel insights and evidence for gene regulation and facilitate the establishment of regulatory blueprint in a cell type and/or tissue-specific manner.

One limitation of the current study and of chromatin conformation studies in general is the lack of racial/ethnic diversity in contributing samples. Future studies are warranted to generate data from more diverse racial/ethnic background and examine chromatin folding patterns across these diverse groups accordingly.

The success of SIP characterization in neuronal, and now hematopoietic lineages, suggests the value of cataloguing SIPs in other cell types and incorporating those SIPs with results of GWAS analysis for relevant traits. It would also be interesting to examine condition-specific SIPs, such as different molecular environments triggered by drugs, toxic chemicals, diet, or stress, in various cell types. Doing so would allow for investigation on how gene expression varies in a cell-type specific manner under different environmental conditions. In addition, future work may involve exploring the relationship between super PIRs and super enhancers. Further experimental work to validate the cell type-specific SIP genes and the connection of these genes to corresponding blood cell traits will be required, but many attractive candidates have been identified through our systematic evaluation of promoters and their interacting regulatory regions in hematopoietic cell types.

## Methods

### Cell types

There are eight hematopoietic cell types in the pcHi-C data [13]: M0 macrophage, M1 macrophage, M2 macrophage, monocyte, neutrophil, erythrocyte, naive CD4 T-cell, and megakaryocyte. Since monocytes circulate in the blood and exist in tissues as macrophages in their mature form, we grouped the monocytes with the three macrophage types (by taking the average of the gene expression in BLUEPRINT [32] and the CHiCAGO [26] scores in pcHi-C data) to form one group. Thus, we focus on five cell types throughout this paper.

### Defining SIPs in blood cells

We first calculated the cumulative interaction scores for each promoter-containing anchor bin (bait) in the pcHi-C data [13], in each cell type. For each bait, the cumulative interaction score is the sum of the CHiCAGO scores of significant interactions (CHiCAGO score > = 5, as informed by Cairns et al. [26]). The CHiCAGO algorithm [26] robust detects DNA looping interactions for capture Hi-C data, explicitly modeling bait bias factors to increase accuracy. CHiCAGO scores quantify the significance of interaction with higher scores corresponding to more significant interactions. Conventionally, the score of 5 has been used as the threshold to define significant interactions. For each cell type, we calculated the SIP scores, or cumulative interaction scores for each bait, by summing over both bait-to-other-end and bait-to-bait interactions in pcHi-C data. We then ranked the SIP scores and identified the inflection point of the ranked baits. Following the ROSE algorithm to define super-enhancers [51,52], we defined inflection point or the SIP threshold point by finding the x-axis value at which the line tangent to the curve has a slope of 1. Following Song et al 2020, we loosely define SIPs as promoters with high cumulative chromatin interactions with other regions. More precisely, we define SIPs as promoters overlapping bait regions that exceed the inflection point, and other promoters as non-SIPs. SIPs are approximately the top 7.5% of cumulative interaction scores. We defined cell type-specific SIPs as promoter baits that only meet the SIP criteria (i.e. have extremely high cumulative interaction scores) in the corresponding cell type. Shared SIPs refer to promoter baits that are identified as SIPs in all five cell types.

For Hi-C and HiChIP, the SIP scores are defined as the summation of -log10 (fit-hi-c [53] q-value) and -log10 (MAPS [8] q-value) respectively, to replace CHiCAGO scores for pcHi-C data. Fit-hi-c and MAPs q-values quantify the statistical significance of chromatin interactions in Hi-C ad HiChIP data respectively (**S1 Text**).

### Define SIPs in K562

We first used the MAPS method [8] to call peaks (significant interactions defined as FDR < 0.01) on the K562 H3K27ac HiChIP data. Next, we defined promoter baits as the baits that overlapped with promoters defined in GENCODE v28. Accordingly, PIRs were defined as regions interacting with promoters with ChiCAGO score ≥ 5 from Javierre et al pcHi-C data [13]. We then used the -log10 (MAPS q-value) from the MAPS [8] calls as the interaction score for each promoter-PIR interaction, and took the cumulative sum of all interaction scores per promoter bait. By finding the inflection point of ranked promoter baits, we defined SIPs as those promoters with extremely high cumulative interactions scores (~ top 7%) (**S9–S11 Figs, S1 Text**). For assessing the effects of SIPs or PIRs of SIPs upon CRISPRi perturbation, we specifically used CRISPRi perturbation data from Gasperini et al 2019 [38] by examining the enhancer regions that overlapped at least 1bp with the SIP/non-SIP PIRs as well as the promoter bait regions themselves.

### SIP PIRs Overlap with relevant GWAS Variants

In each cell type, for every SIP, we determined if at least one PIR overlapped with a relevant blood cell trait variant (i.e., if a PIR region contained a variant), using summary statistics from the latest two GWAS studies on blood cell traits, including GWAS variants identified in European samples [48] as well as non-European and trans-ethnic analyses [35]. Phenotypes (i.e., relevant traits) considered for each cell type are as follows: any red blood cell trait (HCT, HGB, MCH, MCHC, RBC, RDW) for erythrocytes, MONO or WBC for macrophages/monocytes, PLT or MPV for megakaryocytes, LYM or WBC for naive CD4 T-cells, and NEU or WBC for neutrophils. Next, for each cell type, we randomly sampled non-SIPs (where *n* sampled is the number of SIPs in the respective cell type) and determined if at least one PIR overlapped with a relevant variant. Since the overlapping may be confounded by the length of bait, we used logistic regression to perform the enrichment analysis, adjusting bait length. Specifically, we fit the following model:

Bait‐PIR‐overlapping‐GWAS‐variants∼SIP‐status+bait‐length

where the binary outcome “Bait-PIR-overlapping-GWAS-variants” is defined by whether the bait has at least one PIR overlapping with GWAS variants, SIP-status is the binary predictor of interest (= 1 if the bait is a SIP and 0 otherwise), and bait-length is the length of bait that we want to adjust. We want to test whether SIP-status is associated with the outcome while adjusting bait length.

To construct SIP subnetworks, we only considered the statistically independent GWAS variants from Vuckovic et al. [48]. Consequently, each SIP subnetwork has PIRs that each overlap with a relevant statistically independent variant, as well as a cell type-specific ATAC-seq peak [27]. We identify SIP subnetworks for each of the following cell type/phenotype combinations: erythrocytes (HCT (2), HGB (2), MCH (7), MCHC (3), RBC (4), RDW (11)), macrophages/monocytes (MONO (5), WBC (1)), megakaryocytes (PLT (14), MPV (10)), and naive CD4 T-cells (LYM (15), WBC (2)). When removing the constraint of PIR overlapping with ATAC-seq data for neutrophil SIPs, as it is unavailable, we identify neutrophil SIP subnetworks for NEU (16) and WBC (22).

### Partitioned heritability for Cell Type-Specific SIP PIRs

We leveraged linkage disequilibrium score regression [34] (LDSC) using the PIRs of cell type-specific SIP to partition the SNP heritability for 15 blood cell traits from GWAS summary statistics from European ancestry individuals [35]. The LD scores were estimated from the European ancestry participants in the 1000 Genomes Project, and the common SNPs were defined using HapMap 3, both from the original Finucane et al 2015 paper and downloaded from https://alkesgroup.broadinstitute.org/LDSCORE/. LDSC jointly models 75 baselines annotations consisting of coding, UTR, promoter, and intron regions, histone marks, DNase I hypersensitive sites, ChromHMM/Segway predictions, regions that are conserved in mammals, super-enhancers, FANTOM5 enhancers, and LD-related annotations (recombination rate, nucleotide diversity CpG content, etc.) [54] that are not specific to any cell type.

### Comparing gene expression levels in Shared and Cell Type-Specific SIP Genes

We downloaded gene expression data for all tissues from the GTEx portal [55]. For comparison to our blood cell types of interest, we used gene expression from BLUEPRINT [32] for erythrocytes, macrophages/monocytes, megakaryocytes, naive CD4 T-cells, and neutrophils. Specifically, we measured gene expression level using exp(MMSEQ) with MMSEQ downloaded directly from BLUEPRINT. For each of the shared SIP genes, we computed the mean gene expression across all five blood cell types and the mean gene expression across all other tissues (non-blood cells). Next, we partitioned the shared SIP genes into percentiles based on the ranked mean gene expressions in blood cells (**S7A–S7B Fig**), and the ranked mean gene expressions in other tissues (**S7C–S7D Fig**). We followed a similar computational process for the cell type-specific SIP genes. For each set of cell type-specific SIP genes, we partitioned the genes into percentiles based on the ranked gene expression in the respective cell type (**S8A–S8B Fig**), and the ranked mean gene expressions in other tissues (**S8C–S8D Fig**).

### Fold enrichment test for highly expressed genes among genes with SIPs

Gene expression was ranked from highest (1st) to lowest (5th) quintile in each cell type. For each cell type, we calculated the proportion of SIP genes with rank r out of the total number of genes with rank r. Fold enrichment was then calculated relative to the group with the lowest gene expression (5th) and the significance level was obtained through a Chi-square test for proportions (for each cell type).

### Abbreviations of blood cell traits

HCT = Hematocrit; HGB = Hemoglobin; MCH = Mean Corpuscular Hemoglobin; MCHC = MCH Concentration; MCV = Mean Corpuscular Volume; RBC = Red Blood Cell Count; RDW = RBC Distribution Width; BASO = Basophil Count; EOS = Eosinophil Count; LYM = Lymphocyte Count; MONO = Monocyte Count; NEU = Neutrophil Count; WBC = White Blood Cell Count; PLT = Platelet Count; MPV = Mean Platelet Volume.

## Supporting information

S1 Text Supplemental Materials. (PDF)

S1 Note Neutrophils ATAC-seq peak was excluded. (PDF)

S1 Fig Cell type-specifically expressed genes exhibit higher levels of chromatin interactivity in the corresponding cell type. Each panel displays the empirical cumulative distribution function (CDF) of the number of significant pcHi-C interactions for shared versus cell type-specific genes in **(A)** erythrocytes, **(B)** macrophages/monocytes, **(C)** megakaryocytes, **(D)** naive CD4 T-cells, and **(E)** neutrophils. The average number of interactions for specific and shared genes within each cell type is reported along with the corresponding two-sided *t*-test *p*-value. **(F)** The number of specific genes per cell type.
(PDF)

S2 Fig A majority of SIPs are cell type-specific or shared across all five cell types. Details of SIPs are shared across cell types (black). Most SIPs, however, are cell-type specific (blue) or common between all five cell type groups (green). (Ery = erythrocytes; MacMon = macrophages/monocytes; MK = megakaryocytes; nCD4 = naive CD4 T-cells; Neu = neutrophils).
(PDF)

S3 Fig Principal component analysis (PCA) on cumulative interaction scores reflects expected correlations between SIPs. **(A)** PCA on the cumulative interaction scores of cell type-specific SIPs shows most correlation between erythrocyte- and megakaryocyte-specific SIPS, and distinction between those SIPs and the macrophage/monocyte-, naive CD4 T-cell- and neutrophil-specific SIPs (all immune function-related cell types), reflecting known relationships on the hematopoietic tree. **(B)** PCA on the cumulative interaction scores of SIPs, where “Shared” refers to SIPs shared across all five cell types, and “Other” refers to a SIP in at least one cell type.
(PDF)

S4 Fig Enrichment results of SIPs with having at least one PIR overlapping with GWAS variants. A. PIRs of blood lineage cell type-specific SIPs are more likely to overlap blood cell traits GWAS variants. SIPs are significantly more likely to have at least one PIR overlap with blood cell traits GWAS variants, compared to non-SIPs (odds ratio ≥ 1.72 and p-value ≤ 2.63×10^−3^). The only exception is MacMon where the point estimate of odds ratio (= 1.39) suggests the same direction of association but only not significant (p-value = 0.42). The insignificance may be due to the relatively small number of cell type-specific SIPs for MacMon. **B. PIRs of all SIPs for each blood lineage cell type are more likely to overlap blood cell traits GWAS variants.** In each cell type, all SIPs (regardless of specific or shared with other cell types) are more likely to have at least one PIR overlap with blood cell traits GWAS variants, compared to non-SIPs (odds ratio ≥ 2.34 and p-value ≤ 1.18×10^−6^). **C. PIRs of blood lineage cell type-specific SIPs show no enrichment of GWAS variants associated with schizophrenia (SCZ)**. PIRs for blood lineage cell type specific SIPs show no significant enrichment of SCZ GWAS variants (p-value ≥ 0.106). **D. PIRs of all SIPs for each blood lineage cell type are more likely to overlap SCZ GWAS variants**. We observe significant enrichment (p-value ≤ 6.30×10^−5^), but the magnitude of enrichment is less than that for blood cell traits (odds ratio ≥ 1.94). Odds ratio point estimates (purple dots) and corresponding 95% confidence intervals are shown. The text above the purple dot specifies the *p*-values.
(PDF)

S5 Fig Partitioned SNP heritability for blood cell traits—cell type-specific SIP PIRs. Enrichment scores for cell type-specific SIPs and 15 blood cell traits. (* denotes statistically significant enrichment score (*p* < 0.05); Ery = erythrocytes; MacMon = macrophages/monocytes; MK = megakaryocytes; nCD4 = naive CD4 T-cells; Neu = neutrophils).
(PDF)

S6 Fig Partitioned SNP heritability–for blood cell traits—all SIP PIRs. Enrichment scores for SIPs and 15 blood cell traits. (* denotes statistically significant enrichment score (*p* < 0.05); Ery = erythrocytes; MacMon = macrophages/monocytes; MK = megakaryocytes; nCD4 = naive CD4 T-cells; Neu = neutrophils).
(PDF)

S7 Fig Shared SIP genes have elevated expression levels in hematopoietic cell types. Violin plots showing the distribution of gene expression of shared SIP genes in various tissues as well as the five blood cell types. **(A)** Shared SIP genes with the top 10% of expression among blood cells. **(B)** Shared SIP genes with the top 10–20% of expression among blood cells. **(C)** Shared SIP genes with the top 10% of expression among other tissues (non-blood cells). **(D)** Shared SIP genes with the top 10–20% of expression among other tissues (non-blood cells). (Ery = erythrocytes; MacMon = macrophages/monocytes; MK = megakaryocytes; nCD4 = naive CD4 T-cells; Neu = neutrophils).
(PDF)

S8 Fig Neutrophil specific SIP genes have elevated expression levels in neutrophils. Violin plots showing the distribution of gene expression of neutrophil-specific SIP genes in various tissues as well as the five blood cell types. The cell type-specific SIP genes for erythrocytes, macrophages/monocytes, and naive CD4 T-cells show similar trends. **(A)** Neutrophil-specific SIP genes with the top 10% of neutrophils expression. **(B)** Neutrophil-specific SIP genes with the top 10–20% of neutrophils expression. **(C)** Neutrophil-specific SIP genes with the top 10% of expression among other tissues (non-blood cells). **(D)** Neutrophil-specific SIP genes with the top 10–20% of expression among other tissues (non-blood cells). (Ery = erythrocytes; MacMon = macrophages/monocyItes; MK = megakaryocytes; nCD4 = naive CD4 T-cells; Neu = neutrophils).
(PDF)

S9 Fig Hockey plots for each cell type show the ranked cumulative interaction MAP scores for K562 SIPs (baits). (PDF)

S10 Fig K562 SIPs are driven by a large number of interactions. Distribution of the number of significant interactions (log10 scale) between promoter bait and promoter interacting regions (PIRs) for SIPs and non-SIPs in each cell type. The median of each distribution is marked by a black dot.
(PDF)

S11 Fig The Distribution of the median MAP score (log10 scale) of significant interactions per promoter bait for SIPs and non-SIPs in K562. The median of each distribution is marked by a black dot.
(PDF)

S12 Fig **A. Highly expressed genes exhibit higher level of chromatin interactions in a cell type specific manner.** In each cell type, genes with the highest (1st) rank in expression show significantly higher chromatin interaction (as measured by SIP score) than those with the lowest (5th) ranked. The size of the circle denotes the fold-change (in SIP scores, quantifying the level of chromatin interaction) and the color denotes the Chi-square significance of enrichment (again for SIP scores). The Cochran-Armitage trend test p-value (one-sided) is also reported. **B. SIP genes detected in a cell type are more likely to be highly expressed in that cell type** (Wilcoxon test *p*-value = 2.95×10^−88^ ~ 7.14×10^−13^). **c. SIPs overlapping ATAC-seq peaks are more likely to be highly expressed than those not overlapping**. (Wilcoxon test *p*-value = 4.23×10^−40^~ 7.15×10^−16^) (Ery = erythrocytes; MacMon = macrophages/monocytes; MK = megakaryocytes; nCD4 = naive CD4 T-cells; Neu = neutrophils).
(PDF)

S13 Fig A. GM12878 SIPs are driven by a large number of interactions. Distribution of the number of significant interactions (log10 scale) between promoter bait and promoter interacting regions (PIRs) for SIPs and non-SIPs in GM12878 HiChIP. B. The Distribution of the median SIP score (-log10 MAPS q-value) of significant interactions per promoter bait for SIPs and non-SIPs in GM12878 HiChIP. C. Distribution of the number of significant interactions (log10 scale) between promoter bait and promoter interacting regions (PIRs) for SIPs and non-SIPs in GM12878 Hi-C. D. The Distribution of the median SIP score (-log10 q-value) of significant interactions per promoter bait for SIPs and non-SIPs in GM12878 Hi-C. The median of each distribution is marked by a black dot.
(PDF)

S14 Fig Transcription factor motif enrichment analysis for A. SIPs versus non-SIPs. B. SIP PIRs versus non-SIP PIRs. The Y-axis shows the transcription factor and X-axis shows odds ratio of enrichment. The dot denotes the point estimate of odds ratio and the line denotes the lower 95% confidence.
(PDF)

S15 Fig Distance to TAD boundary for SIPs versus non-SIPs. Distributions of distance to TAD boundaries for non-SIPs versus SIPs are visualized using side-by-side violin plots (left) and overlapping density plots (right).
(PDF)

S16 Fig Distance to TAD boundary for SIP PIRs versus non-SIP PIRs. Distributions of distance to TAD boundaries for non-SIP PIRs versus SIP PIRs are visualized using side-by-side violin plots (left) and overlapping density plots (right).
(PDF)

S17 Fig Gene density for SIPs versus non-SIPs and SIP PIRs versus non-SIP PIRs. **A. SIPs versus non-SIPs; B. SIP PIRs versus non-SIP PIRs.** The median of each distribution is marked by a black dot.
(PDF)

S18 Fig Venn diagrams show SIPs detected across biological replicates. **A.** Two replicates of GM12878 HiChIP data; and B. Three replicates of K562 HiChIP data.
(PDF)

S19 Fig SIP patterns at different resolution (GM12878 HiChIP data). **A**. The distribution of the number of significant interactions (log10 scale) for SIPs and non-SIPs, at 5kb resolution; **B**. The distribution of the number of significant interactions (log10 scale) for SIPs and non-SIPs, at 10kb resolution; **C**. The distribution of the median SIP score (-log10 MAPS q-value) of significant interactions for SIPs and non-SIPs, at 5kb resolution; **D.** The distribution of the median SIP score (-log10 MAPS q-value) of significant interactions for SIPs and non-SIPs, at 10kb resolution.
(PDF)

S20 Fig SIP patterns at different sequencing depths (GM12878 HiChIP data). **A**. The distribution of the number of significant interactions (log10 scale) for SIPs and non-SIPs, merging the two biological replicates; **B-C**. The distribution of the number of significant interactions (log10 scale) for SIPs and non-SIPs, for each biological replicate; **D**. The distribution of the median SIP score (-log10 MAPS q-value) of significant interactions for SIPs and non-SIPs, merging the two biological replicates; **E-F**. The distribution of the median SIP score (-log10 MAPS q-value) of significant interactions for SIPs and non-SIPs, for each biological replicate.
(PDF)

S21 Fig SIP patterns at different sequencing depths (K562 HiChIP data). **A**. The distribution of the number of significant interactions (log10 scale) for SIPs and non-SIPs, merging the three biological replicates; **B-D**. The distribution of the number of significant interactions (log10 scale) for SIPs and non-SIPs, for each biological replicate; **E**. The distribution of the median SIP score (-log10 MAPS q-value) of significant interactions for SIPs and non-SIPs, merging the three biological replicates; **F-H**. The distribution of the median SIP score (-log10 MAPS q-value) of significant interactions for SIPs and non-SIPs, for each biological replicate.
(PDF)

S22 Fig SIP patterns when detected using a maximum 1D genomic distance of 1Mb (Javierre et al pcHi-C data). **A**. The distribution of the number of significant interactions (log10 scale) for SIPs and non-SIPs**; B**. The distribution of the median CHiCAGO (log10 scale) of significant interactions per promoter bait for SIPs and non-SIPs.
(PDF)

S23 Fig Proportion of PIRs overlapping ATAC-seq peaks, SIPs versus non-SIPs. Dots denote median proportions, and triangles denote mean proportions.
(PDF)

S24 Fig The K562 genotypes comparison for SIP PIRs and non-SIP PIRs. “0/0” denotes homozygotes; “0/1” denotes heterozygotes; and “1/1” denotes alternative alleles homozygotes.
(PDF)

S25 Fig Effect size when perturbing PIRs, SIPs versus non-SIPs. Effect sizes are from CRISPRi experiments in Gasperini et al [1]. Dots denote median, and triangles denote mean.
(PDF)

S1 Table Total counts of cell type-specific SIPs and SIP genes as well as total counts of all SIPs and SIP genes, in each cell type. The total number of SIPs and SIP genes shared across all five cell types is also reported. Percent refers to the percent of total SIPs or SIP genes that are cell type-specific.
(PDF)

S2 Table SIPs interact with more super promoter-interacting regulatory regions (super PIRs) than non-SIPs. Details corresponding to **Fig 2**. For each cell type, the number of promoter baits interacting with a super PIR or typical PIR are reported, along with the corresponding ratio and Chi-square p-value. Median PIR scores for each cell type and promoter bait type are also reported, along with the corresponding Wilcoxon p-value for the difference in distribution between SIPs and non-SIPs. (Ery = erythrocytes; MacMon = macrophages/monocytes; MK = megakaryocytes; nCD4 = naive CD4 T-cells; Neu = neutrophils).
(PDF)

S3 Table SIP PIRs overlap with ATAC-seq peak regions. Details corresponding to **Fig 3**. For each cell type, the number of SIP PIRs and non-SIP PIRs that overlap with a cell type-specific ATAC-seq peak region are reported, along with the corresponding ratio and Chi-square p-value. The median (Med.) and average (Avg.) number of PIR interactions overlapping an ATAC-seq peak and corresponding t-test *p*-value for the difference between SIP and non-SIP PIRs is also reported. (Ovlp. = Overlap; Ery = erythrocyteIcMon = macrophages/monocytes; MK = megakaryocytes; nCD4 = naive CD4 T-cells; Neu = neutrophils).
(PDF)

S4 Table Around a third of the GWAS variants residing in the SIP PIR regions disrupt the transcript factor binding motifs. (PDF)

S5 Table Cell-type-specific CTCF Enrichment analysis for SIPs versus non-SIPs in K562 and GM12878. With applying fisher exact test, SIPs highly enriched for CTCF. K562 ORs = 2.46, *p*-value < 2.2×10^−16^ and GM12878 ORs = 2.92 and *p*-value < 2.2×10^−16^.
(PDF)

S1 Dataset Details of SIPs and SIP genes. Each sheet in the Excel file details the SIPs and SIP genes for each of the five cell types (Ery = erythrocytes; MacMon = macrophages/monocytes; MK = megakaryocytes; nCD4 = naive CD4 T-cells; Neu = neutrophils). The columns *baitID*, *baitChr*, *baitStart*, *baitEnd*, and *baitName* pertain to the promoter bait identifier, gene(s), and bait location (from the Javierre et al.^1^ pcHi-C data). The column *intscore* refers to the cumulative interaction score calculated to define SIPs, and the column *rank* reflects the order of SIP baits from largest score to smallest. Note that a SIP bait may have multiple genes and genes may correspond to multiple SIP baits. The column *specific* indicates if the SIP gene is only found in this particular cell type, and the column *shared* indicates if the SIP gene is shared across all five cell types. The next five columns, *SIP*.*Ery*, *SIP*.*MacMon*, *SIP*.*MK*, *SIP*.*nCD4*, and *SIP*.*Neu*, indicate whether that SIP is also found in the respective cell type. The column *ENSEMBL_ID* indicates the ENSEMBL ID (GRCh37), if found, for the gene names (*baitName)* provided in the pcHi-C data. The subsequent columns *Ery*, *MacMon*, *MK*, *nCD4*, and *Neu*, report the exponentiated BLUEPRINT gene expression (equivalent to RPKM) data in that cell type, if available.
(XLSX)

S2 Dataset Details of SIP PIR overlap with GWAS Variants. Each sheet in the Excel file provides information for the SIP PIRs that overlap with a relevant GWAS variant, for each of the five cell types (Ery = erythrocytes; MacMon = macrophages/monocytes; MK = megakaryocytes; nCD4 = naive CD4 T-cells; Neu = neutrophils). The columns *baitID*, *baitChr*, *baitStart*, *baitEnd*, and *baitName* pertain to the promoter bait identifier, gene(s), and bait location (from the Javierre et al. [1] pcHi-C data). The columns *oeStart*, *oeEnd*, *oeID*, and *oeName* pertain to the other end (PIR) location, identifier, and gene(s). The column *specific* indicates if the SIP gene is only found in this particular cell type (i.e., a cell type-specific SIP). The remaining columns pertain to the GWAS variants: *Phenotype*, *VariantID*, *rsID*, *Position* (hg19 base pair location of variant), *pval*, *ancestry*, and *data* (Vuckovic et al. [2] or Chen et al. [3]). The column “ATACseq_olap” denote whether the variant overlaps with cell-type-specific ATAC-seq peaks and the column “Motifs_genename” annotates transcription factor binding motifs the variant disrupts.
(XLSX)

S3 Dataset Details of SIP Subnetworks. Each sheet in the Excel file details the SIP subnetworks for a cell type and a particular phenotype. The naming convention for each sheet is “CellType_Phenotype”. A table of abbreviations and total SIP subnetworks for the cell type/phenotype combinations can be found on the first sheet, “README”, along with column descriptions.
(XLSX)
