# Supplementary material for: Super interactive promoters provide insight into cell type-specific regulatory networks in blood lineage cell types
Source: PLoS Genet. 2022 Jan 31;18(1):e1009984. doi: 10.1371/journal.pgen.1009984 (PMC8830683; doi:10.1371/journal.pgen.1009984)
Supplement: S1 Text — (PDF) [file pgen.1009984.s001.pdf]

Supplemental Materials:

Cell Type-Specifically Expressed Genes Exhibit Higher Levels of Chromatin Interactivity in the Corresponding Cell Type than Shared Genes

We first explored the relationship between chromatin interactivity and gene expression in a cell type-specific manner. We examined this relationship using pcHi-C data from Javierre et al. [1] and gene expression data from BLUEPRINT [2], in each of the five hematopoietic cell types: erythrocyte (Ery), macrophage/monocyte (MacMon), megakaryocyte (MK), naive CD4 T-cell (nCD4), and neutrophil (Neu) (Methods). We classified genes as "specific" (expressed in a cell type-specific manner) or "shared" across the five cell types. The promoters for cell type-specific genes have significantly more interactions than the shared genes across all five cell types (p-value < 0.05) (**S1A-E Fig**). Similar results were observed by Song et al. in neuron cells [3].

We classified genes as cell type-specific or shared via the Shannon entropy across the five cell types. Gene expression data was downloaded from BLUEPRINT [2]. Since this gene expression is calculated by MMSEQ, we took exponentials so that transcript quantification was comparable to RPKM. For each gene, we first filtered out the lowly expressed genes with gene expression < 1 in all cell types and then calculated the normalized gene expression as the gene expression in one cell type divided by the sum of the gene's expression across all five cell types. Next, we calculated the entropy (defined as the distance to log2(K), where K=5 is the number of cell types) using the relative gene expression across cell types. We defined cell type-specific genes as those with entropy > 0.5, in the respective cell type, and shared genes across cell types as those with entropy < 0.1. Approximately 534-1,814 genes are cell type-specific, depending on cell type (**S1F Fig**), and 1,476 genes meet the shared gene criteria.

Use Hi-C and HiChIP data to guard against potential bias of promoter-capture Hi-C

Promoter capture Hi-C (pcHi-C) has potential bait bias due to its design. Although the CHiCAGO algorithm corrects potential differential bait capture efficiency by incorporating bait bias factors into their model, the difference across baits in pcHi-C data cannot be ignored or completely corrected by

mathematical modeling. To guard against the potential bait bias in pcHi-C data, we performed further evaluations using Hi-C and HiChIP data available for blood cell lineages, namely Hi-C data for GM12878 [4], as well as H3K27ac HiChIP data for K562 and GM12878 [5]. In SIP score definition, we use -log10(Fit-Hi-C [6] q-value) and -log10(MAPS [7] q-value), respectively for Hi-C and HiChIP data, to replace CHiCAGO score for pcHi-C data. Overall, the conclusions, our conclusions, specifically that SIPs are primarily driven by a large number of interactions while interaction strength (as measured by SIP score) for SIPs is only slightly higher than that for non-SIPs, still hold, using these Hi-C and HiChIP data (**S10, S11 and S13 Figs**).

Reference

1. Javierre BM, Burren OS, Wilder SP, Kreuzhuber R, Hill SM, Sewitz S, et al. Lineage-Specific Genome Architecture Links Enhancers and Non-coding Disease Variants to Target Gene Promoters. Cell. 2016;167(5):1369-84 e19. Epub 2016/11/20. doi: 10.1016/j.cell.2016.09.037. PubMed PMID: 27863249; PubMed Central PMCID: PMCPMC5123897.
2. Chen L, Kostadima M, Martens JHA, Canu G, Garcia SP, Turro E, et al. Transcriptional diversity during lineage commitment of human blood progenitors. Science (New York, NY). 2014;345(6204):1251033. Epub 2014/09/27. doi: 10.1126/science.1251033. PubMed PMID: 25258084; PubMed Central PMCID: PMCPMC4254742.
3. Song M, Pebworth M-P, Yang X, Abnousi A, Fan C, Wen J, et al. Cell-type-specific 3D epigenomes in the developing human cortex. Nature. 2020;587(7835):644-9. Epub 2020/10/14. doi: 10.1038/s41586-020-2825-4. PubMed PMID: 33057195.
4. Schmitt AD, Hu M, Jung I, Xu Z, Qiu Y, Tan CL, et al. A Compendium of Chromatin Contact Maps Reveals Spatially Active Regions in the Human Genome. Cell reports. 2016;17(8):2042-59. Epub 2016/11/17. doi: 10.1016/j.celrep.2016.10.061. PubMed PMID: 27851967.
5. Mumbach MR, Satpathy AT, Boyle EA, Dai C, Gowen BG, Cho SW, et al. Enhancer connectome in primary human cells identifies target genes of disease-associated DNA elements. Nature genetics. 2017;49(11):1602-12. Epub 2017/09/26. doi: 10.1038/ng.3963. PubMed PMID: 28945252; PubMed Central PMCID: PMCPMC5805393.
6. Ay F, Bailey TL, Noble WS. Statistical confidence estimation for Hi-C data reveals regulatory chromatin contacts. Genome Res. 2014. Epub 2014/02/07. doi: 10.1101/gr.160374.113. PubMed PMID: 24501021.
7. Juric I, Yu M, Abnousi A, Raviram R, Fang R, Zhao Y, et al. MAPS: Model-based analysis of long-range chromatin interactions from PLAC-seq and HiChIP experiments. PLoS Comput Biol. 2019;15(4):e1006982. Epub 2019/04/16. doi: 10.1371/journal.pcbi.1006982. PubMed PMID: 30986246; PubMed Central PMCID: PMCPMC6483256.
