# Supplementary material for: Super interactive promoters provide insight into cell type-specific regulatory networks in blood lineage cell types
Source: PLoS Genet. 2022 Jan 31;18(1):e1009984. doi: 10.1371/journal.pgen.1009984 (PMC8830683; doi:10.1371/journal.pgen.1009984)
Supplement: S1 Note — (PDF) [file pgen.1009984.s002.pdf]

S1 Note. Neutrophils ATAC-seq peak was excluded.

Neutrophils were excluded from the SIP and ATAC-seq peak analysis. The Ulirsch et al. [1] ATAC-seq data used in this analysis does not include neutrophil ATAC-seq. Chen et al.[2] isolated neutrophils from two healthy donors' blood. Peak calling of this data (performed by MACS2 narrow peak mode with default parameters –q 0.01 –nomodel –shift 0) resulted in ~2,000 neutrophil ATAC-seq peaks, which is 1-2 orders of magnitude smaller than expected based on ATAC-seq in other hematopoietic cell types and consistent with the findings of Chen et. al who note that neutrophils have fewer chromatin accessibility peaks than do cell types with comparable sequencing depths and alignment rates. Since neutrophils are terminally differentiated cells with a short lifespan and the accessible chromatin peaks are not associated with usual euchromatin marks, it is possible that ATAC-seq peaks are not enriched or relevant for neutrophil traits. We also note that ATAC-seq data from monocytes was used for analyses involving the macrophage/monocyte SIPs.

Reference

1. Ulirsch JC, Lareau CA, Bao EL, Ludwig LS, Guo MH, Benner C, et al. Interrogation of human hematopoiesis at single-cell and single-variant resolution. Nat Genet. 2019;51(4):683-93. Epub 2019/03/13. doi: 10.1038/s41588-019-0362-6. PubMed PMID: 30858613; PubMed Central PMCID: PMCPMC6441389.
2. Chen X, Shen Y, Draper W, Buenrostro JD, Litzenburger U, Cho SW, et al. ATAC-see reveals the accessible genome by transposase-mediated imaging and sequencing. Nature methods. 2016;13(12):1013-20. doi: 10.1038/nmeth.4031.
