# Supplementary material for: Super interactive promoters provide insight into cell type-specific regulatory networks in blood lineage cell types
Source: PLoS Genet. 2022 Jan 31;18(1):e1009984. doi: 10.1371/journal.pgen.1009984 (PMC8830683; doi:10.1371/journal.pgen.1009984)
Supplement: S1 Fig — Each panel displays the empirical cumulative distribution function (CDF) of the number of significant pcHi-C interactions for shared versus cell type-specific genes in (A) erythrocytes, (B) macrophages/monocytes, (C) megakaryocytes, (D) naive CD4 T-cells, and (E) neutrophils. The average number of interactions for specific and shared genes within each cell type is reported along with the corresponding two-sided t-test p-value. (F) The number of specific genes per cell type. (PDF) [file pgen.1009984.s003.pdf]

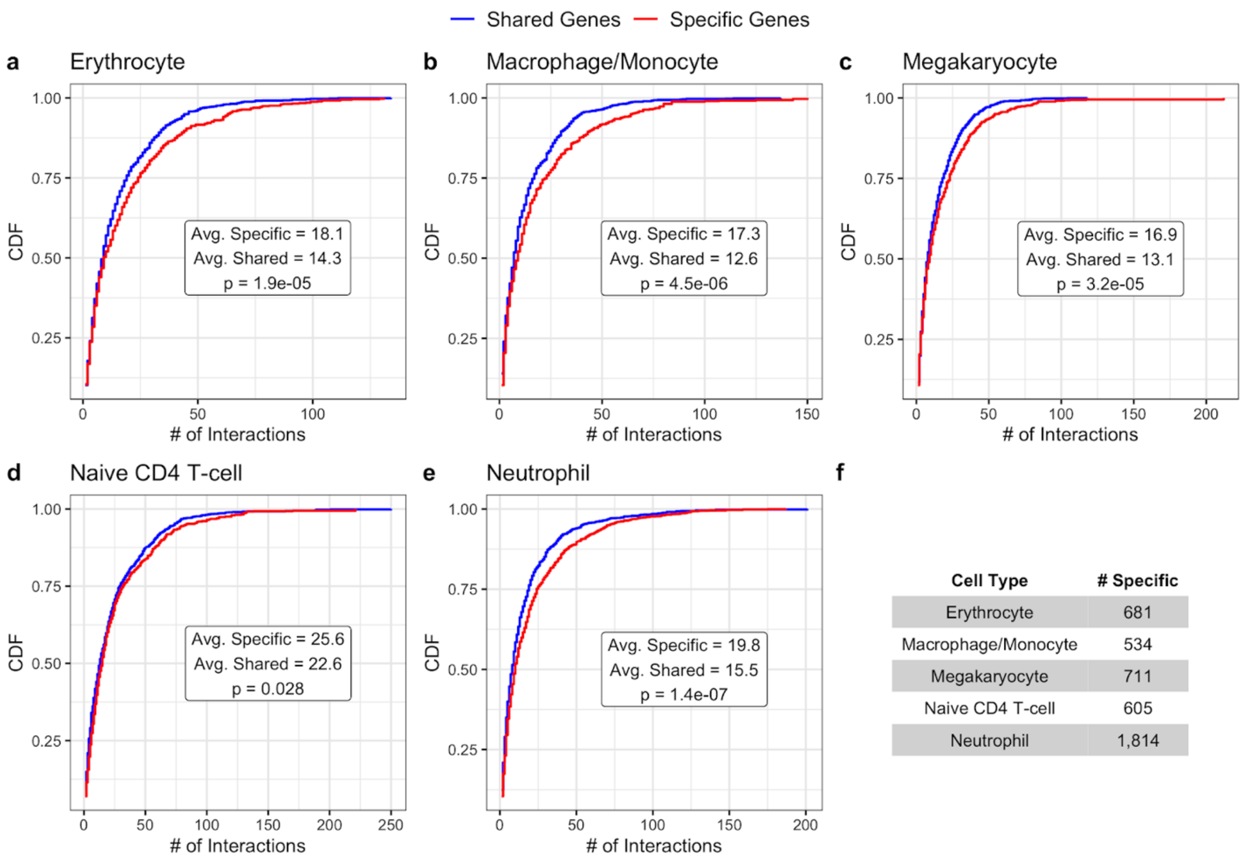

Cell Type	# Specific
Erythrocyte	681
Macrophage/Monocyte	534
Megakaryocyte	711
Naive CD4 T-cell	605
Neutrophil	1,814

S1 Fig. Cell type-specifically expressed genes exhibit higher levels of chromatin interactivity in the corresponding cell type. Each panel displays the empirical cumulative distribution function (CDF) of the number of significant pcHi-C interactions for shared versus cell type-specific genes in **(A)** erythrocytes, **(B)** macrophages/monocytes, **(C)** megakaryocytes, **(D)** naive CD4 T-cells, and **(E)** neutrophils. The average number of interactions for specific and shared genes within each cell type is reported along with the corresponding two-sided *t*-test *p*-value. **(F)** The number of specific genes per cell type.
