# Supplementary material for: Super interactive promoters provide insight into cell type-specific regulatory networks in blood lineage cell types
Source: PLoS Genet. 2022 Jan 31;18(1):e1009984. doi: 10.1371/journal.pgen.1009984 (PMC8830683; doi:10.1371/journal.pgen.1009984)
Supplement: S2 Fig — Details of SIPs are shared across cell types (black). Most SIPs, however, are cell-type specific (blue) or common between all five cell type groups (green). (Ery = erythrocytes; MacMon = macrophages/monocytes; MK = megakaryocytes; nCD4 = naive CD4 T-cells; Neu = neutrophils). (PDF) [file pgen.1009984.s004.pdf]

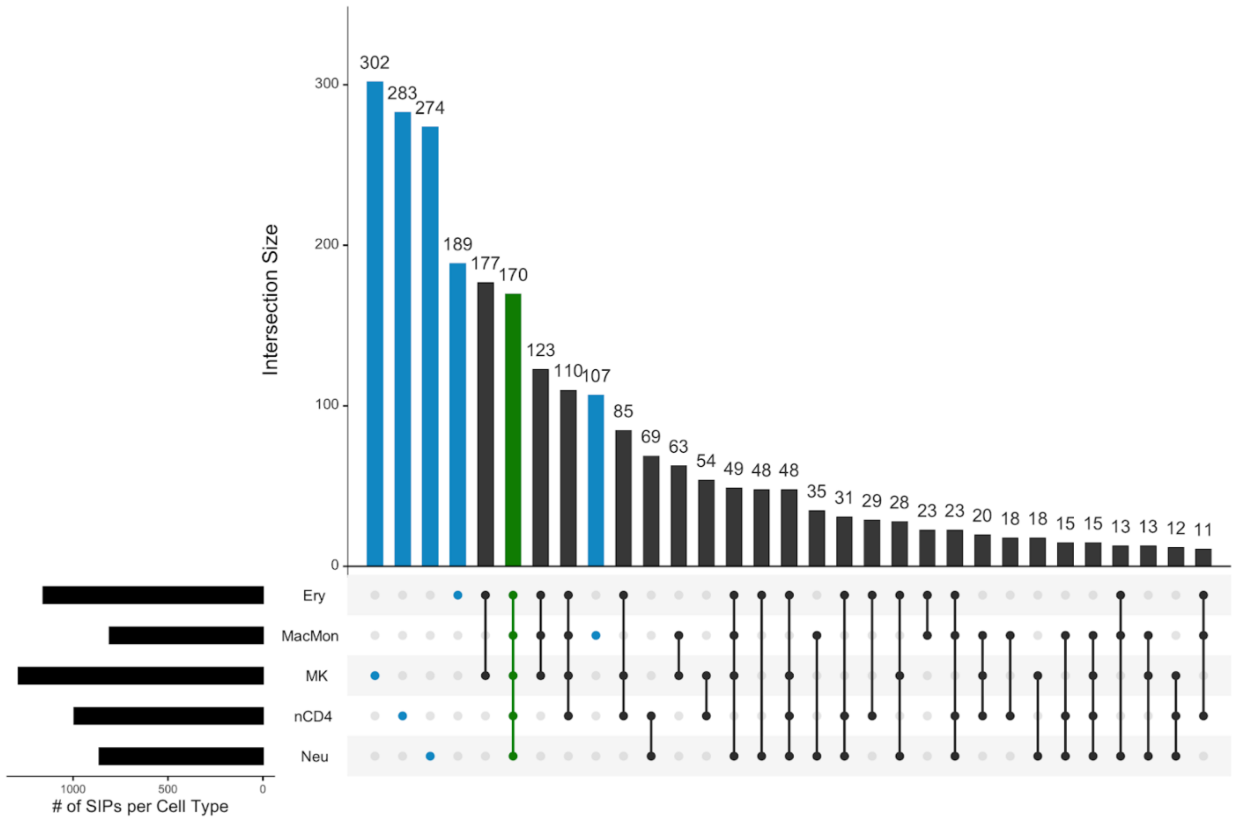

S2 Fig. A majority of SIPs are cell type-specific or shared across all five cell types. Details of SIPs are shared across cell types (black). Most SIPs, however, are cell-type specific (blue) or common between all five cell type groups (green). (Ery = erythrocytes; MacMon = macrophages/monocytes; MK = megakaryocytes; nCD4 = naive CD4 T-cells; Neu = neutrophils)
