# Supplementary material for: Super interactive promoters provide insight into cell type-specific regulatory networks in blood lineage cell types
Source: PLoS Genet. 2022 Jan 31;18(1):e1009984. doi: 10.1371/journal.pgen.1009984 (PMC8830683; doi:10.1371/journal.pgen.1009984)
Supplement: S3 Fig — (A) PCA on the cumulative interaction scores of cell type-specific SIPs shows most correlation between erythrocyte- and megakaryocyte-specific SIPS, and distinction between those SIPs and the macrophage/monocyte-, naive CD4 T-cell- and neutrophil-specific SIPs (all immune function-related cell types), reflecting known relationships on the hematopoietic tree. (B) PCA on the cumulative interaction scores of SIPs, where “Shared” refers to SIPs shared across all five cell types, and “Other” refers to a SIP in at least one cell type. (PDF) [file pgen.1009984.s005.pdf]

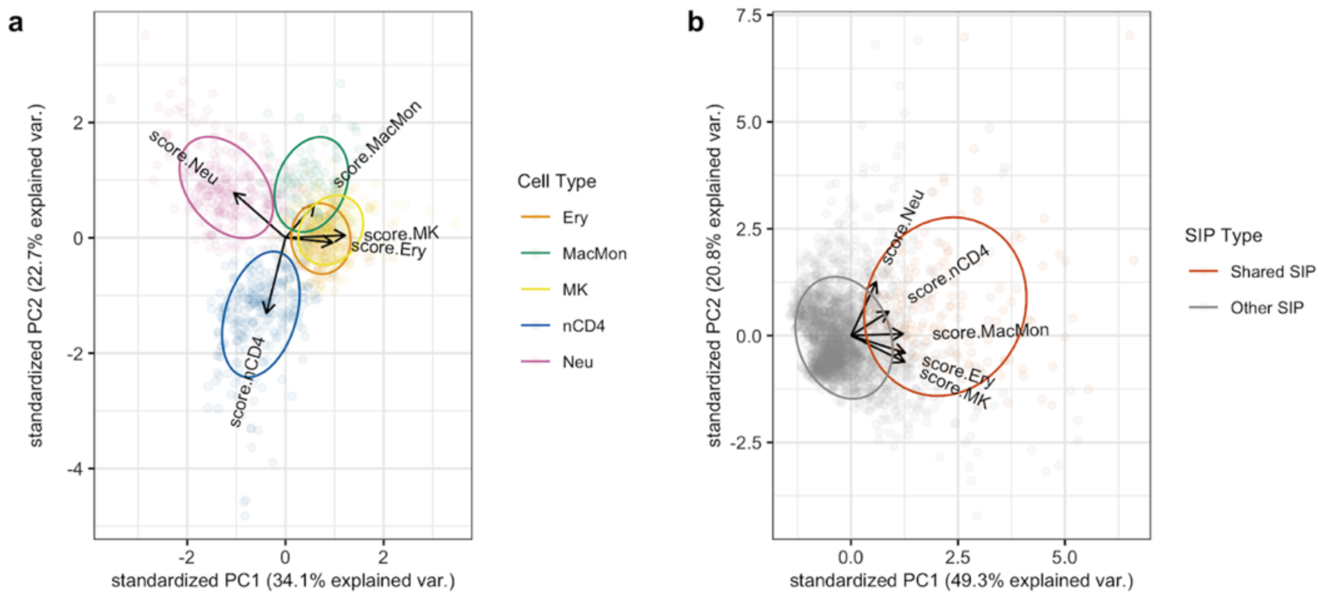

S3 Fig. Principal component analysis (PCA) on cumulative interaction scores reflects expected correlations between SIPs. (A) PCA on the cumulative interaction scores of cell type-specific SIPs shows most correlation between erythrocyte- and megakaryocyte-specific SIPS, and distinction between those SIPs and the macrophage/monocyte-, naive CD4 T-cell- and neutrophil-specific SIPs (all immune function-related cell types), reflecting known relationships on the hematopoietic tree. **(B)** PCA on the cumulative interaction scores of SIPs, where "Shared" refers to SIPs shared across all five cell types, and "Other" refers to a SIP in at least one cell type.
