# Supplementary material for: Super interactive promoters provide insight into cell type-specific regulatory networks in blood lineage cell types
Source: PLoS Genet. 2022 Jan 31;18(1):e1009984. doi: 10.1371/journal.pgen.1009984 (PMC8830683; doi:10.1371/journal.pgen.1009984)
Supplement: S4 Fig — SIPs are significantly more likely to have at least one PIR overlap with blood cell traits GWAS variants, compared to non-SIPs (odds ratio ≥ 1.72 and p-value ≤ 2.63×10^−3^). The only exception is MacMon where the point estimate of odds ratio (= 1.39) suggests the same direction of association but only not significant (p-value = 0.42). The insignificance may be due to the relatively small number of cell type-specific SIPs for MacMon. B. PIRs of all SIPs for each blood lineage cell type are more likely to overlap blood cell traits GWAS variants. In each cell type, all SIPs (regardless of specific or shared with other cell types) are more likely to have at least one PIR overlap with blood cell traits GWAS variants, compared to non-SIPs (odds ratio ≥ 2.34 and p-value ≤ 1.18×10^−6^). C. PIRs of blood lineage cell type-specific SIPs show no enrichment of GWAS variants associated with schizophrenia (SCZ). PIRs for blood lineage cell type specific SIPs show no significant enrichment of SCZ GWAS variants (p-value ≥ 0.106). D. PIRs of all SIPs for each blood lineage cell type are more likely to overlap SCZ GWAS variants. We observe significant enrichment (p-value ≤ 6.30×10^−5^), but the magnitude of enrichment is less than that for blood cell traits (odds ratio ≥ 1.94). Odds ratio point estimates (purple dots) and corresponding 95% confidence intervals are shown. The text above the purple dot specifies the p-values. (PDF) [file pgen.1009984.s006.pdf]

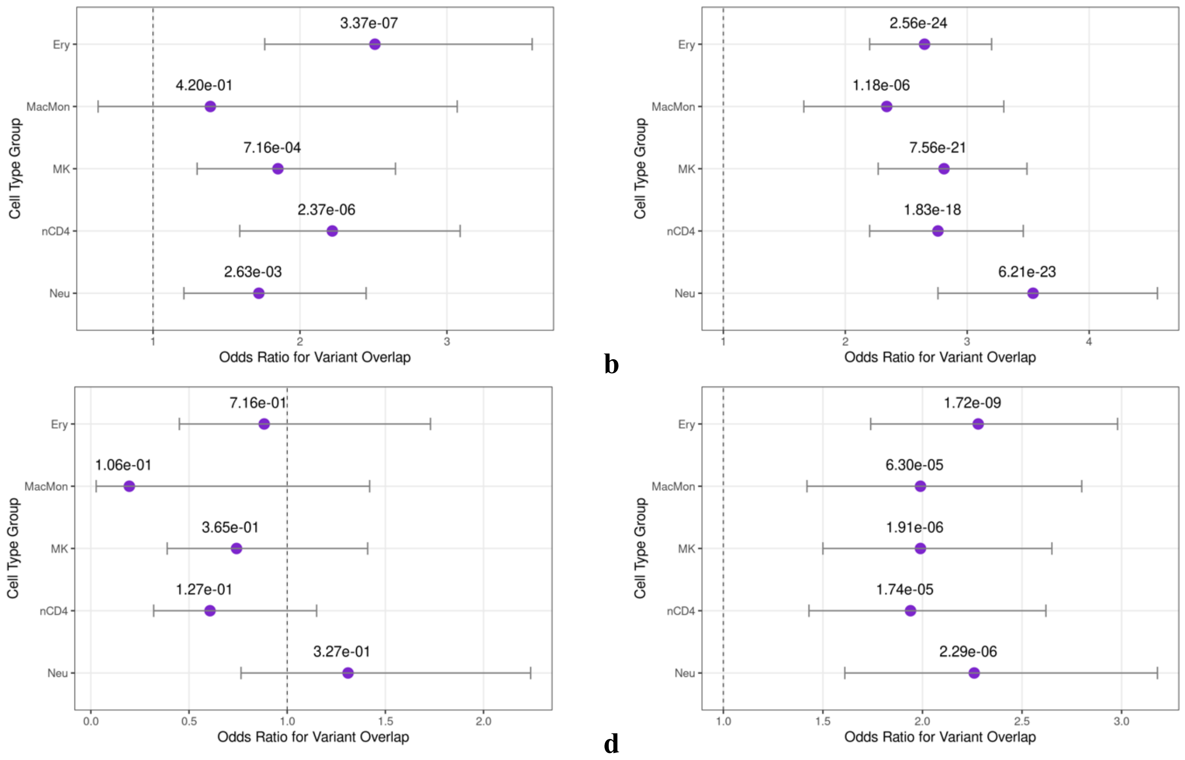

S4 Fig. Enrichment results of SIPs with having at least one PIR overlapping with GWAS variants. (A) PIRs of blood lineage cell type-specific SIPs are more likely to overlap blood cell traits GWAS variants. SIPs are significantly more likely to have at least one PIR overlap with blood cell traits GWAS variants, compared to non-SIPs (odds ratio ≥ 1.72 and p-value $\leqslant 2.63\times10^{-3}$). The only exception is MacMon where the point estimate of odds ratio (= 1.39) suggests the same direction of association but only not significant (p-value = 0.42). The insignificance may be due to the relatively small number of cell type-specific SIPs for MacMon. **(B) PIRs of all SIPs for each blood lineage cell type are more likely to overlap blood cell traits GWAS variants.** In each cell type, all SIPs (regardless of specific or shared with other cell types) are more likely to have at least one PIR overlap with blood cell traits GWAS variants, compared to non-SIPs (odds ratio $\geqslant 2.34$ and p-value $\leqslant 1.18\times10^{-6}$). **(C) PIRs of blood lineage cell type-specific SIPs show no enrichment of GWAS variants associated with schizophrenia (SCZ)**. PIRs for blood lineage cell type specific SIPs show no significant enrichment of SCZ GWAS variants (p-value $\geqslant 0.106$). **(D) PIRs of all SIPs for each blood lineage cell type are more likely to overlap SCZ GWAS variants**. We observe significant enrichment (p-value $\leq 6.30\times10^{-5}$), but the magnitude of

enrichment is less than that for blood cell traits (odds ratio ≥ 1.94). Odds ratio point estimates (purple dots) and corresponding 95% confidence intervals are shown. The text above the purple dot specifies the *p*-values.
