# Supplementary material for: Super interactive promoters provide insight into cell type-specific regulatory networks in blood lineage cell types
Source: PLoS Genet. 2022 Jan 31;18(1):e1009984. doi: 10.1371/journal.pgen.1009984 (PMC8830683; doi:10.1371/journal.pgen.1009984)
Supplement: S6 Fig — Enrichment scores for SIPs and 15 blood cell traits. (* denotes statistically significant enrichment score (p < 0.05); Ery = erythrocytes; MacMon = macrophages/monocytes; MK = megakaryocytes; nCD4 = naive CD4 T-cells; Neu = neutrophils). (PDF) [file pgen.1009984.s008.pdf]

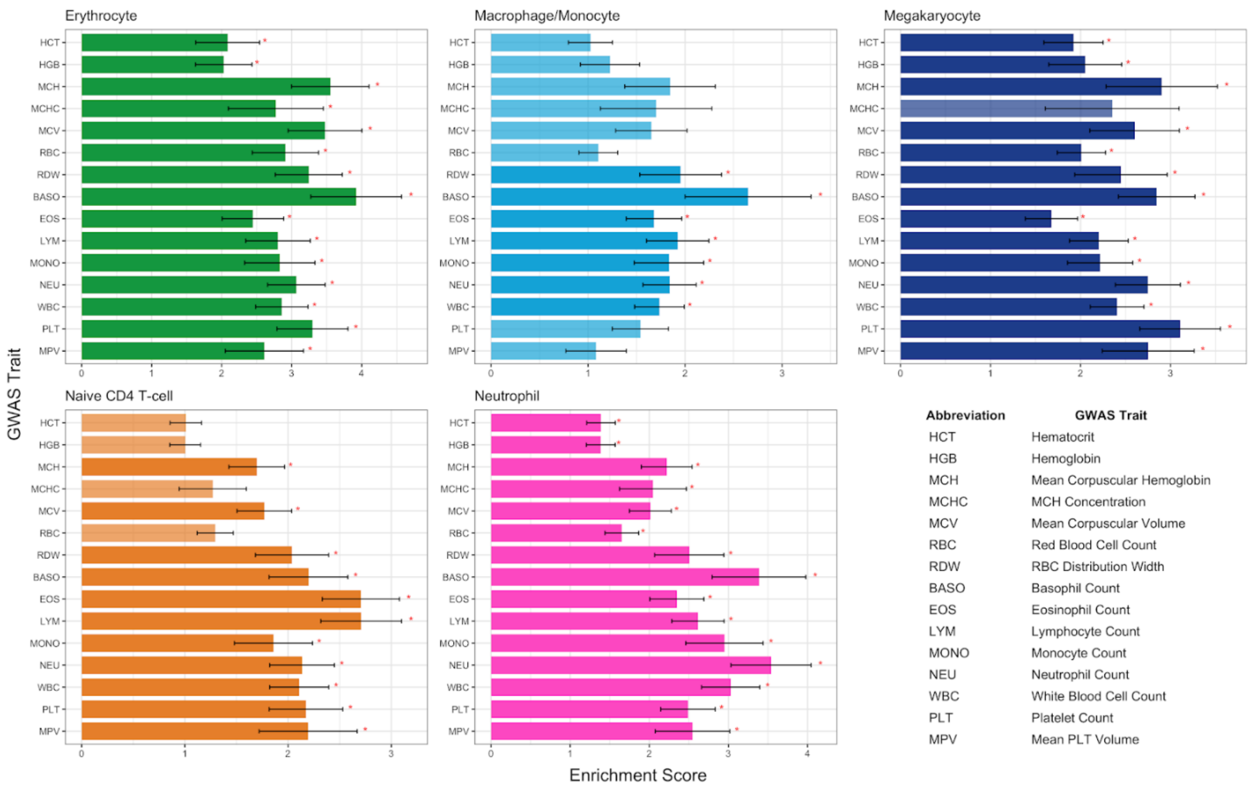

S6 Fig. Partitioned SNP heritability–for blood cell traits - all SIP PIRs. Enrichment scores for SIPs and 15 blood cell traits. (* denotes statistically significant enrichment score ($p < 0.05$); Ery = erythrocytes; MacMon = macrophages/monocytes; MK = megakaryocytes; nCD4 = naive CD4 T-cells; Neu = neutrophils)
