# Supplementary material for: Super interactive promoters provide insight into cell type-specific regulatory networks in blood lineage cell types
Source: PLoS Genet. 2022 Jan 31;18(1):e1009984. doi: 10.1371/journal.pgen.1009984 (PMC8830683; doi:10.1371/journal.pgen.1009984)
Supplement: S7 Fig — Violin plots showing the distribution of gene expression of shared SIP genes in various tissues as well as the five blood cell types. (A) Shared SIP genes with the top 10% of expression among blood cells. (B) Shared SIP genes with the top 10–20% of expression among blood cells. (C) Shared SIP genes with the top 10% of expression among other tissues (non-blood cells). (D) Shared SIP genes with the top 10–20% of expression among other tissues (non-blood cells). (Ery = erythrocytes; MacMon = macrophages/monocytes; MK = megakaryocytes; nCD4 = naive CD4 T-cells; Neu = neutrophils). (PDF) [file pgen.1009984.s009.pdf]

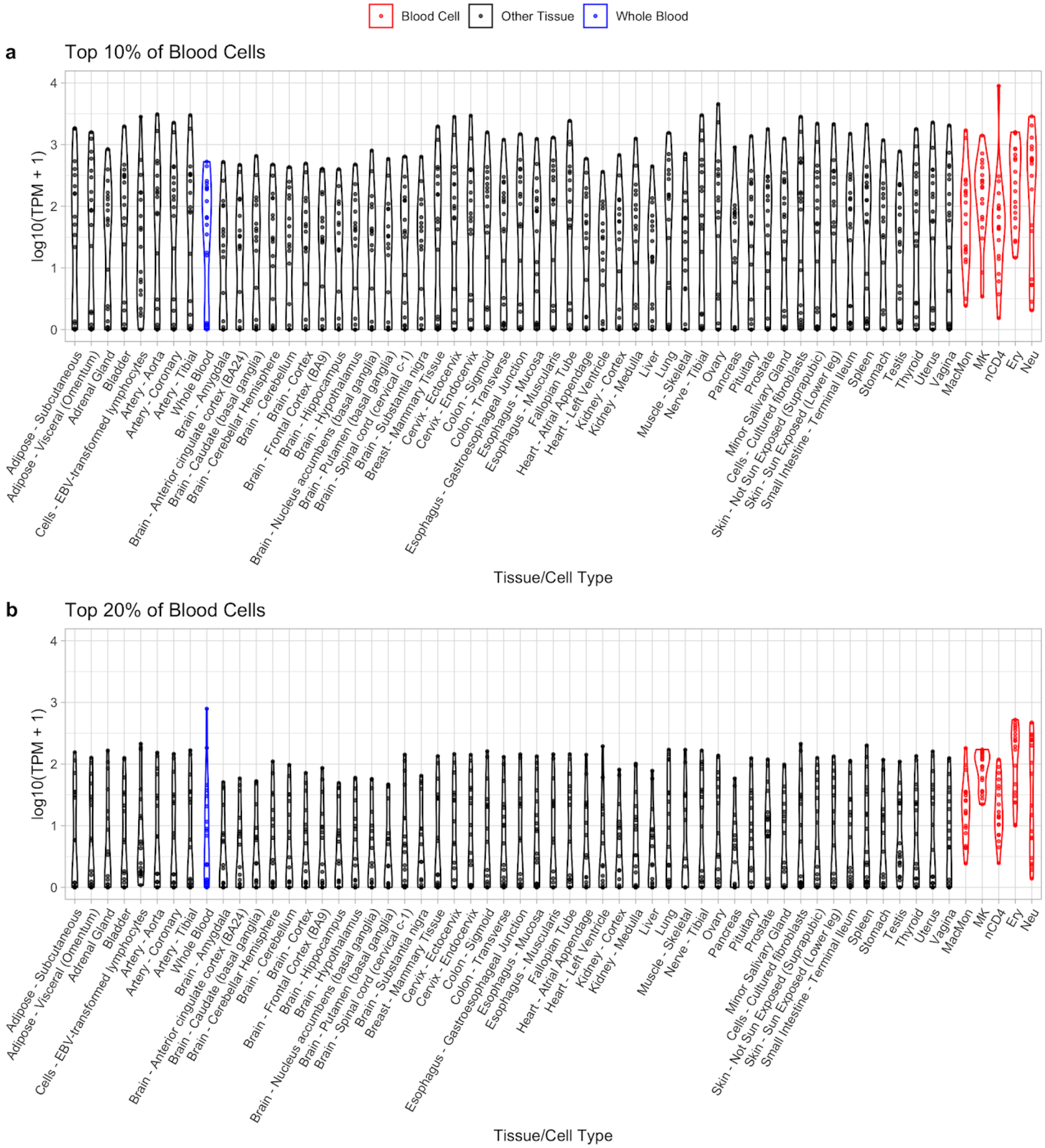
Blood Cell
Other Tissue
Whole Blood
a
Top 10% of Blood Cells
log10(TPM + 1)
0
1
2
3
4
Adipose - Subcutaneous
Adipose - Visceral (Omentum)
Adrenal Gland
Bladder
Cells - EBV-transformed lymphocytes
Artery - Aorta
Artery - Coronary
Artery - Tibial
Whole Blood
Brain - Amygdala
Brain - Anterior cingulate cortex (BA24)
Brain - Caudate (basal ganglia)
Brain - Cerebellar Hemisphere
Brain - Cerebellum
Brain - Cortex
Brain - Frontal Cortex (BA9)
Brain - Hippocampus
Brain - Hypothalamus
Brain - Nucleus accumbens (basal ganglia)
Brain - Putamen (basal ganglia)
Brain - Spinal cord (cervical c-1)
Brain - Substantia nigra
Breast - Mammary Tissue
Cervix - Ectocervix
Cervix - Endocervix
Colon - Sigmoid
Colon - Transverse
Esophagus - Gastroesophageal Junction
Esophagus - Mucosa
Esophagus - Muscularis
Fallopian Tube
Heart - Atrial Appendage
Heart - Left Ventricle
Kidney - Cortex
Kidney - Medulla
Liver
Lung
Muscle - Skeletal
Nerve - Tibial
Ovary
Pancreas
Pituitary
Prostate
Minor Salivary Gland
Cells - Cultured fibroblasts
Skin - Not Sun Exposed (Suprapubic)
Skin - Sun Exposed (Lower leg)
Small Intestine - Terminal Ileum
Spleen
Stomach
Testis
Thyroid
Uterus
Vagina
MacMon
MK
nCD4
Ery
Neu
Tissue/Cell Type
b
Top 20% of Blood Cells
log10(TPM + 1)
0
1
2
3
4
Adipose - Subcutaneous
Adipose - Visceral (Omentum)
Adrenal Gland
Bladder
Cells - EBV-transformed lymphocytes
Artery - Aorta
Artery - Coronary
Artery - Tibial
Whole Blood
Brain - Amygdala
Brain - Anterior cingulate cortex (BA24)
Brain - Caudate (basal ganglia)
Brain - Cerebellar Hemisphere
Brain - Cerebellum
Brain - Cortex
Brain - Frontal Cortex (BA9)
Brain - Hippocampus
Brain - Hypothalamus
Brain - Nucleus accumbens (basal ganglia)
Brain - Putamen (basal ganglia)
Brain - Spinal cord (cervical c-1)
Brain - Substantia nigra
Breast - Mammary Tissue
Cervix - Ectocervix
Cervix - Endocervix
Colon - Sigmoid
Colon - Transverse
Esophagus - Gastroesophageal Junction
Esophagus - Mucosa
Esophagus - Muscularis
Fallopian Tube
Heart - Atrial Appendage
Heart - Left Ventricle
Kidney - Cortex
Kidney - Medulla
Liver
Lung
Muscle - Skeletal
Nerve - Tibial
Ovary
Pancreas
Pituitary
Prostate
Minor Salivary Gland
Cells - Cultured fibroblasts
Skin - Not Sun Exposed (Suprapubic)
Skin - Sun Exposed (Lower leg)
Small Intestine - Terminal Ileum
Spleen
Stomach
Testis
Thyroid
Uterus
Vagina
MacMon
MK
nCD4
Ery
Neu
Tissue/Cell Type

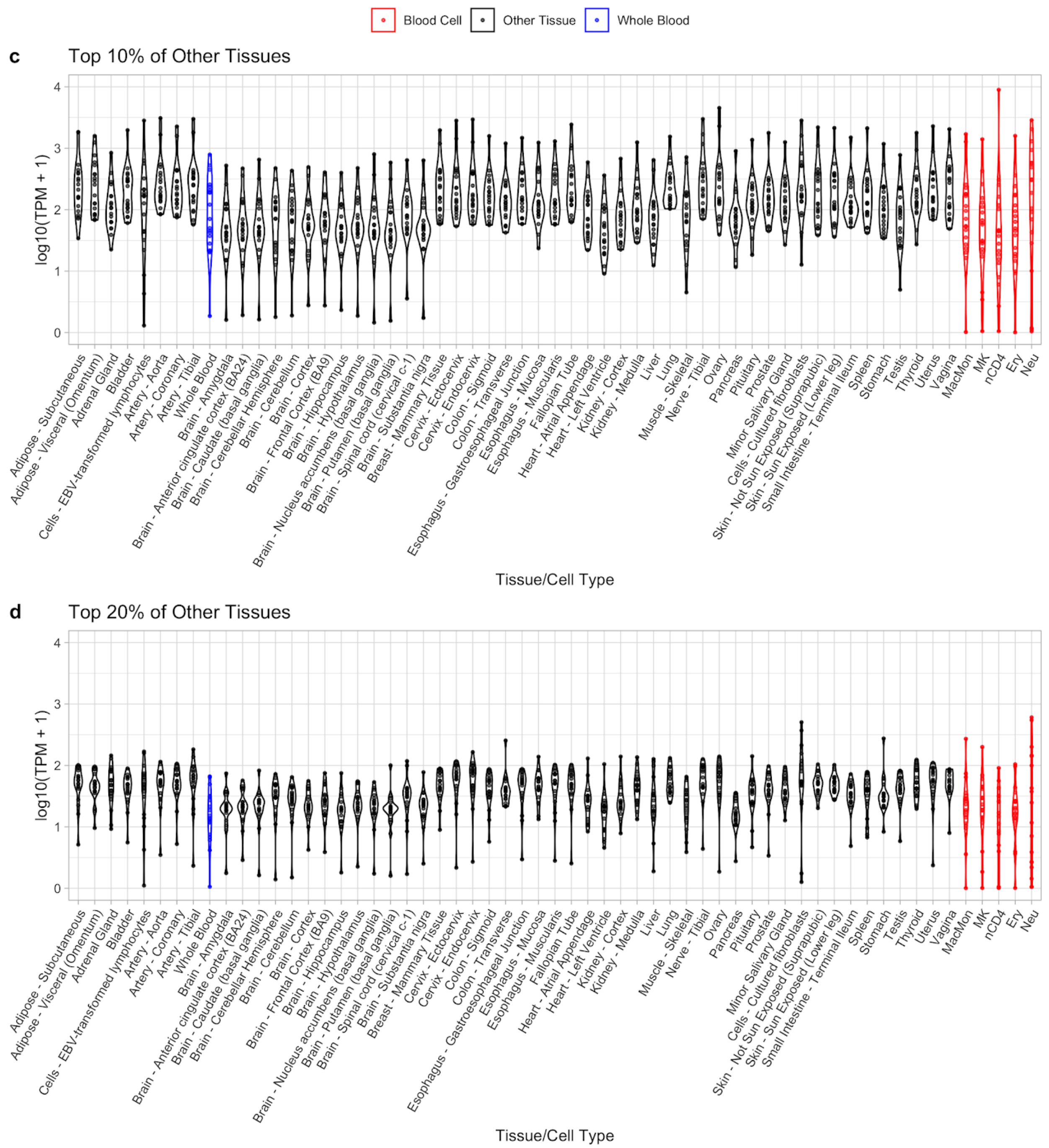

S7 Fig. Shared SIP genes have elevated expression levels in hematopoietic cell types. Violin plots showing the distribution of gene expression of shared SIP genes in various tissues as well as the five blood cell types. **(A)** Shared SIP genes with the top 10% of expression among blood cells. **(B)** Shared SIP genes with the top 10-20% of expression among blood cells. **(C)** Shared SIP genes with the top 10% of expression among other tissues (non-blood cells). **(D)** Shared SIP genes with the top 10-20% of

expression among other tissues (non-blood cells). (Ery = erythrocytes; MacMon = macrophages/monocytes; MK = megakaryocytes; nCD4 = naive CD4 T-cells; Neu = neutrophils)
