# Supplementary material for: Super interactive promoters provide insight into cell type-specific regulatory networks in blood lineage cell types
Source: PLoS Genet. 2022 Jan 31;18(1):e1009984. doi: 10.1371/journal.pgen.1009984 (PMC8830683; doi:10.1371/journal.pgen.1009984)
Supplement: S8 Fig — Violin plots showing the distribution of gene expression of neutrophil-specific SIP genes in various tissues as well as the five blood cell types. The cell type-specific SIP genes for erythrocytes, macrophages/monocytes, and naive CD4 T-cells show similar trends. (A) Neutrophil-specific SIP genes with the top 10% of neutrophils expression. (B) Neutrophil-specific SIP genes with the top 10–20% of neutrophils expression. (C) Neutrophil-specific SIP genes with the top 10% of expression among other tissues (non-blood cells). (D) Neutrophil-specific SIP genes with the top 10–20% of expression among other tissues (non-blood cells). (Ery = erythrocytes; MacMon = macrophages/monocyItes; MK = megakaryocytes; nCD4 = naive CD4 T-cells; Neu = neutrophils). (PDF) [file pgen.1009984.s010.pdf]

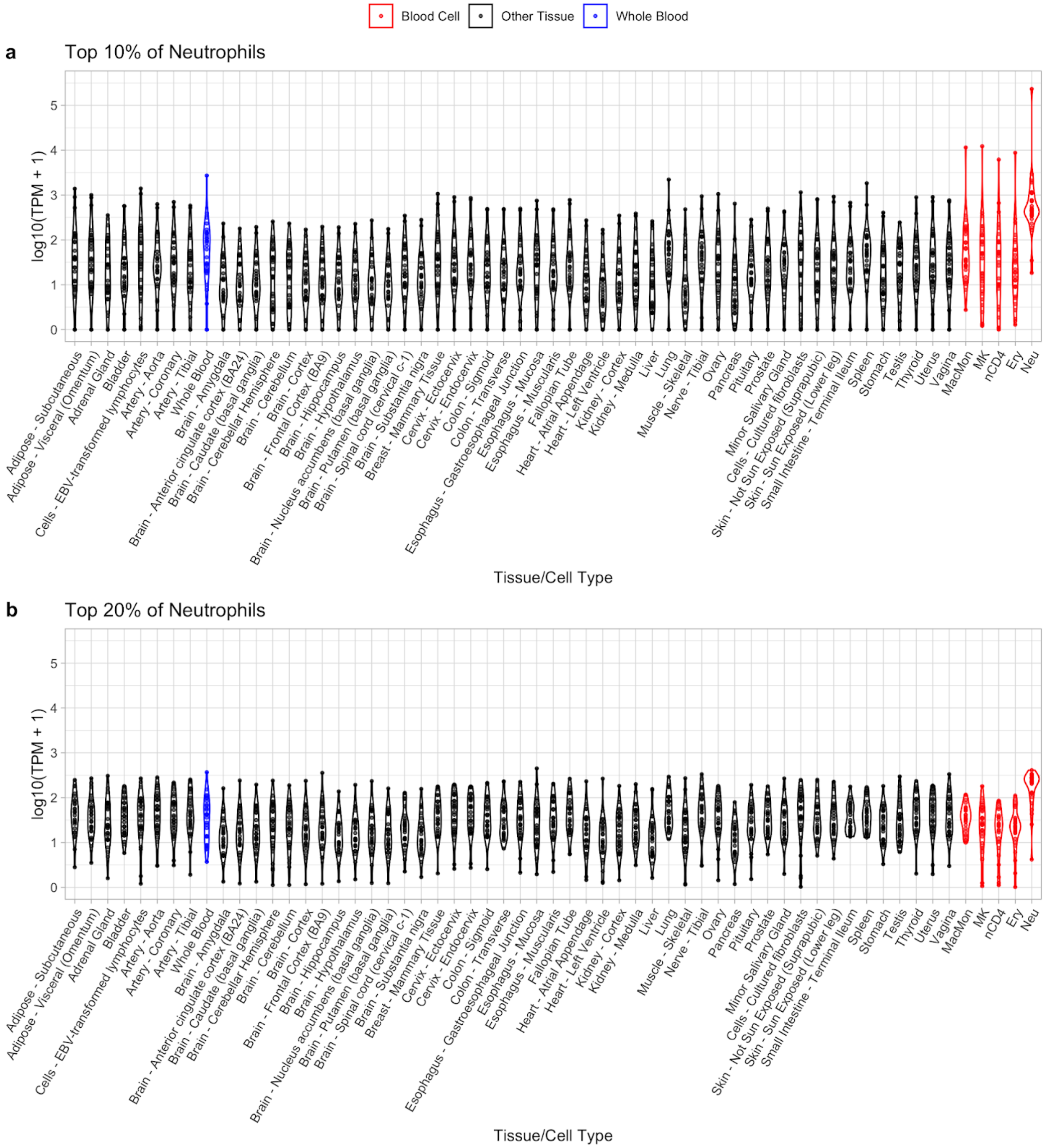
Blood Cell
Other Tissue
Whole Blood
a
Top 10% of Neutrophils
log10(TPM + 1)
0
1
2
3
4
5
Adipose - Subcutaneous
Adipose - Visceral (Omentum)
Adrenal Gland
Bladder
Cells - EBV-transformed lymphocytes
Artery - Aorta
Artery - Coronary
Artery - Tibial
Whole Blood
Brain - Amygdala
Brain - Anterior cingulate cortex (BA24)
Brain - Caudate (basal ganglia)
Brain - Cerebellar Hemisphere
Brain - Cerebellum
Brain - Cortex
Brain - Frontal Cortex (BA9)
Brain - Hippocampus
Brain - Hypothalamus
Brain - Nucleus accumbens (basal ganglia)
Brain - Putamen (basal ganglia)
Brain - Spinal cord (cervical c-1)
Brain - Substantia nigra
Breast - Mammary Tissue
Cervix - Ectocervix
Cervix - Endocervix
Colon - Sigmoid
Colon - Transverse
Esophagus - Gastroesophageal Junction
Esophagus - Mucosa
Esophagus - Muscularis
Fallopian Tube
Heart - Atrial Appendage
Heart - Left Ventricle
Kidney - Cortex
Kidney - Medulla
Liver
Lung
Muscle - Skeletal
Nerve - Tibial
Ovary
Pancreas
Pituitary
Prostate
Minor Salivary Gland
Cells - Cultured fibroblasts
Skin - Not Sun Exposed (Suprapubic)
Skin - Sun Exposed (Lower leg)
Small Intestine - Terminal Ileum
Spleen
Stomach
Testis
Thyroid
Uterus
Vagina
MacMon
MK
nCD4
Ery
Neu
Tissue/Cell Type
b
Top 20% of Neutrophils
log10(TPM + 1)
0
1
2
3
4
5
Adipose - Subcutaneous
Adipose - Visceral (Omentum)
Adrenal Gland
Bladder
Cells - EBV-transformed lymphocytes
Artery - Aorta
Artery - Coronary
Artery - Tibial
Whole Blood
Brain - Amygdala
Brain - Anterior cingulate cortex (BA24)
Brain - Caudate (basal ganglia)
Brain - Cerebellar Hemisphere
Brain - Cerebellum
Brain - Cortex
Brain - Frontal Cortex (BA9)
Brain - Hippocampus
Brain - Hypothalamus
Brain - Nucleus accumbens (basal ganglia)
Brain - Putamen (basal ganglia)
Brain - Spinal cord (cervical c-1)
Brain - Substantia nigra
Breast - Mammary Tissue
Cervix - Ectocervix
Cervix - Endocervix
Colon - Sigmoid
Colon - Transverse
Esophagus - Gastroesophageal Junction
Esophagus - Mucosa
Esophagus - Muscularis
Fallopian Tube
Heart - Atrial Appendage
Heart - Left Ventricle
Kidney - Cortex
Kidney - Medulla
Liver
Lung
Muscle - Skeletal
Nerve - Tibial
Ovary
Pancreas
Pituitary
Prostate
Minor Salivary Gland
Cells - Cultured fibroblasts
Skin - Not Sun Exposed (Suprapubic)
Skin - Sun Exposed (Lower leg)
Small Intestine - Terminal Ileum
Spleen
Stomach
Testis
Thyroid
Uterus
Vagina
MacMon
MK
nCD4
Ery
Neu
Tissue/Cell Type

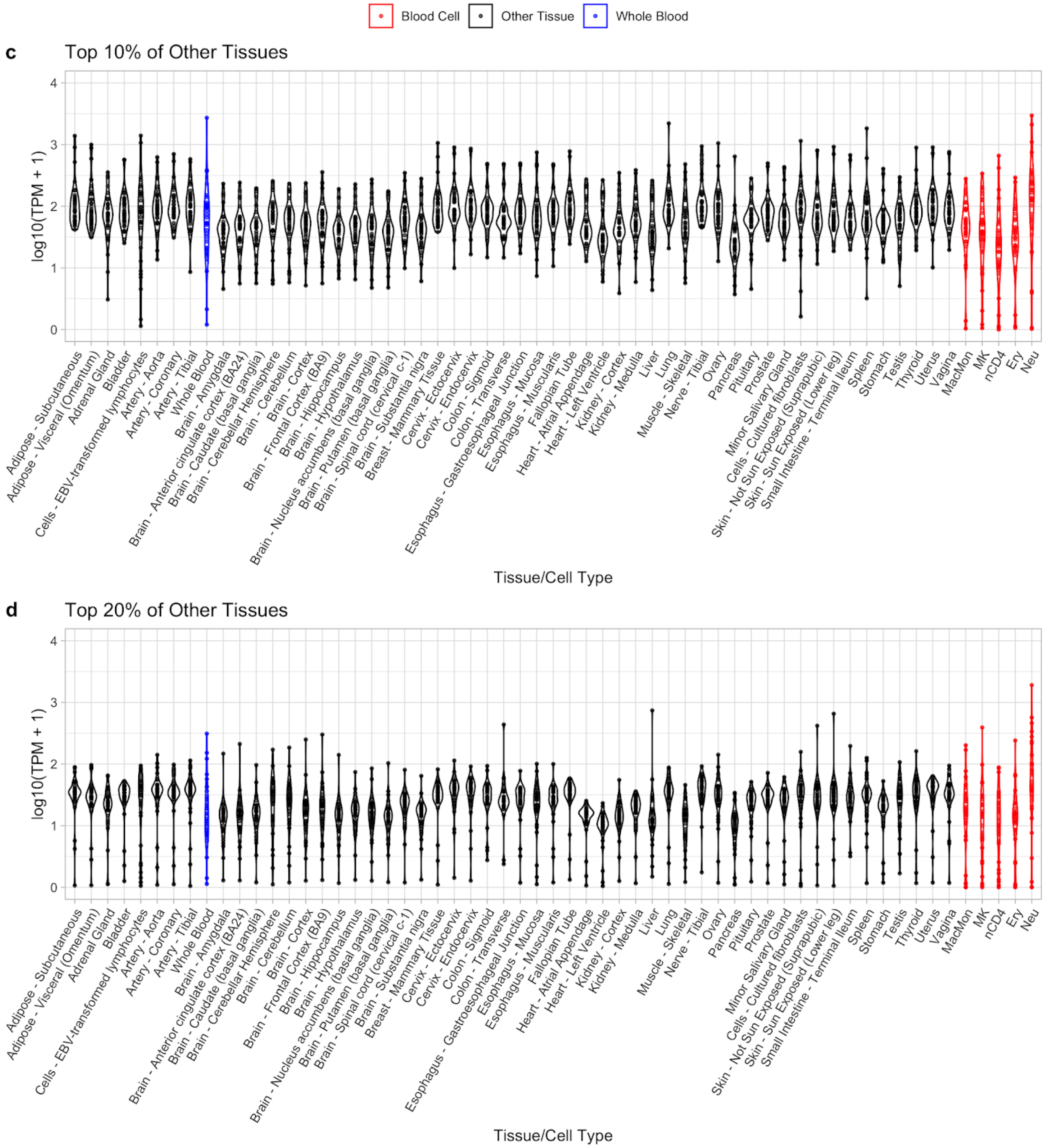

S8 Fig. Neutrophil specific SIP genes have elevated expression levels in neutrophils. Violin plots showing the distribution of gene expression of neutrophil-specific SIP genes in various tissues as well as the five blood cell types. The cell type-specific SIP genes for erythrocytes, macrophages/monocytes, and naive CD4 T-cells show similar trends. **(A)** Neutrophil-specific SIP genes with the top 10% of neutrophils

expression. **(B)** Neutrophil-specific SIP genes with the top 10-20% of neutrophils expression. **(C)** Neutrophil-specific SIP genes with the top 10% of expression among other tissues (non-blood cells). **(D)** Neutrophil-specific SIP genes with the top 10-20% of expression among other tissues (non-blood cells). (Ery = erythrocytes; MacMon = macrophages/monocyItes; MK = megakaryocytes; nCD4 = naive CD4 T-cells; Neu = neutrophils)
