# Supplementary material for: Super interactive promoters provide insight into cell type-specific regulatory networks in blood lineage cell types
Source: PLoS Genet. 2022 Jan 31;18(1):e1009984. doi: 10.1371/journal.pgen.1009984 (PMC8830683; doi:10.1371/journal.pgen.1009984)
Supplement: S9 Fig — (PDF) [file pgen.1009984.s011.pdf]

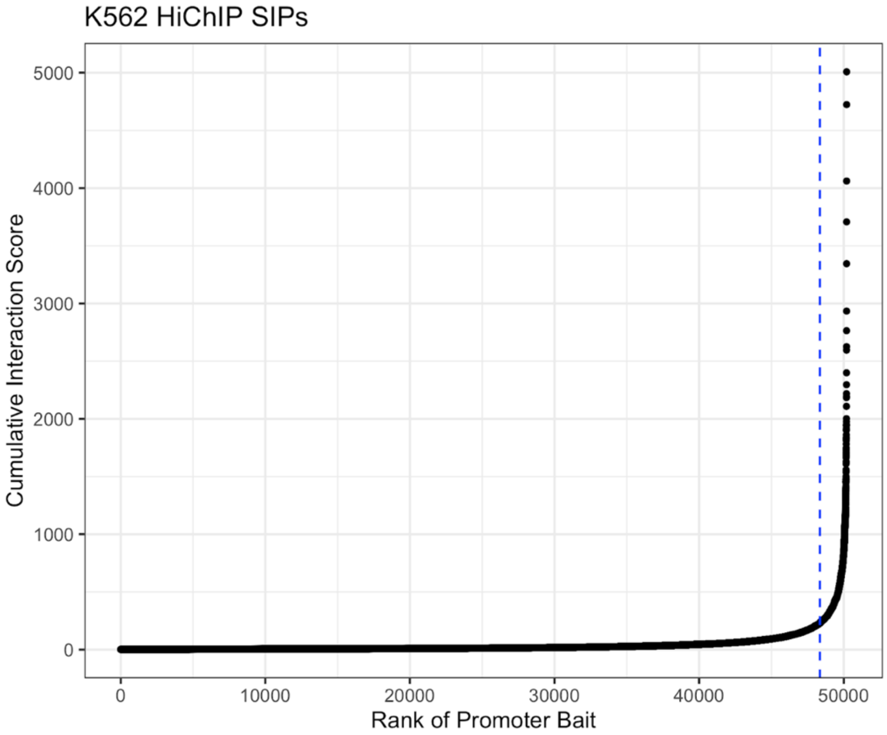

S9 Fig. Hockey plots for each cell type show the ranked cumulative interaction MAP scores for K562 SIPs (baits)
