# Supplementary material for: Super interactive promoters provide insight into cell type-specific regulatory networks in blood lineage cell types
Source: PLoS Genet. 2022 Jan 31;18(1):e1009984. doi: 10.1371/journal.pgen.1009984 (PMC8830683; doi:10.1371/journal.pgen.1009984)
Supplement: S10 Fig — Distribution of the number of significant interactions (log10 scale) between promoter bait and promoter interacting regions (PIRs) for SIPs and non-SIPs in each cell type. The median of each distribution is marked by a black dot. (PDF) [file pgen.1009984.s012.pdf]

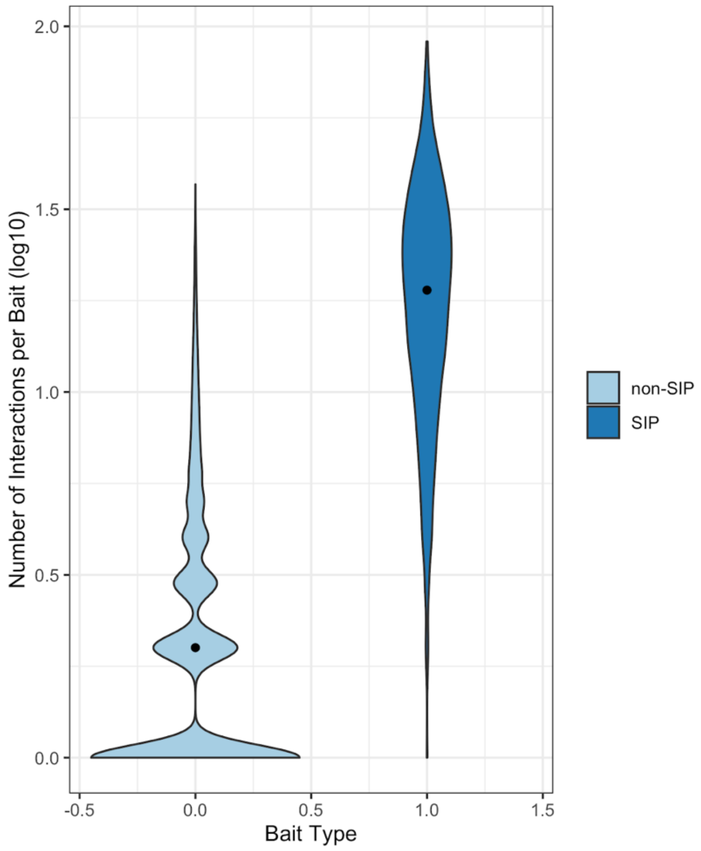

S10 Fig. K562 SIPs are driven by a large number of interactions. Distribution of the number of significant interactions (log10 scale) between promoter bait and promoter interacting regions (PIRs) for SIPs and non-SIPs in each cell type. The median of each distribution is marked by a black dot.
