# Supplementary material for: Super interactive promoters provide insight into cell type-specific regulatory networks in blood lineage cell types
Source: PLoS Genet. 2022 Jan 31;18(1):e1009984. doi: 10.1371/journal.pgen.1009984 (PMC8830683; doi:10.1371/journal.pgen.1009984)
Supplement: S11 Fig — The median of each distribution is marked by a black dot. (PDF) [file pgen.1009984.s013.pdf]

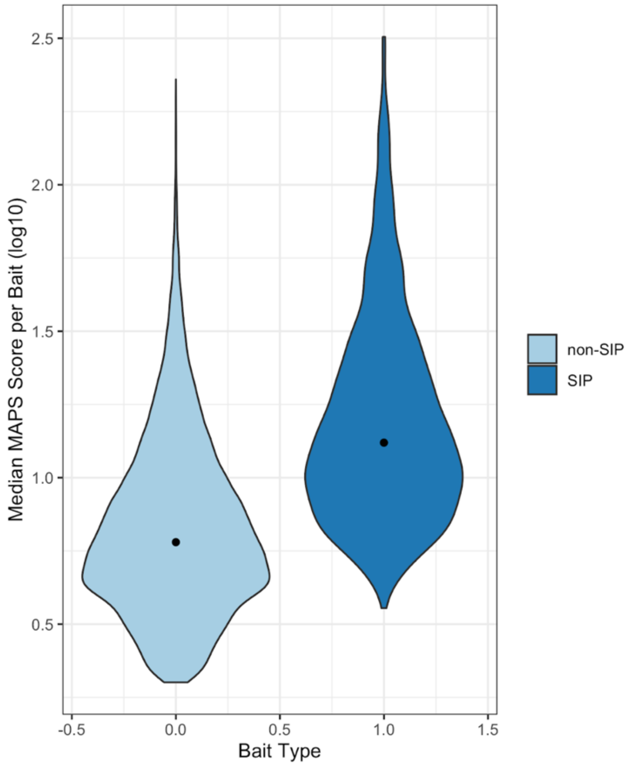

S11 Fig. The Distribution of the median MAP score (log10 scale) of significant interactions per promoter bait for SIPs and non-SIPs in K562. The median of each distribution is marked by a black dot.
