# Supplementary material for: Super interactive promoters provide insight into cell type-specific regulatory networks in blood lineage cell types
Source: PLoS Genet. 2022 Jan 31;18(1):e1009984. doi: 10.1371/journal.pgen.1009984 (PMC8830683; doi:10.1371/journal.pgen.1009984)
Supplement: S12 Fig — A. Highly expressed genes exhibit higher level of chromatin interactions in a cell type specific manner. In each cell type, genes with the highest (1st) rank in expression show significantly higher chromatin interaction (as measured by SIP score) than those with the lowest (5th) ranked. The size of the circle denotes the fold-change (in SIP scores, quantifying the level of chromatin interaction) and the color denotes the Chi-square significance of enrichment (again for SIP scores). The Cochran-Armitage trend test p-value (one-sided) is also reported. B. SIP genes detected in a cell type are more likely to be highly expressed in that cell type (Wilcoxon test p-value = 2.95×10^−88^ ~ 7.14×10^−13^). c. SIPs overlapping ATAC-seq peaks are more likely to be highly expressed than those not overlapping. (Wilcoxon test p-value = 4.23×10^−40^~ 7.15×10^−16^) (Ery = erythrocytes; MacMon = macrophages/monocytes; MK = megakaryocytes; nCD4 = naive CD4 T-cells; Neu = neutrophils). (PDF) [file pgen.1009984.s014.pdf]

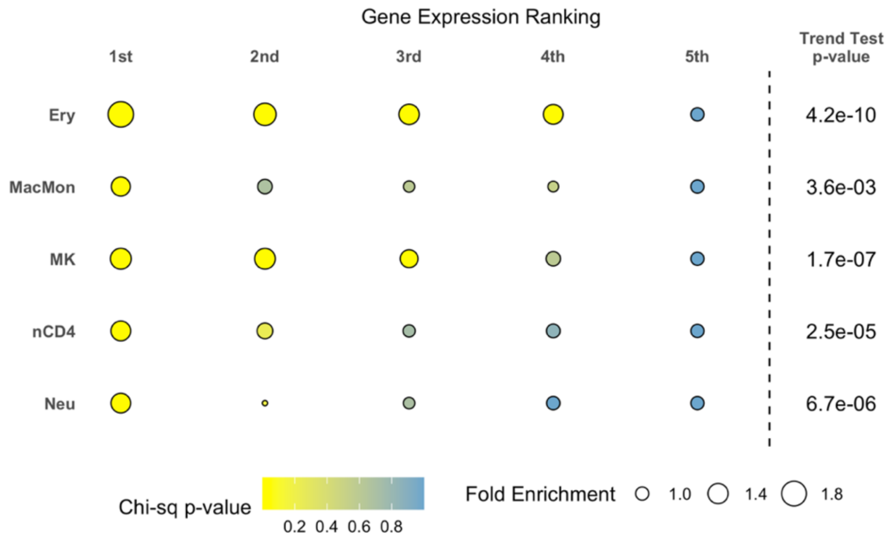
Gene Expression Ranking
1st
2nd
3rd
4th
5th
Trend Test p-value
Ery
MacMon
MK
nCD4
Neu
4.2e-10
3.6e-03
1.7e-07
2.5e-05
6.7e-06
Chi-sq p-value
0.2 0.4 0.6 0.8
Fold Enrichment
1.0
1.4
1.8

a.

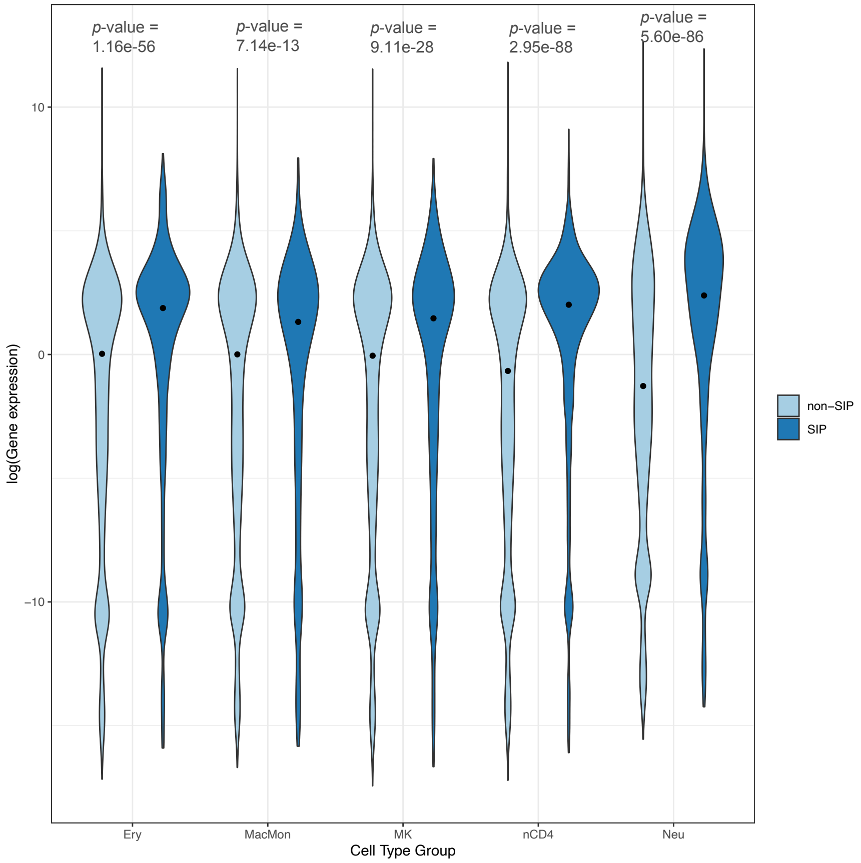
p-value = 1.16e-56
p-value = 7.14e-13
p-value = 9.11e-28
p-value = 2.95e-88
p-value = 5.60e-86
10
0
−10
log(Gene expression)
Ery
MacMon
MK
nCD4
Neu
Cell Type Group
non−SIP
SIP

b.

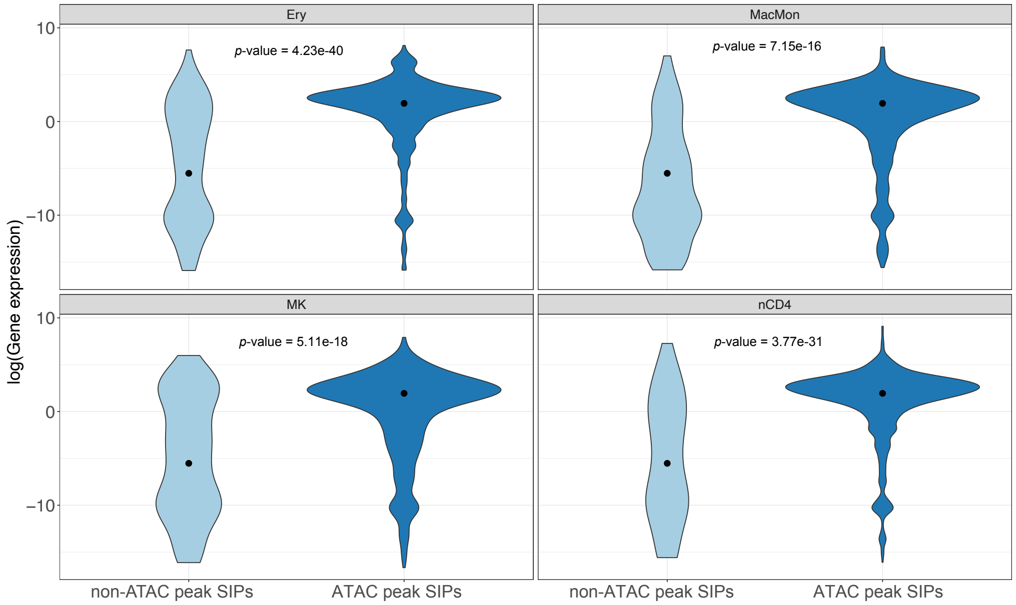

c.

S12 Fig. (A) Highly expressed genes exhibit higher level of chromatin interactions in a cell type specific manner. In each cell type, genes with the highest (1st) rank in expression show significantly higher chromatin interaction (as measured by SIP score) than those with the lowest (5th) ranked. The size of the circle denotes the fold-change (in SIP scores, quantifying the level of chromatin interaction) and the color denotes the Chi-square significance of enrichment (again for SIP scores). The Cochran-Armitage trend test *p*-value (one-sided) is also reported. **(B) SIP genes detected in a cell type are more likely to be highly expressed in that cell type** (Wilcoxon test *p*-value = $2.95\times10^{-88} \sim 7.14\times10^{-13}$). **(C) SIPs overlapping ATAC-seq peaks are more likely to be highly expressed than those not overlapping.** (Wilcoxon test *p*-value = $4.23\times10^{-40}\sim 7.15\times10^{-16}$) (Ery = erythrocytes; MacMon = macrophages/monocytes; MK = megakaryocytes; nCD4 = naive CD4 T-cells; Neu = neutrophils)
