# Supplementary material for: Super interactive promoters provide insight into cell type-specific regulatory networks in blood lineage cell types
Source: PLoS Genet. 2022 Jan 31;18(1):e1009984. doi: 10.1371/journal.pgen.1009984 (PMC8830683; doi:10.1371/journal.pgen.1009984)
Supplement: S13 Fig — A. GM12878 SIPs are driven by a large number of interactions. Distribution of the number of significant interactions (log10 scale) between promoter bait and promoter interacting regions (PIRs) for SIPs and non-SIPs in GM12878 HiChIP. B. The Distribution of the median SIP score (-log10 MAPS q-value) of significant interactions per promoter bait for SIPs and non-SIPs in GM12878 HiChIP. C. Distribution of the number of significant interactions (log10 scale) between promoter bait and promoter interacting regions (PIRs) for SIPs and non-SIPs in GM12878 Hi-C. D. The Distribution of the median SIP score (-log10 q-value) of significant interactions per promoter bait for SIPs and non-SIPs in GM12878 Hi-C. The median of each distribution is marked by a black dot. (PDF) [file pgen.1009984.s015.pdf]

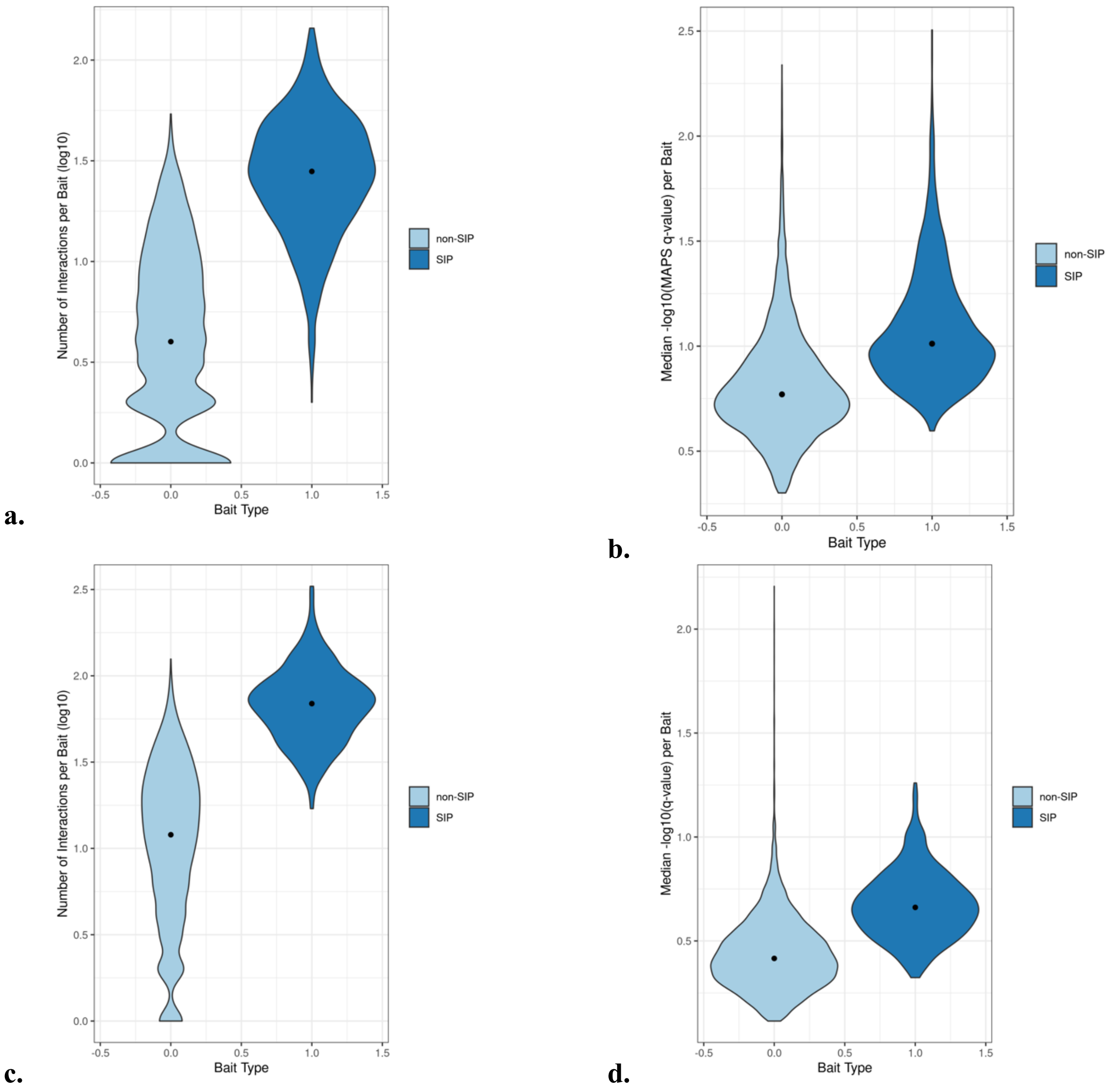

S13 Fig. (A) GM12878 SIPs are driven by a large number of interactions. Distribution of the number of significant interactions (log10 scale) between promoter bait and promoter interacting regions (PIRs) for SIPs and non-SIPs in GM12878 HiChIP. **(B)** The Distribution of the median SIP score (-log10 MAPS q-value) of significant interactions per promoter bait for SIPs and non-SIPs in GM12878 HiChIP**. (C)** Distribution of the number of significant interactions (log10 scale) between promoter bait and promoter interacting regions (PIRs) for SIPs and non-SIPs in GM12878 Hi-C**. (D)** The Distribution of the median SIP score (-log10 q-value) of significant interactions per promoter bait for SIPs and non-SIPs in GM12878 Hi-C. The median of each distribution is marked by a black dot.
