# Supplementary material for: Super interactive promoters provide insight into cell type-specific regulatory networks in blood lineage cell types
Source: PLoS Genet. 2022 Jan 31;18(1):e1009984. doi: 10.1371/journal.pgen.1009984 (PMC8830683; doi:10.1371/journal.pgen.1009984)
Supplement: S14 Fig — Transcription factor motif enrichment analysis for A. SIPs versus non-SIPs. B. SIP PIRs versus non-SIP PIRs. The Y-axis shows the transcription factor and X-axis shows odds ratio of enrichment. The dot denotes the point estimate of odds ratio and the line denotes the lower 95% confidence. (PDF) [file pgen.1009984.s016.pdf]

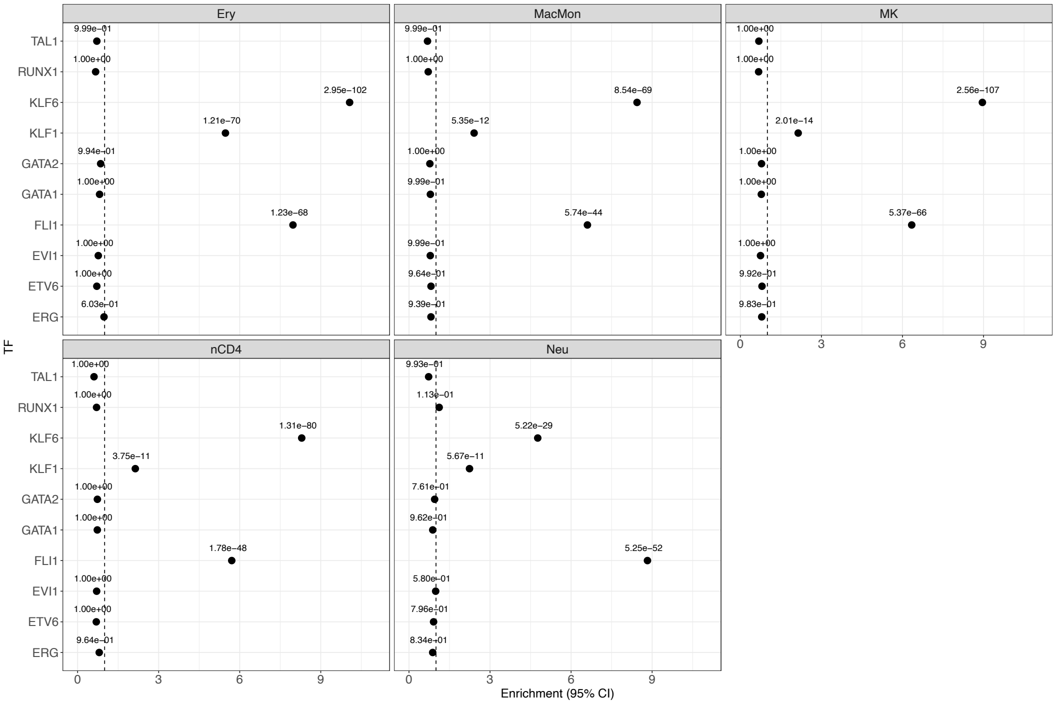

a.

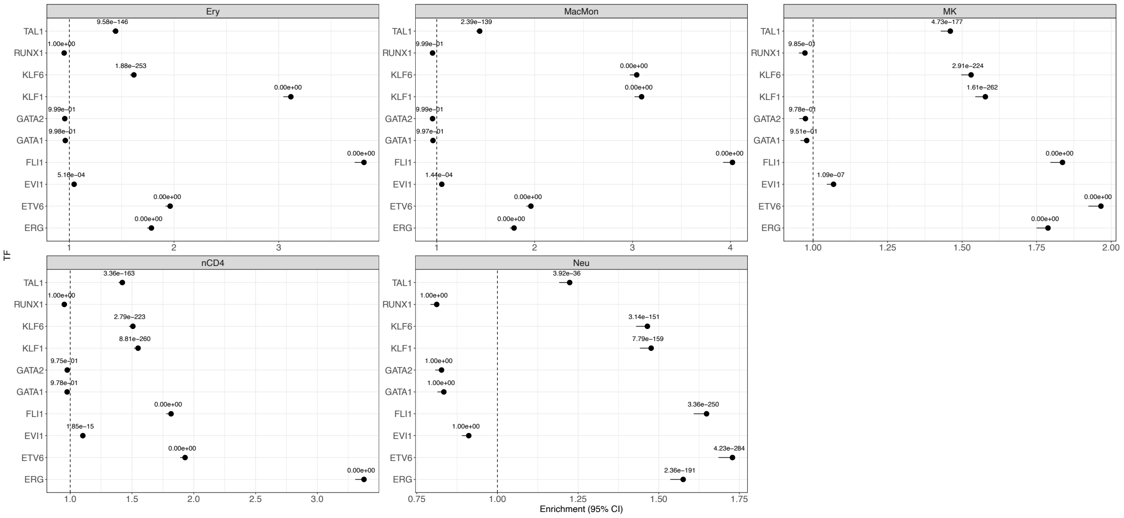

b.

S14 Fig. Transcription factor motif enrichment analysis for **(A)** SIPs versus non-SIPs. (**B**) SIP PIRs versus non-SIP PIRs. The Y-axis shows the transcription factor and X-axis shows odds ratio of enrichment. The dot denotes the point estimate of odds ratio and the line denotes the lower 95% confidence.
