# Supplementary material for: Super interactive promoters provide insight into cell type-specific regulatory networks in blood lineage cell types
Source: PLoS Genet. 2022 Jan 31;18(1):e1009984. doi: 10.1371/journal.pgen.1009984 (PMC8830683; doi:10.1371/journal.pgen.1009984)
Supplement: S15 Fig — Distributions of distance to TAD boundaries for non-SIPs versus SIPs are visualized using side-by-side violin plots (left) and overlapping density plots (right). (PDF) [file pgen.1009984.s017.pdf]

Ery SIPs vs non-SIPs

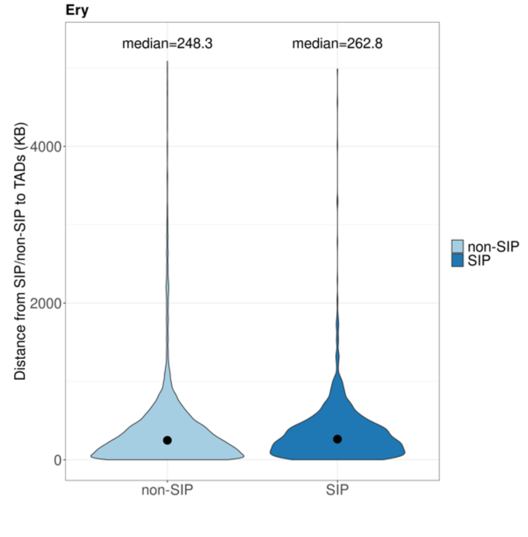

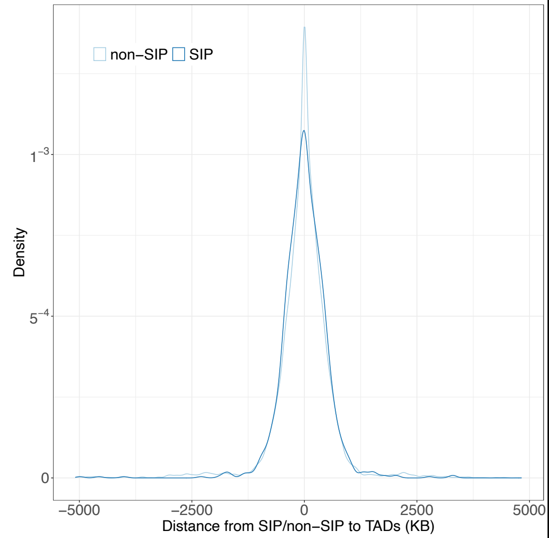

MacMon SIPs vs non-SIPs

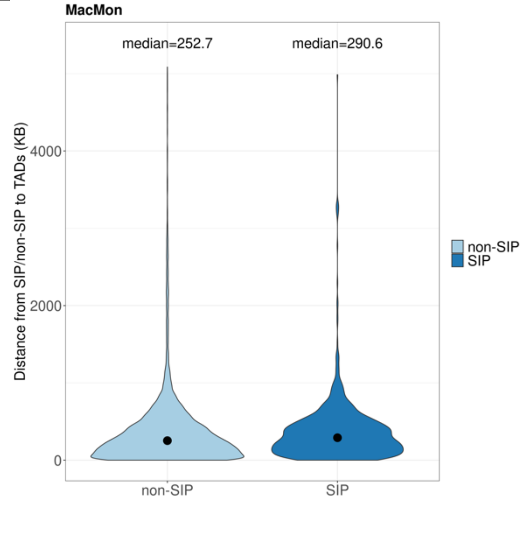

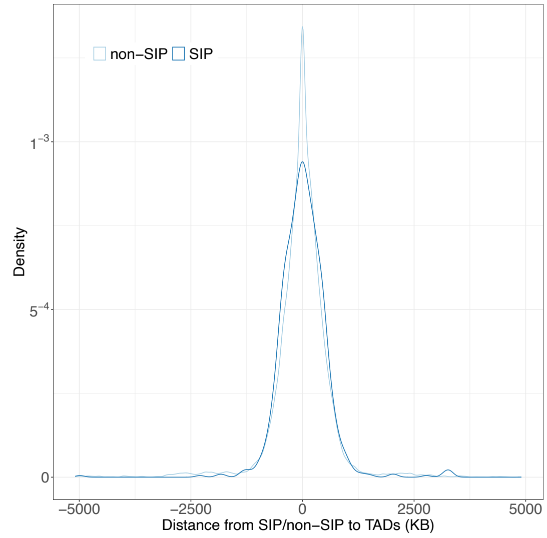

MK SIPs vs non-SIPs

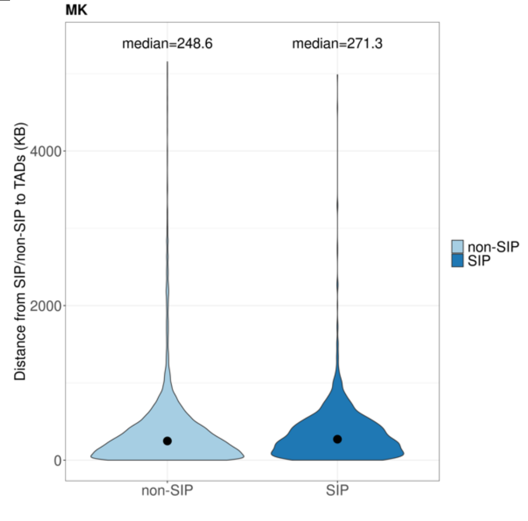

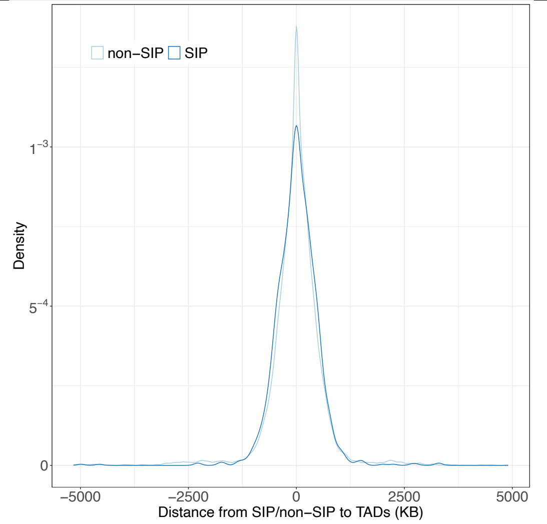

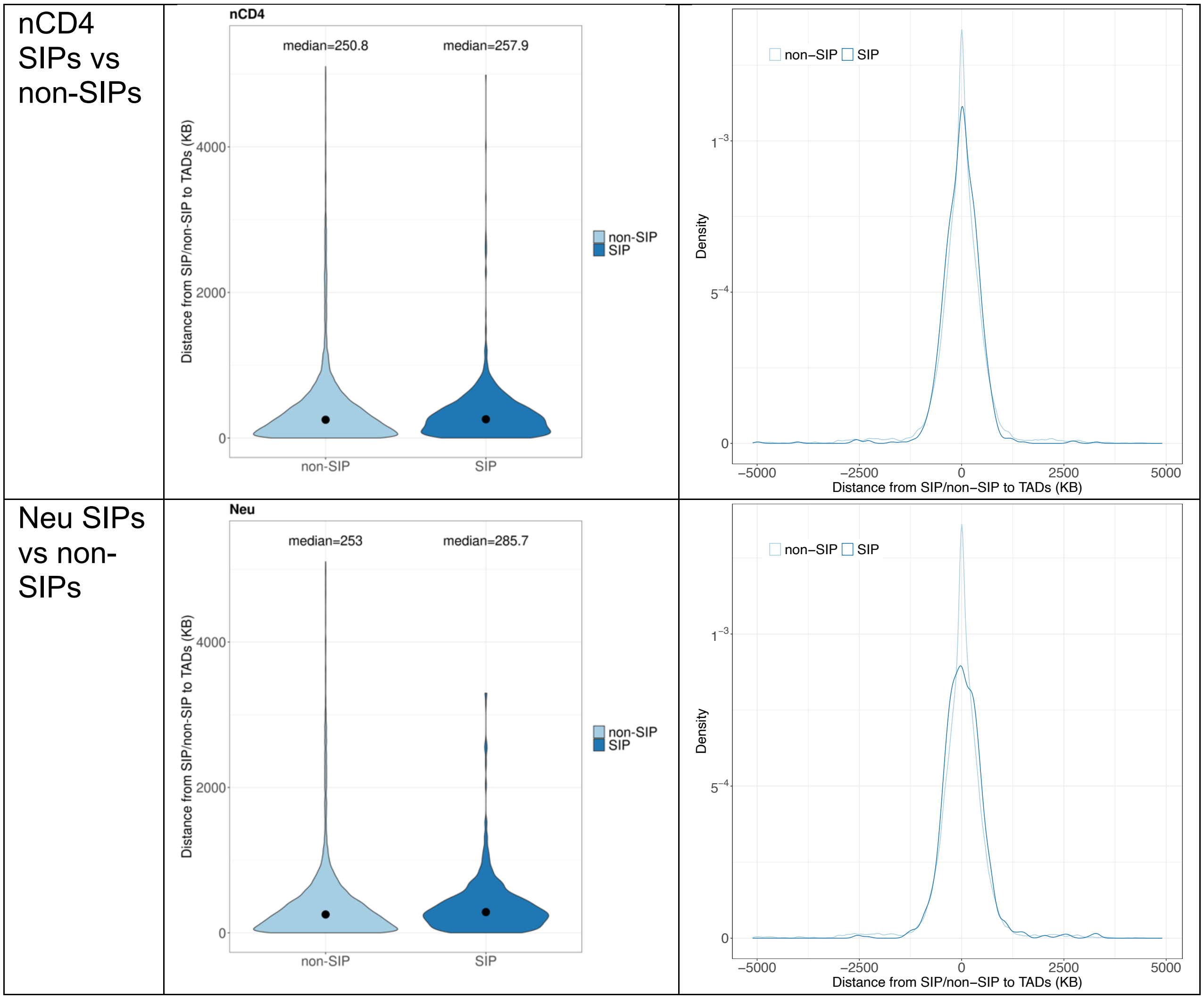

S15 Fig. Distance to TAD boundary for SIPs versus non-SIPs. Distributions of distance to TAD boundaries for non-SIPs versus SIPs are visualized using side-by-side violin plots (left) and overlapping density plots (right).
