# Supplementary material for: Super interactive promoters provide insight into cell type-specific regulatory networks in blood lineage cell types
Source: PLoS Genet. 2022 Jan 31;18(1):e1009984. doi: 10.1371/journal.pgen.1009984 (PMC8830683; doi:10.1371/journal.pgen.1009984)
Supplement: S16 Fig — Distributions of distance to TAD boundaries for non-SIP PIRs versus SIP PIRs are visualized using side-by-side violin plots (left) and overlapping density plots (right). (PDF) [file pgen.1009984.s018.pdf]

Ery SIP PIRs vs non-SIP PIRs

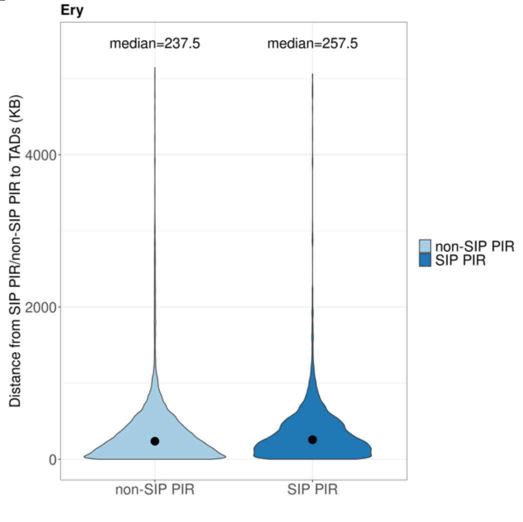

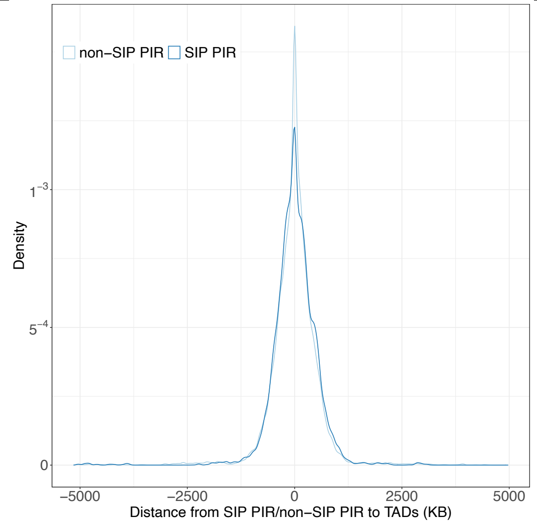

MacMon SIP PIRs vs non-SIP PIRs

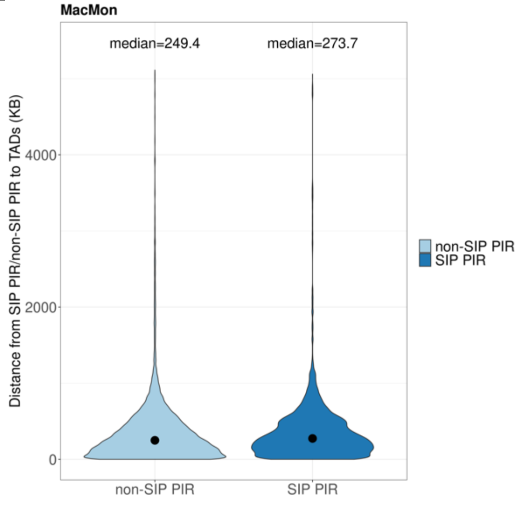

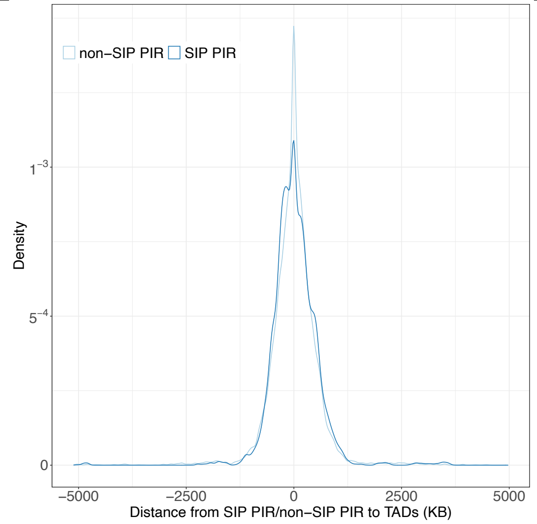

MK SIP PIRs vs non-SIP PIRs

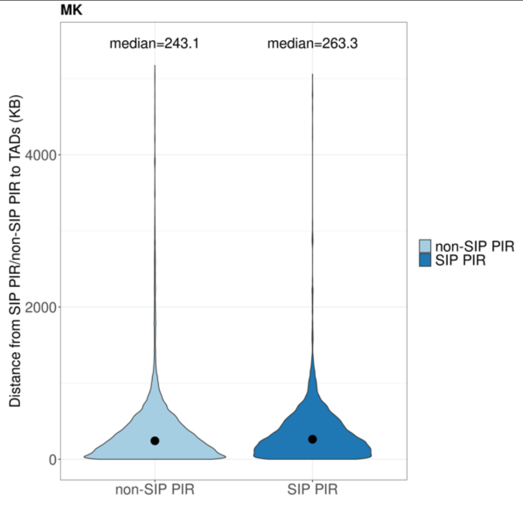

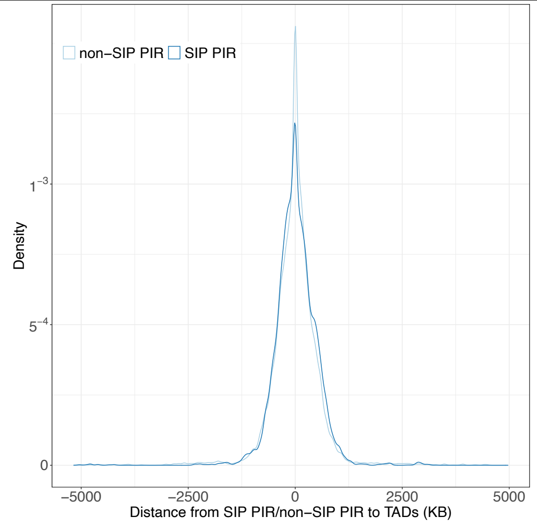

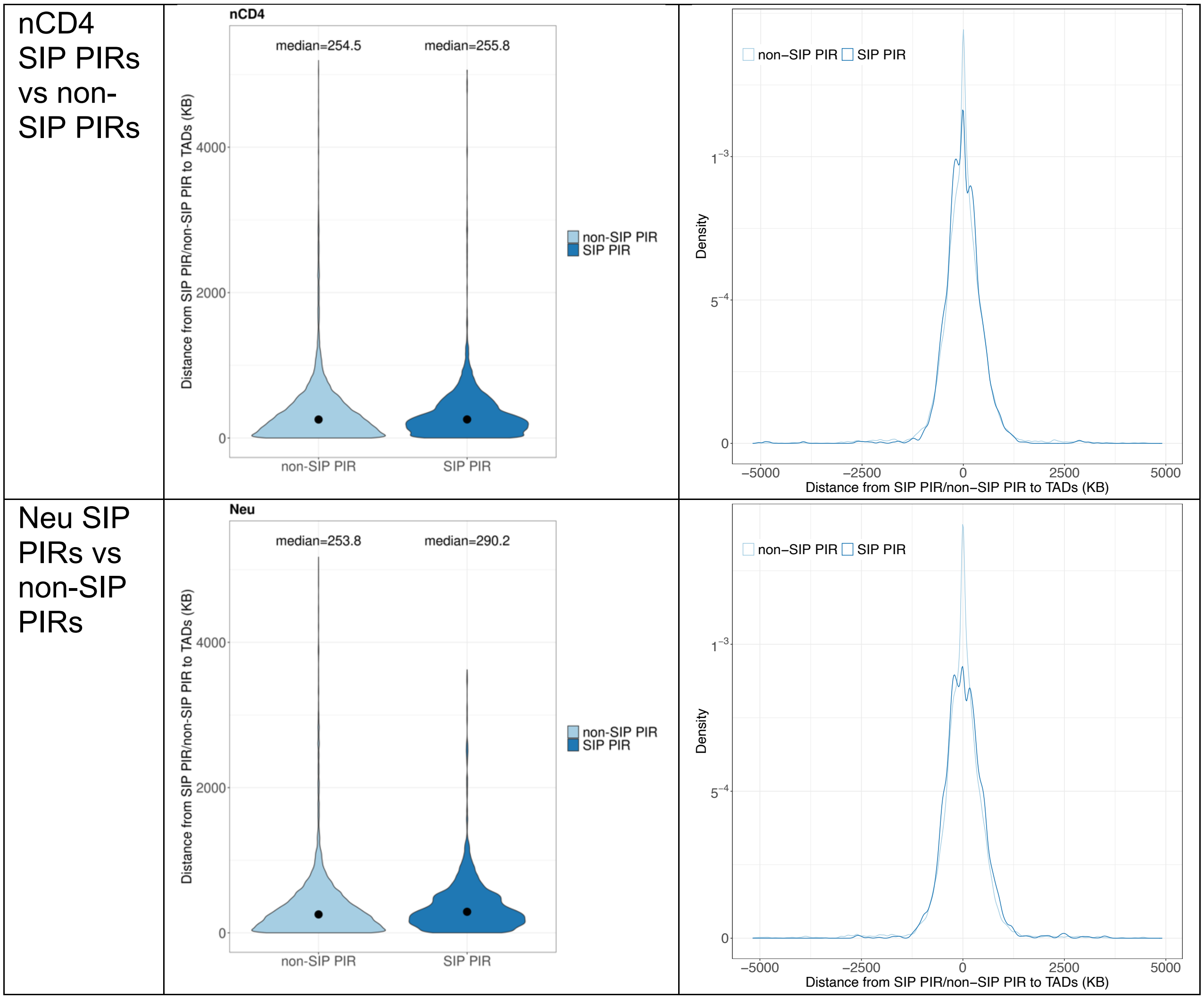

S16 Fig. Distance to TAD boundary for SIP PIRs versus non-SIP PIRs. Distributions of distance to TAD boundaries for non-SIP PIRs versus SIP PIRs are visualized using side-by-side violin plots (left) and overlapping density plots (right).
