# Supplementary material for: Super interactive promoters provide insight into cell type-specific regulatory networks in blood lineage cell types
Source: PLoS Genet. 2022 Jan 31;18(1):e1009984. doi: 10.1371/journal.pgen.1009984 (PMC8830683; doi:10.1371/journal.pgen.1009984)
Supplement: S17 Fig — A. SIPs versus non-SIPs; B. SIP PIRs versus non-SIP PIRs. The median of each distribution is marked by a black dot. (PDF) [file pgen.1009984.s019.pdf]

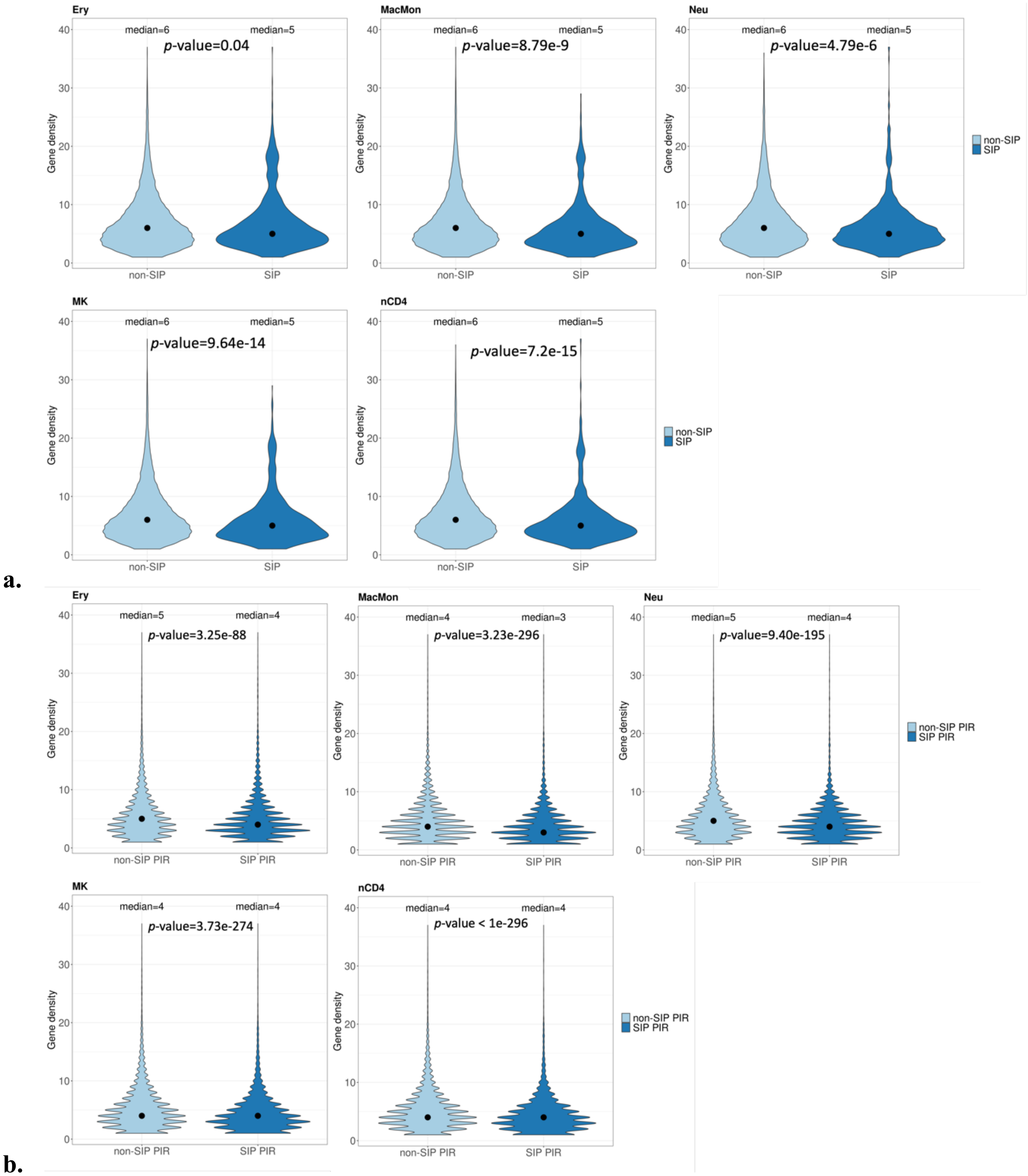

S17 Fig. Gene density for SIPs versus non-SIPs and SIP PIRs versus non-SIP PIRs. A. SIPs versus non-SIPs; B. SIP PIRs versus non-SIP PIRs. The median of each distribution is marked by a black dot.
