# Supplementary material for: Super interactive promoters provide insight into cell type-specific regulatory networks in blood lineage cell types
Source: PLoS Genet. 2022 Jan 31;18(1):e1009984. doi: 10.1371/journal.pgen.1009984 (PMC8830683; doi:10.1371/journal.pgen.1009984)
Supplement: S18 Fig — A. Two replicates of GM12878 HiChIP data; and B. Three replicates of K562 HiChIP data. (PDF) [file pgen.1009984.s020.pdf]

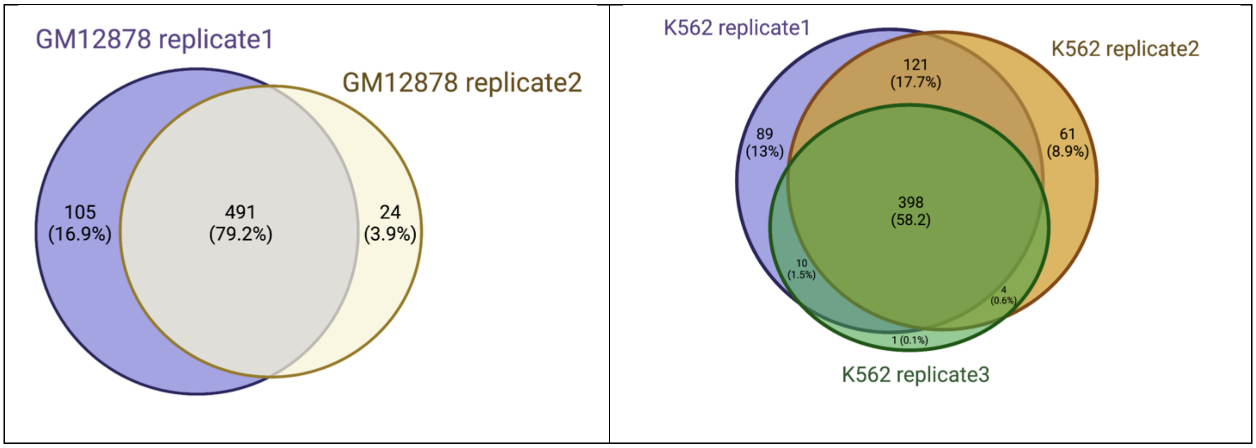

S18 Fig. Venn diagrams show SIPs detected across biological replicates. (A) Two replicates of GM12878 HiChIP data; and **(B)** Three replicates of K562 HiChIP data.
