# Supplementary material for: Super interactive promoters provide insight into cell type-specific regulatory networks in blood lineage cell types
Source: PLoS Genet. 2022 Jan 31;18(1):e1009984. doi: 10.1371/journal.pgen.1009984 (PMC8830683; doi:10.1371/journal.pgen.1009984)
Supplement: S20 Fig — A. The distribution of the number of significant interactions (log10 scale) for SIPs and non-SIPs, merging the two biological replicates; B-C. The distribution of the number of significant interactions (log10 scale) for SIPs and non-SIPs, for each biological replicate; D. The distribution of the median SIP score (-log10 MAPS q-value) of significant interactions for SIPs and non-SIPs, merging the two biological replicates; E-F. The distribution of the median SIP score (-log10 MAPS q-value) of significant interactions for SIPs and non-SIPs, for each biological replicate. (PDF) [file pgen.1009984.s022.pdf]

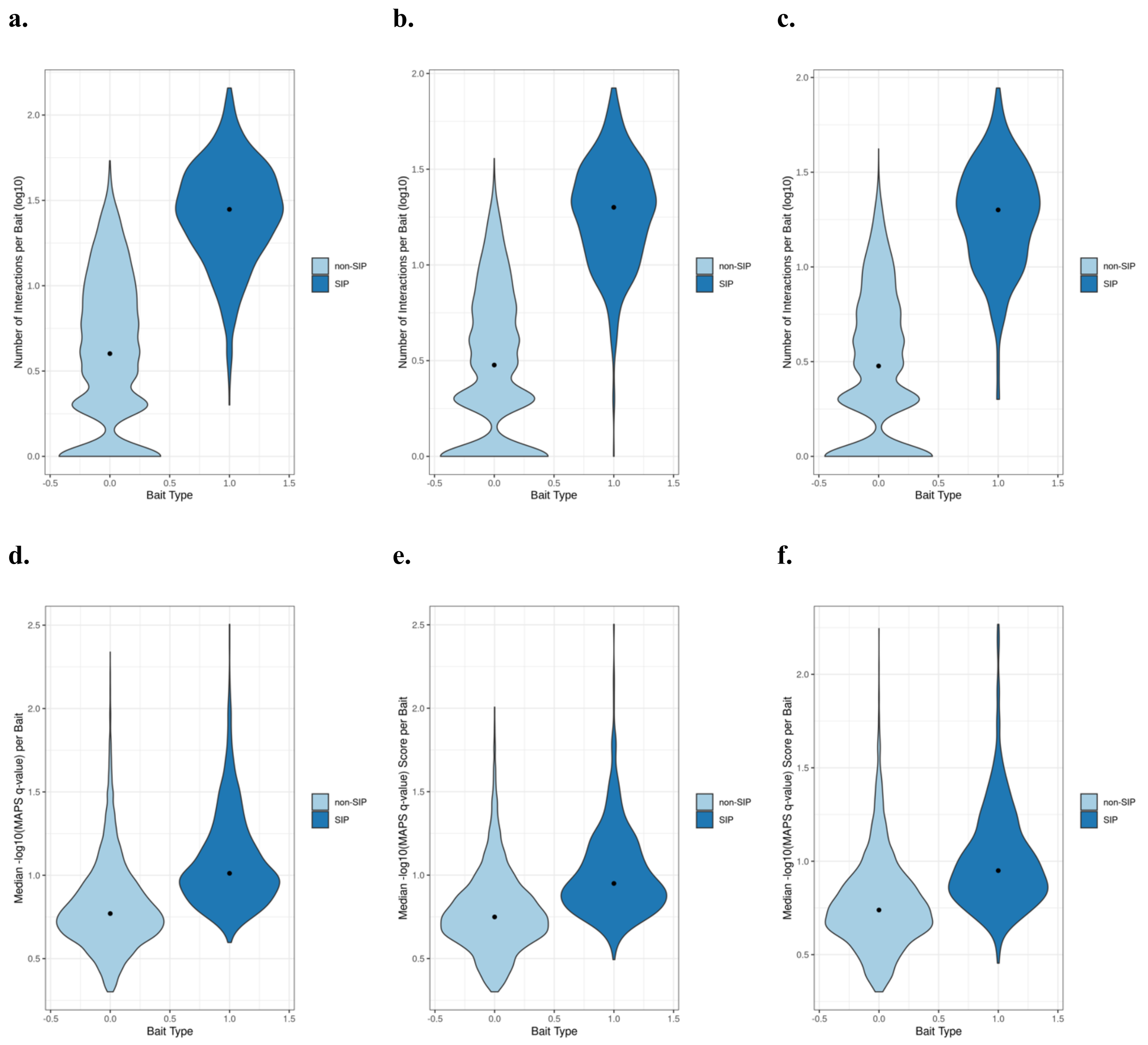

S20 Fig. SIP patterns at different sequencing depths (GM12878 HiChIP data) (A) The distribution of the number of significant interactions (log10 scale) for SIPs and non-SIPs, merging the two biological replicates; (**B-C)**. The distribution of the number of significant interactions (log10 scale) for SIPs and non-SIPs, for each biological replicate; **(D)** The distribution of the median SIP score (-log10 MAPS q-value) of significant interactions for SIPs and non-SIPs, merging the two biological replicates; (**E-F)**. The distribution of the median SIP score (-log10 MAPS q-value) of significant interactions for SIPs and non-SIPs, for each biological replicate.
