# Supplementary material for: Super interactive promoters provide insight into cell type-specific regulatory networks in blood lineage cell types
Source: PLoS Genet. 2022 Jan 31;18(1):e1009984. doi: 10.1371/journal.pgen.1009984 (PMC8830683; doi:10.1371/journal.pgen.1009984)
Supplement: S21 Fig — A. The distribution of the number of significant interactions (log10 scale) for SIPs and non-SIPs, merging the three biological replicates; B-D. The distribution of the number of significant interactions (log10 scale) for SIPs and non-SIPs, for each biological replicate; E. The distribution of the median SIP score (-log10 MAPS q-value) of significant interactions for SIPs and non-SIPs, merging the three biological replicates; F-H. The distribution of the median SIP score (-log10 MAPS q-value) of significant interactions for SIPs and non-SIPs, for each biological replicate. (PDF) [file pgen.1009984.s023.pdf]

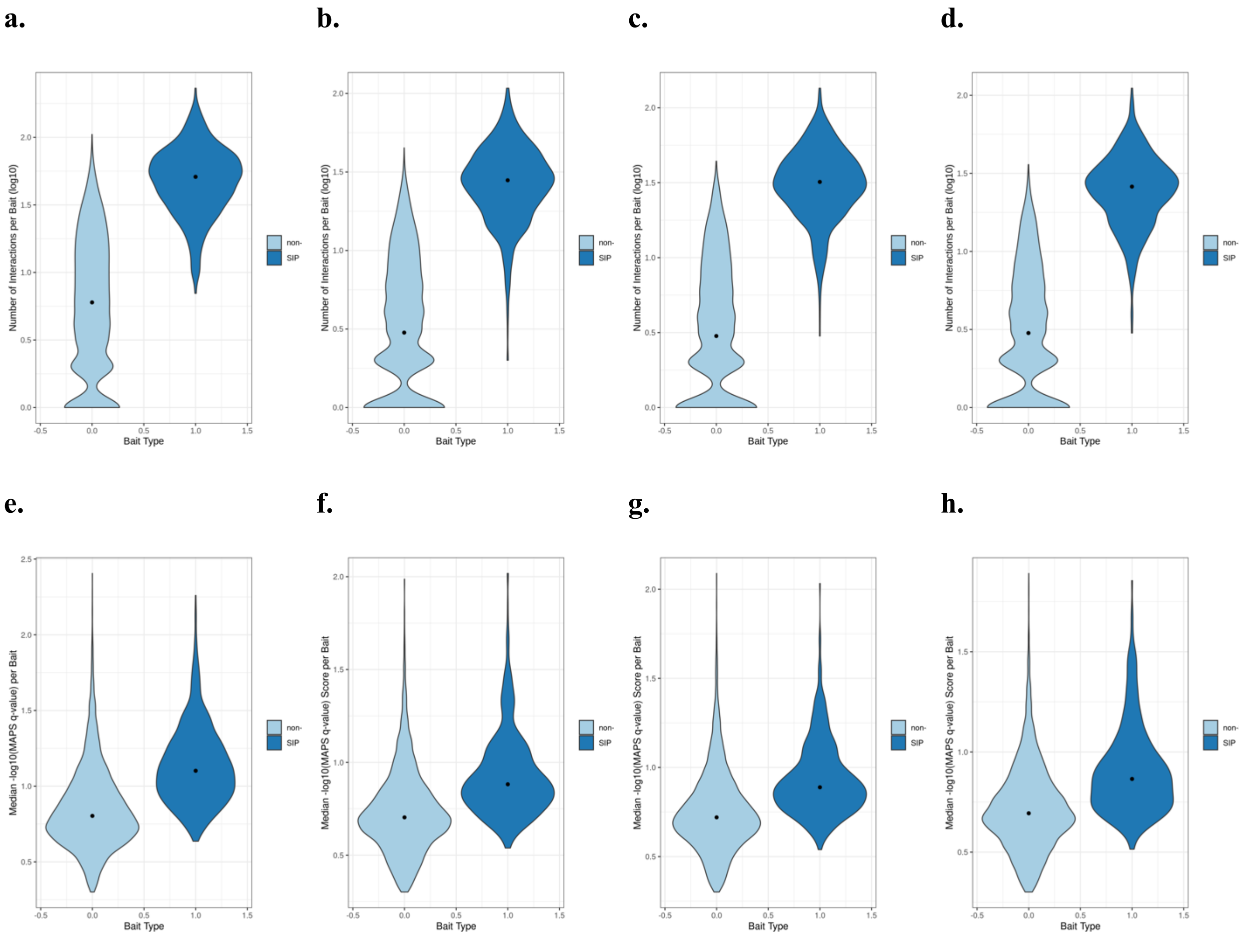

S21 Fig. SIP patterns at different sequencing depths (K562 HiChIP data) (A). The distribution of the number of significant interactions (log10 scale) for SIPs and non-SIPs, merging the three biological replicates; **(B-D)**. The distribution of the number of significant interactions (log10 scale) for SIPs and non-SIPs, for each biological replicate; **(E)**. The distribution of the median SIP score (-log10 MAPS q-value) of significant interactions for SIPs and non-SIPs, merging the three biological replicates; **(F-H)**. The distribution of the median SIP score (-log10 MAPS q-value) of significant interactions for SIPs and non-SIPs, for each biological replicate.
