# Supplementary material for: Super interactive promoters provide insight into cell type-specific regulatory networks in blood lineage cell types
Source: PLoS Genet. 2022 Jan 31;18(1):e1009984. doi: 10.1371/journal.pgen.1009984 (PMC8830683; doi:10.1371/journal.pgen.1009984)
Supplement: S22 Fig — A. The distribution of the number of significant interactions (log10 scale) for SIPs and non-SIPs; B. The distribution of the median CHiCAGO (log10 scale) of significant interactions per promoter bait for SIPs and non-SIPs. (PDF) [file pgen.1009984.s024.pdf]

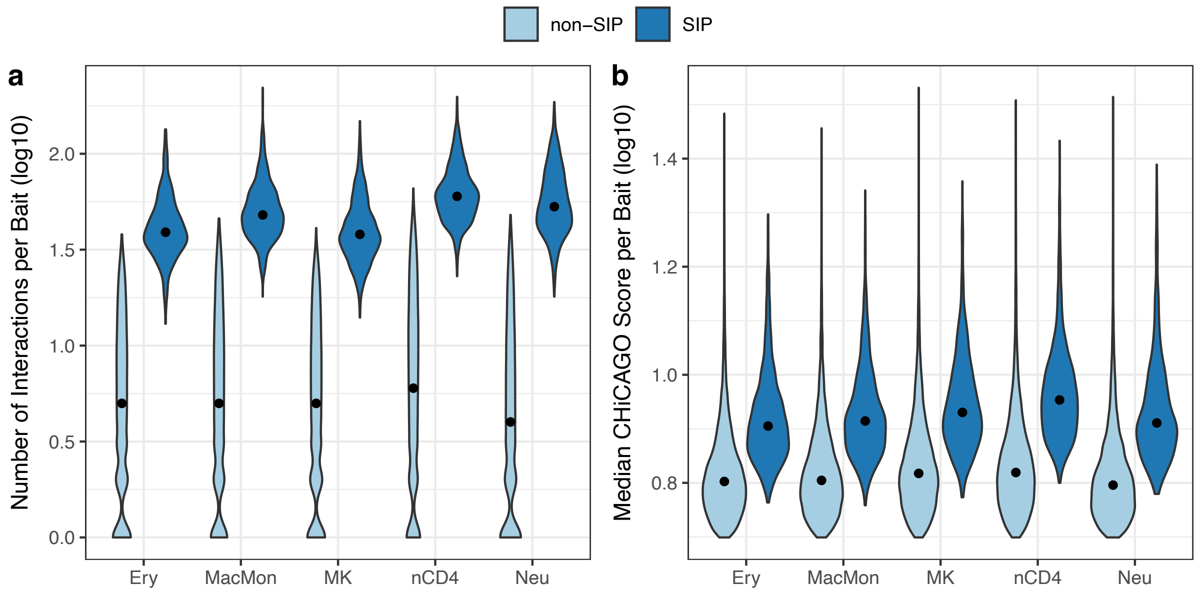

S22 Fig. SIP patterns when detected using a maximum 1D genomic distance of 1Mb (Javierre et al pcHi-C data) (A) The distribution of the number of significant interactions (log10 scale) for SIPs and non-SIPs**; (B)**. The distribution of the median CHiCAGO (log10 scale) of significant interactions per promoter bait for SIPs and non-SIPs.
