# Supplementary material for: Super interactive promoters provide insight into cell type-specific regulatory networks in blood lineage cell types
Source: PLoS Genet. 2022 Jan 31;18(1):e1009984. doi: 10.1371/journal.pgen.1009984 (PMC8830683; doi:10.1371/journal.pgen.1009984)
Supplement: S23 Fig — Dots denote median proportions, and triangles denote mean proportions. (PDF) [file pgen.1009984.s025.pdf]

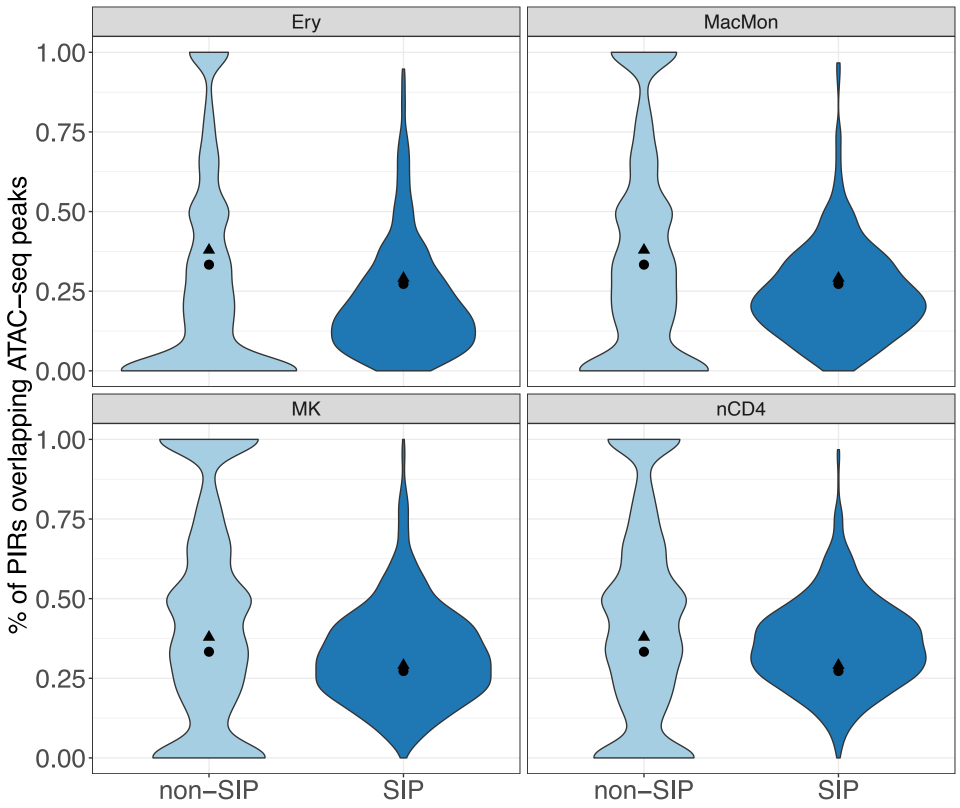

S23 Fig. Proportion of PIRs overlapping ATAC-seq peaks, SIPs versus non-SIPs. Dots denote median proportions, and triangles denote mean proportions.
