# Supplementary material for: Super interactive promoters provide insight into cell type-specific regulatory networks in blood lineage cell types
Source: PLoS Genet. 2022 Jan 31;18(1):e1009984. doi: 10.1371/journal.pgen.1009984 (PMC8830683; doi:10.1371/journal.pgen.1009984)
Supplement: S24 Fig — “0/0” denotes homozygotes; “0/1” denotes heterozygotes; and “1/1” denotes alternative alleles homozygotes. (PDF) [file pgen.1009984.s026.pdf]

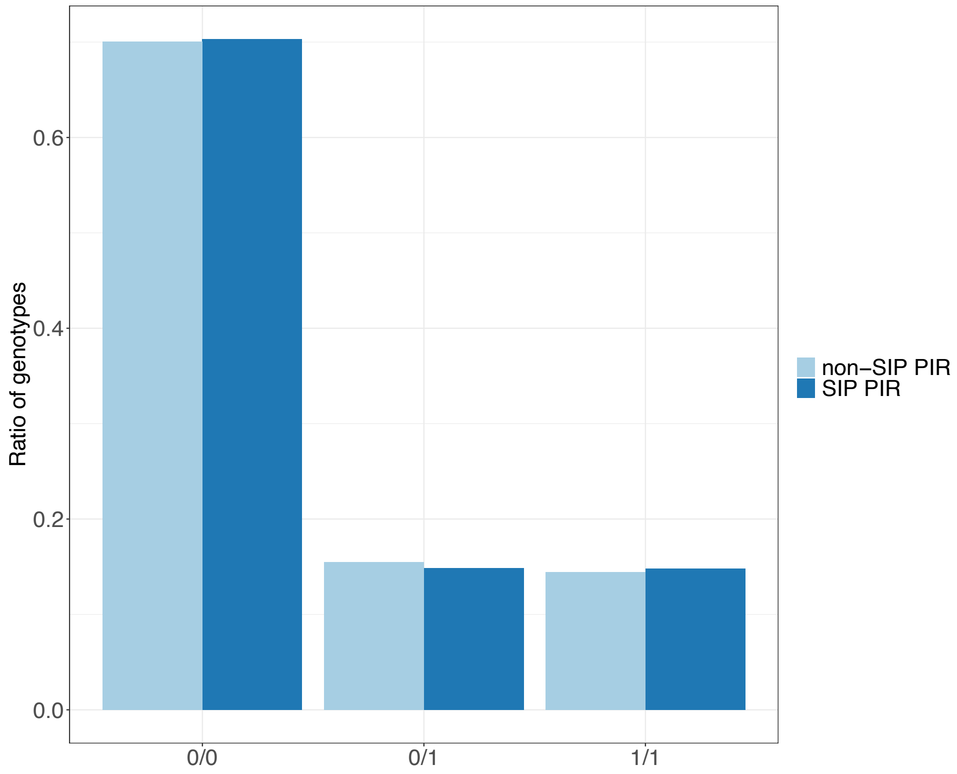

S24 Fig. The K562 genotypes comparison for SIP PIRs and non-SIP PIRs. “0/0” denotes homozygotes; “0/1” denotes heterozygotes; and “1/1” denotes alternative alleles homozygotes.
