# Supplementary material for: Super interactive promoters provide insight into cell type-specific regulatory networks in blood lineage cell types
Source: PLoS Genet. 2022 Jan 31;18(1):e1009984. doi: 10.1371/journal.pgen.1009984 (PMC8830683; doi:10.1371/journal.pgen.1009984)
Supplement: S25 Fig — Effect sizes are from CRISPRi experiments in Gasperini et al [1]. Dots denote median, and triangles denote mean. (PDF) [file pgen.1009984.s027.pdf]

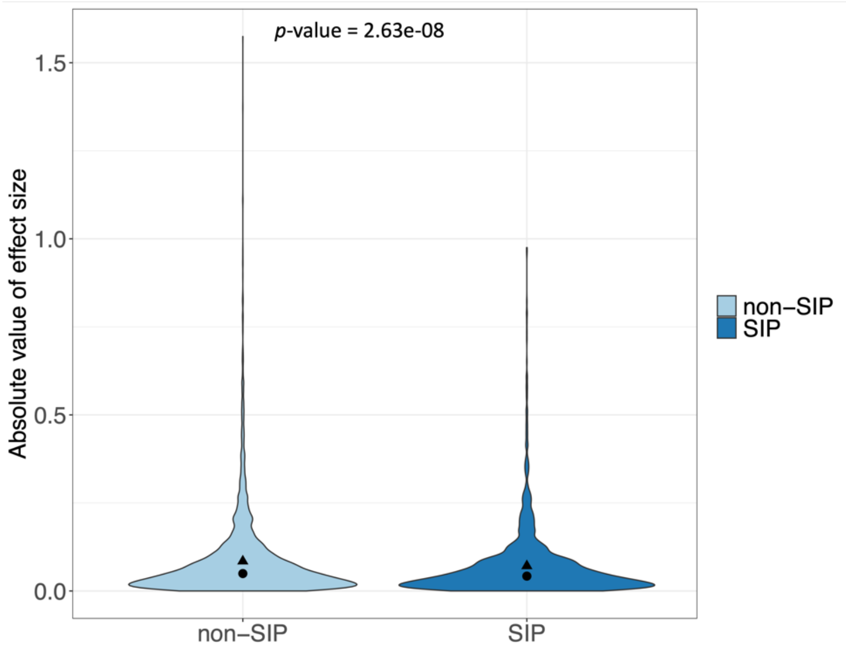

S25 Fig. Effect size when perturbing PIRs, SIPs versus non-SIPs. Effect sizes are from CRISPRi experiments in Gasperini et al[1]. Dots denote median, and triangles denote mean.

Reference

1. Gasperini M, Hill AJ, McFaline-Figueroa JL, Martin B, Kim S, Zhang MD, et al. A Genome-wide Framework for Mapping Gene Regulation via Cellular Genetic Screens. Cell. 2019;176(1-2):377-90 e19. Epub 2019/01/08. doi: 10.1016/j.cell.2018.11.029. PubMed PMID: 30612741; PubMed Central PMCID: PMCPMC6690346.
