# Supplementary material for: Super interactive promoters provide insight into cell type-specific regulatory networks in blood lineage cell types
Source: PLoS Genet. 2022 Jan 31;18(1):e1009984. doi: 10.1371/journal.pgen.1009984 (PMC8830683; doi:10.1371/journal.pgen.1009984)
Supplement: S1 Table — The total number of SIPs and SIP genes shared across all five cell types is also reported. Percent refers to the percent of total SIPs or SIP genes that are cell type-specific. (PDF) [file pgen.1009984.s028.pdf]

S1 Table. Total counts of cell type-specific SIPs and SIP genes as well as total counts of all SIPs and SIP genes, in each cell type. The total number of SIPs and SIP genes shared across all five cell types is also reported. Percent refers to the percent of total SIPs or SIP genes that are cell type-specific.

**SIPs**			
	# Specific	# Total	Percent
Erythrocyte	189	1,157	16.3%
Macrophage/MonocytI 808	13.2%		
Megakaryocyte	302	1,287	23.4%
Naive CD4 T-cell	283	993	28.5%
Neutrophil	274	861	31.8%
Shared	*NA*	170	*NA*
**SIP Genes**			
	# Specific	# Total	Percent
Erythrocyte	251	1,615	15.6%
Macrophage/MonoI125 1,093	11.4%		
Megakaryocyte	385	1,752	22.0%
Naive CD4 T-cell	386	1,393	27.7%
Neutrophil	384	1,201	32.0%
Shared	*NA*	234	*NA*
