# Supplementary material for: Super interactive promoters provide insight into cell type-specific regulatory networks in blood lineage cell types
Source: PLoS Genet. 2022 Jan 31;18(1):e1009984. doi: 10.1371/journal.pgen.1009984 (PMC8830683; doi:10.1371/journal.pgen.1009984)
Supplement: S2 Table — Details corresponding to Fig 2. For each cell type, the number of promoter baits interacting with a super PIR or typical PIR are reported, along with the corresponding ratio and Chi-square p-value. Median PIR scores for each cell type and promoter bait type are also reported, along with the corresponding Wilcoxon p-value for the difference in distribution between SIPs and non-SIPs. (Ery = erythrocytes; MacMon = macrophages/monocytes; MK = megakaryocytes; nCD4 = naive CD4 T-cells; Neu = neutrophils). (PDF) [file pgen.1009984.s029.pdf]

S2 Table. SIPs interact with more super promoter-interacting regulatory regions (super PIRs) than non-SIPs. Details corresponding to **Fig. 2**. For each cell type, the number of promoter baits interacting with a super PIR or typical PIR are reported, along with the corresponding ratio and Chi-square p-value. Median PIR scores for each cell type and promoter bait type are also reported, along with the corresponding Wilcoxon p-value for the difference in distribution between SIPs and non-SIPs. (Ery = erythrocytes; MacMon= macrophages/monocytes; MK = megakaryocytes; nCD4 = naive CD4 T-cells; Neu = neutrophils)

	Bait Type	# Super PIR	# Typical PIR	Ratio	Chi-sq p-value	Median PIR Score	Wilcoxon p-value
**Ery**							
	SIP	969	188	0.84	2.7e-91	6	1.6e-119
	non-SIP	6,924	6,188	0.53		4	
**MacMon**							
	SIP	603	205	0.75	3.2e-35	5	1.7e-50
	non-SIP	6,550	6,011	0.52		4	
**MK**							
	SIP	955	332	0.74	2.7e-52	5	2.9e-68
	non-SIP	6,796	6,273	0.52		4	
**nCD4**							
	SIP	889	104	0.90	7.7e-50	7	3.2e-69
	non-SIP	8,311	4,146	0.67		5	
**Neu**							
	SIP	716	145	0.83	8.6e-83	6	2.9e-104
	non-SIP	5,399	5,618	0.49		3	
