# Supplementary material for: Super interactive promoters provide insight into cell type-specific regulatory networks in blood lineage cell types
Source: PLoS Genet. 2022 Jan 31;18(1):e1009984. doi: 10.1371/journal.pgen.1009984 (PMC8830683; doi:10.1371/journal.pgen.1009984)
Supplement: S3 Table — Details corresponding to Fig 3. For each cell type, the number of SIP PIRs and non-SIP PIRs that overlap with a cell type-specific ATAC-seq peak region are reported, along with the corresponding ratio and Chi-square p-value. The median (Med.) and average (Avg.) number of PIR interactions overlapping an ATAC-seq peak and corresponding t-test p-value for the difference between SIP and non-SIP PIRs is also reported. (Ovlp. = Overlap; Ery = erythrocyteIcMon = macrophages/monocytes; MK = megakaryocytes; nCD4 = naive CD4 T-cells; Neu = neutrophils). (PDF) [file pgen.1009984.s030.pdf]

S3 Table. SIP PIRs overlap with ATAC-seq peak regions. Details corresponding to **Fig. 3**. For each cell type, the number of SIP PIRs and non-SIP PIRs that overlap with a cell type-specific ATAC-seq peak region are reported, along with the corresponding ratio and Chi-square p-value. The median (Med.) and average (Avg.) number of PIR interactions overlapping an ATAC-seq peak and corresponding t-test *p*-value for the difference between SIP and non-SIP PIRs is also reported. (Ovlp. = Overlap; Ery = erythrocyteIcMon = macrophages/monocytes; MK = megakaryocytes; nCD4 = naive CD4 T-cells; Neu = neutrophils)

Bait Type	# Overlap	# No Overlap	Ratio	Chi-sq p-value	Med. # Ovlp.	Avg. # Ovlp.	t p-value
**Ery**							
SIP	1,118	39	0.97	5.3e-118	8	9.4	1.2e-162
non-SIP	8,255	4,857	0.63		1	1.9	
**MacMon**							
SIP	798	10	0.99	8.9e-52	12	12.8	2.7e-172
non-SIP	9,491	3,070	0.76		2	2.6	
**MK**							
SIP	1,285	2	1.00	4.7e-63	12	13.5	6.3e-292
non-SIP	10,645	2,424	0.81		2	2.9	
**nCD4**							
SIP	992	1	1.00	2.9e-45	22	23.3	2.1e-290
non-SIP	10,315	2,142	0.83		3	4.4	
