# Supplementary material for: Super interactive promoters provide insight into cell type-specific regulatory networks in blood lineage cell types
Source: PLoS Genet. 2022 Jan 31;18(1):e1009984. doi: 10.1371/journal.pgen.1009984 (PMC8830683; doi:10.1371/journal.pgen.1009984)
Supplement: S4 Table — (PDF) [file pgen.1009984.s031.pdf]

S4 Table. Around a third of the GWAS variants residing in the SIP PIR regions disrupt the transcript factor binding motifs.

Cell type	# Variants in SIP PIRs	#(%) disrupting motifs
**Ery**	369	132 (35.8%)
**MacMon**	149	59 (39.6%)
**MK**	249	88 (35.3%)
**nCD4**	222	57(25.7%)
**Neu**	208	69 (33.2%)
