# Supplementary material for: Super interactive promoters provide insight into cell type-specific regulatory networks in blood lineage cell types
Source: PLoS Genet. 2022 Jan 31;18(1):e1009984. doi: 10.1371/journal.pgen.1009984 (PMC8830683; doi:10.1371/journal.pgen.1009984)
Supplement: S5 Table — With applying fisher exact test, SIPs highly enriched for CTCF. K562 ORs = 2.46, p-value < 2.2×10^−16^ and GM12878 ORs = 2.92 and p-value < 2.2×10^−16^. (PDF) [file pgen.1009984.s032.pdf]

S5 Table. Cell-type-specific CTCF Enrichment analysis for SIPs versus non-SIPs in K562 and GM12878. With applying fisher exact test, SIPs highly enriched for CTCF. K562 ORs = 2.46, p-value < 2.2×10^{-16} and GM12878 ORs = 2.92 and p-value < 2.2×10^{-16}

K562	SIP	non-SIP
Overlap with CTCF	540	5071
Non-CTCF	271	6281

GM12878	SIP	non-SIP
Overlap with CTCF	594	4835
Non-CTCF	229	5439
